# Small-molecule activating SIRT6 elicits therapeutic effects and synergistically promotes anti-tumor activity of vitamin D_3_ in colorectal cancer

**DOI:** 10.7150/thno.44043

**Published:** 2020-04-27

**Authors:** Jialin Shang, Zhehui Zhu, Yingyi Chen, Jinglue Song, Yuji Huang, Kun Song, Jie Zhong, Xinyuan Xu, Jiacheng Wei, Chengxiang Wang, Long Cui, Chen-Ying Liu, Jian Zhang

**Affiliations:** 1 State Key Laboratory of Oncogenes and Related Genes, Shanghai Jiao-Tong University School of Medicine, Shanghai 200025, China.; 2 Department of Colorectal and Anal Surgery, Xinhua Hospital, Shanghai Jiao-Tong University School of Medicine, Shanghai 200092, China.; 3 Medicinal Bioinformatics Center, Shanghai Jiao-Tong University School of Medicine, Shanghai 200025, China.; 4 School of Pharmaceutical Sciences, Zhengzhou University, Zhengzhou 450001, China.

**Keywords:** Colorectal cancer, Translational medicine, SIRT6 activator, CYP24A1, Vitamin D_3_

## Abstract

Colorectal cancer (CRC) is the leading cause of cancer death; however, targets with broad anti-CRC effects are limited. Sirtuin6 (SIRT6) is a conserved nicotinamide adenine dinucleotide (NAD^+^)-dependent deacetylase that is widely pathologically downregulated in CRC, but its pharmacological effect in CRC remains undefined due to the lack of small-molecule SIRT6 activators. We searched for a compound activating SIRT6 and investigated its anti-CRC effect in various models.

**Methods**: We identified an allosteric SIRT6 activator, MDL-811. Its ability to enhance SIRT6 deacetylation at protein and cellular levels was evaluated by Fluor de Lys (FDL) and western blots. We assessed the proliferation of 26 CRC cell lines and patient-derived organoids (PDOs) treated with MDL-811. *In vivo* efficacy of MDL-811 was evaluated in HCT116 cell line- and patient-derived xenografts as well as a spontaneous CRC model. RNA sequencing and real-time quantitative PCR assays were performed to analyze gene expression changes in MDL-811-treated HCT116 cells. Along with controls in SIRT6-overexpressing HCT116 cells, the SIRT6-mediated histone H3 deacetylation at the Cytochrome P450 family 24 subfamily A member 1 (CYP24A1) gene locus was assessed by chromatin immunoprecipitation (ChIP) in MDL-811-treated HCT116 cells. A combination therapy against CRC based on the downstream gene of SIRT6 activation was evaluated in cells and mouse models.

**Results**: MDL-811 significantly activated SIRT6 histone H3 deacetylation (H3K9Ac, H3K18Ac, and H3K56Ac) *in vitro* and had broad antiproliferative effects on diverse CRC cell lines and PDOs. More importantly, the *in vivo* anti-tumor efficacy of MDL-811 was demonstrated across cell line- and patient-derived xenografts and in the APC^min/+^ spontaneous CRC model. Mechanically, we identified a new downstream target gene of SIRT6 in CRC, CYP24A1. Based on these findings, a combination drug strategy with MDL-811 to synergistically enhance the anti-CRC effect of vitamin D_3_ was validated *in vitro* and *in vivo*.

**Conclusions**: Our data provide proof of concept that targeting SIRT6 using a small-molecule activator is an attractive therapeutic strategy for CRC and that MDL-811 could be a promising lead compound for further preclinical and clinical studies of treatments for CRC.

## Introduction

Colorectal cancer (CRC) is one of the most common malignant tumors and carries a high risk of morbidity and mortality worldwide 1, 2. Given the disadvantages of conventional therapies, such as toxicity and intolerance, clinical outcomes remain unsatisfactory 2, 3. Thus, there is a pressing need to identify novel drug targets and develop effective therapeutic agents for CRC. In CRC, epigenetic alterations, including histone modifications, have been recognized as a major driver of tumorigenesis and tumor progression 4-7. Sirtuin6 (SIRT6) is a member of the conserved nicotinamide adenine dinucleotide (NAD^+^)-dependent class III histone deacetylase (HDAC) sirtuin family, and emerging evidence implicates SIRT6 in tumorigenesis 8, 9. Many studies suggest that SIRT6 expression has been reduced in many human tumors, including colorectal, pancreatic, ovarian, glioma, hepatocellular, and lung cancers, which is associated with poor clinical outcomes 10-15; Consistently, depletion of SIRT6 results in tumor formation and maintenance, indicating that SIRT6 acts as a tumor suppressor 10. However, some studies suggest an oncogenic role of SIRT6 in skin cancer, squamous cell carcinoma, and acute myeloid leukemia 16-20, therefore the identification of SIRT6 modulators would be crucial for elucidating the pleiotropic effects of SIRT6 in cancer biology, as well as for cancer therapy. The downregulation of SIRT6 closely correlates with carcinogenesis and a poor prognosis in CRC 6, 10, 21, 22. The conditional intestinal deletion of SIRT6 can significantly increase the size, number, and aggressiveness of adenomas in the APC^min/+^ mouse model of spontaneous CRC 10. In addition, SIRT6 overexpression can inhibit CRC stem cell proliferation 23. These findings highlight SIRT6 as a potential drug target for CRC and indicate that its activation may elicit a therapeutic response against CRC.

SIRT6 contains a small zinc-binding domain and a large Rossmann fold domain and forms a large hydrophobic pocket harboring a substrate peptide with an acetylated lysine and NAD^+^, where the acyl group is removed from the lysine to NAD^+^ through deacetylation of SIRT6, yielding nicotinamide, 2′-*O*-acyl-ADP ribose (*O*AADPr), and a deacetylated substrate 24. Previous studies have revealed that SIRT6 is a chromatin-bound protein and deacetylates histone H3 on acetylated K9, K18 and K56 (H3K9Ac, H3K18Ac, and H3K56Ac, respectively) depending on the genomic or cellular context 25-27. SIRT6 thereby acts as a corepressor of oncogenic transcription factors, such as c-Myc 10, Hif-1α 28, and c-Jun 29, and is a tumor suppressor controlling metabolism and tumorigenesis in cancers, including CRC 10, 11, 23, 30. Collectively, these reports indicate that the activation of SIRT6 deacetylase activity could play an important role in the repression of tumorigenesis in CRC.

Due to the potential of SIRT6 activation, many efforts have been directed toward the development of potent activators of SIRT6. Lamin A has been reported as an endogenous activator of SIRT6 to increase deacetylation 31. Recently, several small molecules have been identified to activate the deacetylation of SIRT6 but showed poor efficacy *in vivo* 32-34*.* Therefore, identifying drug-like SIRT6 activators that may be useful in deciphering both the pathological function and potential clinical value of the SIRT6 protein for CRC is desirable.

Here, we report the discovery of a potent SIRT6 activator, MDL-811. MDL-811 activated SIRT6 biochemically in an allosteric manner and is selective for SIRT6 over other HDACs. Using MDL-811 as a pharmacological probe, we found that MDL-811 exhibited high anti-tumor efficacy against CRC in multiple cell-based assays and several *in vivo* models, including cell line-derived and patient-derived xenograft (CDX and PDX, respectively) models, as well as in the genetically engineered APC^min/+^ model of spontaneous CRC. Mechanically, we identified Cytochrome P450 family 24 subfamily A member 1 (CYP24A1) as a new target gene of SIRT6 for the inhibition of CRC proliferation. We then showed that the activation of SIRT6 by MDL-811 suppressed the transcription of CYP24A1, which synergistically enhanced the anti-tumor effect of vitamin D_3_ (VD_3_) in CRC. In summary, our study provides evidence that SIRT6 activation is an effective therapeutic strategy for CRC and that this highly characterized SIRT6 activator represents a valuable lead compound for advancing the understanding of the role of SIRT6 as a target in CRC and developing broad therapeutic agents against CRC.

## Materials and Methods

### Cloning, expression, and purification of wild-type SIRT6

According to previously described methods 34, WT (wild-type) full-length human SIRT6 was inserted into the pET28a-LIC vector (Addgene plasmid #26094). The plasmid was transformed into *Escherichia coli* BL21 (DE3) cells. Protein was purified using a nickel column and gel filtration. Purified protein was dialyzed into assay buffer (50 mM Tris-HCl [pH 8.0], 137 mM NaCl, 2.7 mM KCl, and 1 mM MgCl_2_) and used in all *in vitro* assays performed in this study.

### FDL assay

For the assessment of SIRT6 deacetylase activity, 5 μM WT SIRT6 was incubated in a 50-μL reaction mixture (2.5 mM NAD^+^, 75 μM RHKK-Ac-AMC, compounds/DMSO, and assay buffer) at 37 °C for 2 h, quenched with 40 mM nicotinamide, and developed with 6 mg/mL trypsin for another 30 min at 25 °C. For the assessment of SIRT6 deacylase activity, 1 μM WT SIRT6 was incubated in a 50-μL reaction mixture (1 mM NAD^+^, 7.5 μM EALPKK-Myr-AMC, MDL-811/DMSO, and assay buffer) at 37 °C for 2 h, quenched with 8 mM nicotinamide, and developed with 6 mg/mL trypsin for another 2 h at 37 °C. Fluorescence intensity was measured using a microplate reader (Synergy H4 Hybrid Reader, BioTek) at excitation and emission wavelengths of 360 nm and 460 nm, respectively. EC_50_ values were calculated by fitting the data points with the dose-response function in GraphPad Prism V7 (GraphPad Software). Each experiment was independently repeated three times in technical triplicates.

### Pharmacokinetic studies in mice

Pharmacokinetic studies were performed by Shanghai Medicilon Inc, China, following standard protocols. Briefly, six-week-old male C57BL/6J mice were grouped randomly (n = 5 per group). Five mice of each group were administrated MDL-800/MDL-811 either by a single intravenous (IV) bolus or intraperitoneal (IP) injection at a dose of 20 and 30 mg/kg, respectively. Two administration formulations were prepared in the vehicle with 5% DMSO, 10% Solutol and 85% saline, with the pH adjusted to 7.0-8.0. After treatment, the mice were sacrificed, and their plasma samples were collected at 5 min, 15 min, 30 min, 1 h, 2 h, 4 h, 8 h, and 24 h. The drug concentration in plasma was analyzed by LC-MS/MS. Pharmacokinetic parameters were calculated using Analyst Software 1.6.3 from mean plasma concentration-time profiles. The area under the curve (AUC) was measured from time versus concentration data by the linear trapezoidal rule. The IP bioavailability was calculated as the ratio of AUC for MDL-800/MDL-811 from IP and IV dosage. The calculation was normalized to relative doses.

### Molecular docking

Molecular docking calculations were performed using the crystal structure of SIRT6 in complex with MDL-801 (Protein Data Bank [PDB] code 5Y2F) 34 and prepared with the Protein Preparation Wizard in MAESTRO v11.2.013 (Schrodinger, Inc.). In addition, the compound was processed by the LigPrep module for 3D structure generation, protonation, and energy minimization. Subsequently, each MDL compound was docked into the allosteric site on SIRT6 defined by a 15 × 15 × 15 grid box implemented by GLIDE version 4.5. The best binding mode was selected for further analysis of the structure-activity relationship.

### HPLC assay

For the assessment of SIRT6 deacetylation on RHKK-Ac-AMC, 5 μM WT SIRT6 was incubated in a 50-μL reaction mixture (75 μM RHKK-Ac-AMC, 2.5 mM NAD^+^, and assay buffer) with DMSO or the indicated concentrations of MDL-800/MDL-811 at 37 °C for 2 h. Reactions were terminated with 100 mM HCl and 160 mM acetic acid. After centrifugation at 12,000 r.p.m. for 10 min, the supernatant was analyzed by high-performance liquid chromatography (HPLC) using a Zorbax Eclipse Plus C18 column (4.6 × 100 mm, 3.5 μM). The solvents used for HPLC were water with 0.1% trifluoroacetic acid (solvent A) and acetonitrile with 0.1% trifluoroacetic acid (solvent B). Each experiment was independently repeated three times.

### Histone deacetylase enzyme selectivity assay for MDL-811

The selectivity assays of MDL-811 on histone deacetylase enzymes (HDAC1-11, SIRT1-3, and SIRT6) were performed by CEREP company (Eurofins Cerep, France) according to CEREP standard protocols. In brief, MDL-811 or DMSO control was preincubated for 5 min at room temperature with the corresponding recombinant enzyme at the indicated concentrations in a 20-μL reaction mixture (45 mM Tris-HCl [pH 8.0], 123.3 mM NaCl, 2.43 mM KCl, 0.9 mM MgCl_2_ and 0.18% BSA). Then, the reaction was initiated by adding the fluorogenic HDAC acetylated substrate (also adding NAD^+^ in the deacetylase assays of sirtuins) at the indicated concentrations at room temperature or 37 °C for the indicated durations. After incubation, the reaction was stopped with an equal volume of buffer containing 1 mL of HDAC developer (peptidase activity, BPS Bioscience); 20 µM trichostatin was also included for the termination in the deacetylase assays of HDAC1-11. After the indicated time, fluorescence intensity was measured using a microplate reader (Envision, Perkin Elmer) at excitation and emission wavelengths of 355 nm and 460 nm, respectively. We also developed deacetylase assays for SIRT5 and SIRT7, which were not available at CEREP. For SIRT5 deacetylation assay, HEK293T cells were transiently transfected with empty vector or pcDNA3.1-SIRT5-HA (10 μg/10 cm plate) using Neofect™ DNA transfection reagent. 48 h after transfection, cells were lysed in ice-cold NP-40 buffer (50 mM Tris-HCl [pH 8.0], 150 mM NaCl, 1% NP-40, and protease inhibitor cocktail) with rotation at 4 °C for 30 min. A particle-free supernatant solution was obtained by centrifugation at 13,600 × g for 15 min at 4 °C. Immunoprecipitation was carried out by incubating anti-HA magnetic beads (20 μL/ per 10 cm plate, Pierce-88836, Thermo Scientific) at 4 °C with the lysate supernatant for 2 h. Beads were washed three times with ice-cold NP-40 buffer. HA-peptide (HY-P0239, MedChemExpress) was diluted with assay buffer to the final concentration of 2 mg/mL. Then, HA-SIRT5 protein was eluted with HA-peptide dissolved in the assay buffer at 4 °C and used for the deacetylase assay. The purification procedures of the full-length SIRT7 protein were the same as previously described 35. The methods for the deacetylase assays of the HA-SIRT5 protein and the full-length SIRT7 protein were the same as those for SIRT6 deacetylation as previously described 34. The data points at various concentrations were expressed as percent inhibition or activation effects of the control activity. The data were from three independent experiments conducted in technical triplicates.

### Immunoblotting and antibodies

Western blot analysis was performed as described previously 34. Cell lines (3 × 10^5^ cells) were seeded in 6-well plates and treated with DMSO or MDL-811; the final concentration of DMSO in the media was 0.1%. After 48 h of treatment, the cells were lysed with 1× SDS and separated by SDS-PAGE, followed by incubation with specific primary antibodies overnight at 4 °C and then with HRP-linked anti-rabbit IgG (#7074, Cell Signaling Technology) or HRP-linked anti-mouse IgG (#7076, Cell Signaling Technology). An Immobilon Western Chemiluminescent HRP Substrate Kit (Merck Millipore) was used for detection. Information regarding the primary antibodies can be found in Table S6. Quantification of western blot data was performed using ImageJ V4.

### Detection of SIRT6 deacetylation on nucleosome substrates

*In vitro* deacetylation reactions on nucleosome substrates were performed as previously reported 26, 32. Briefly, 5 μg of mononucleosomes purified from HeLa cells with a nucleosome preparation kit (Active Motif) and 0.2 μg of purified WT SIRT6 were incubated in 40 μL of HDAC buffer (50 mM Tris-HCl [pH 8.0], 2 mM NAD^+^, and 150 mM NaCl) for 80 min at 30 °C. The indicated concentrations of MDL-811 or DMSO control were added before incubation. The reaction mixture was analyzed by western blotting.

### Cell culture

The HEK293T, HeLa, HT29, HCT116, SW480, LS174T, RKO, Colo205, SW620, and DLD-1 cell lines were obtained from the Cell Resource Center of the Shanghai Institute for Biological Sciences, Chinese Academy of Sciences. The HCT15, Colo201, Colo320DM, SW1116, Caco2, T84, NCI-H716, HCT8, and LOVO cell lines were purchased from the EK-Bioscience Biotechnology Company Limited Shanghai Enzyme Research. The LS123, SKCO1, LS180, LS1034, LS513, SW1463, SW1417, SW48, NCI-H508, and FHC cell lines were purchased from CELLBIO, Saiqi (Shanghai) Biological Engineering Company Limited. HEK293T, HeLa, Caco2, T84, LS123, SKCO1, LS180, LS174T, and RKO cell lines were maintained in Dulbecco's modified Eagle's medium (DMEM) supplemented with 10% (v/v) fetal bovine serum (FBS) (Gibco). SW480, HCT15, Colo201, Colo320DM, NCI-H716, HCT8, LS1034, LS513, Colo205, SW620, DLD-1, and NCI-H508 cells were maintained in RPMI 1640 medium added with 10% (v/v) FBS. HT29, and HCT116 cell lines were maintained in McCoy's 5A modified medium supplemented with 10% (v/v) FBS. SW1116, SW1463, SW1417, and SW48 cell lines were maintained in L-15 medium supplemented with 10% (v/v) FBS. LOVO cells were maintained in F-12K medium supplemented with 10% (v/v) FBS. FHC cells were maintained in DMEM:F12 medium with 10 mM HEPES (for a final concentration of 25 mM), 10 ng/mL cholera toxin, 0.005 mg/mL insulin, 0.005 mg/mL transferrin, 100 ng/mL hydrocortisone, 20 ng/mL human recombinant EGF (Thermo Fisher, PHG0311), and 10% (v/v) FBS. All cell lines were cultured according to the standard cell culturing protocols of American Type Culture Collection and maintained in a humidified 37 °C incubator with 5% CO_2_. However, cells cultured in L-15 medium were maintained in free gas exchange with atmospheric air at 37 °C. The cell lines were authenticated by short tandem repeat (STR) profiling, as previously described 36.

### Cellular thermal shift assay (CETSA)

CETSA was conducted as previously described 37. HCT116 cells were seeded in 10-cm cell culture dishes and grown to ~90% confluence. Subsequently, the cells were treated with DMSO or MDL-811 (10 μM) for 24 h; the final DMSO concentration in the assay was 0.1%. Cell pellets were collected by centrifugation (1,000 × g, 5 min, 4 °C), resuspended in 600 μL of 1× PBS, and distributed into different 0.2-mL tubes, each with 40 μL of cell suspension. The tubes were heated at the indicated temperature endpoints (38.5-51.5 °C) for 3 min in a PCR thermocycler. Immediately after heating, the tubes were incubated at room temperature for another 3 min. The samples were subjected to two freeze-thaw cycles in liquid nitrogen. Each cell lysate was centrifuged (15,000 × g for 20 min at 4 °C), and 25 μL of supernatant was collected. The supernatants were lysed in 25 μL of 2× SDS loading buffer at 95 °C for 10 min. Then, the samples were centrifuged, and the supernatants were analyzed by western blotting. Quantification of the SIRT6 level was performed with ImageJ V4. The signal intensity of each protein band on the western blots was normalized to the signal intensity observed at 38.5 °C. The corresponding signal intensities were plotted against temperature in GraphPad Prism V7. Each data point is shown as the mean ± s.e.m. of four independent experiments.

### Cell proliferation

Cell proliferation was evaluated using a Cell Counting Kit-8 (CCK-8) (Dojindo) following the manufacturer's instructions. Briefly, CRC cells were plated in 96-well plates at a density of 5 × 10^3^ cells per well. After attachment overnight, CRC cells were treated with MDL-811 at various concentrations for 48 h. Cells treated with DMSO were used as the positive controls, and the final DMSO concentration in the medium was 0.1%. After 48 h of treatment, 10 μL of CCK-8 solution was added to every well and incubated for 1 h at 37 °C. Then, a microplate reader (Synergy H4 Hybrid Reader, BioTek) was used to measure the absorbance of the solvent at 450 nm. The background of drug and culture medium has been deducted in each well. The relative cell proliferation of each treatment group was expressed as the percentage change relative to the positive control group. Each data point is the mean ± s.d. of two or three independent experiments.

### Calcein-AM/propidium iodide (PI) double staining

Live-dead cell staining was performed following the protocol of a Calcein-AM/PI Double Stain Kit (Yeasen). Briefly, 3 × 10^4^ cells per well were seeded into 24-well plates. After treatment with the indicated concentrations of MDL-811 or doxorubicin for the indicated times (the final DMSO concentration in the medium was 0.1%), the cells were resuspended and incubated with Calcein-AM and propidium iodide for 15 min at 37 °C. Then, cell images were acquired with a fluorescence microscope (Nikon Inverted Research Microscope Eclipse Ti). For each assay, five random fields of view per well were imaged at 10× magnification.

### LDH assay

Cell cytotoxicity was assessed by measuring LDH release from damaged or dead cells using a Cytotoxicity LDH Assay Kit-WST (Dojindo) following the manufacturer's instructions. Briefly, 5 × 10^3^ cells per well were plated in 96-well plates. Twelve hours after seeding, the cells were treated with various concentrations of MDL-811 for 48 h or with 10 μM doxorubicin for 12 h. The final DMSO concentration in the medium was 0.1%. The amount of LDH leakage from the cells was determined by measuring the absorbance at 490 nm using a microplate reader (Synergy H4 Hybrid Reader, BioTek). The percentage of cytotoxicity was calculated as follows: [(test sample - low control)/ (high control - low control)] × 100, where low control is the absorbance of the culture medium without any added extract, and high control is the absorbance of the culture medium in which the cells were treated with 1% (v/v) Triton X-100 to achieve 100% LDH release. Each data point is presented as the mean ± s.d. of three independent experiments.

### Cell cycle analysis

Cells were seeded in 6-well plates overnight and treated with DMSO or with 5 or 10 μM MDL-811 for 48 h; the final DMSO concentration in the media was 0.1%. Then, cells were collected and fixed with 75% ethanol at 4 °C overnight. The cells were incubated with RNase I (50 μg/mL) in 1× PBS at 37 °C for 30 min and stained with propidium iodide (50 μg/mL) for 15 min. The cells were analyzed by flow cytometry and FACS DIVA software, v6.2 (BD Biosciences). The cell cycle distribution was analyzed using FlowJo 7.6.1 software. The data are presented as the mean ± s.d. of three independent experiments.

### Clinical sample collection

All human CRC tissues were collected in the Department of Colorectal and Anal Surgery, Xinhua Hospital, Shanghai Jiao-Tong University School of Medicine. Tumor tissues were collected from four independent donors. All clinical sample collections were approved by the ethics committee of Xinhua Hospital Affiliated with Shanghai Jiao-Tong University School of Medicine. Informed consent was obtained from all patients providing clinical samples.

### CellTiter-Glo 3D viability assay

Tumor cell isolation, organoid culture, and organoid viability assays were performed as previously described 38. CRC PDOs were plated (n = 2 wells/group) in 96-well plates. After 24 h, the media were replaced with DMSO- or MDL-811-containing Matrigel. 3D organoid viability was quantified using CellTiter-Glo (Promega) after 72 h of incubation. Viability was normalized to that of the DMSO groups.

### Human CRC CDX and PDX studies

All animal studies were performed following federal and institutional guidelines approved by the Institutional Animal Care and Use Committee at Shanghai Jiao-Tong University School of Medicine. To establish the CDXs, HCT116 cells (1 × 10^6^) suspended in 100 μL of 1× PBS were injected subcutaneously into the flanks of six-week-old male BALB/c nude mice. To establish the PDXs, primary tumors resected from two independent CRC patients (patient #1 and #2) were collected in the culture medium. The necrotic and supporting tissues were carefully removed using sterilized surgical blades. The gross tumor was cut into fragments (2-3 mm in diameter) and inoculated subcutaneously into the flanks of nude mice. The mice were monitored daily, and caliper measurements began when tumors became visible. The tumor volume was monitored every other day via calipers. The tumor volume was calculated using the following formula: (L × W ^2^) / 2, where L and W refer to the tumor diameter along the longitudinal and transverse axes, respectively. When the mean tumor volume was approximately 100 to 250 mm^3^, the mice were randomly grouped (n = 6 per group for CDXs, n = 8 per group for PDXs), and treatment was initiated. The mice received an intraperitoneal injection of the vehicle alone (5% DMSO, 30% PEG-400, and 65% saline, pH 7.0-8.0) or designated doses of MDL-811 every other day for 16 days for CDXs and for 12 days for PDXs. After treatment or if meeting the humane endpoint criteria, the mice were sacrificed by CO_2_ asphyxiation. The tumors were dissected, imaged, and weighed. Tumor tissues were collected, fixed in 10% neutral buffered formalin, or snap frozen in liquid N_2_ and stored at -80 °C for subsequent analyses.

### APC^min/+^ mouse model

C57BL/6J-APC^min/+^ mice were obtained from Jackson Laboratory. Eight-week-old male mice of the same parental generation were divided into two groups that received 20 mg/kg MDL-811 or vehicle (5% DMSO, 30% PEG-400, and 65% saline, pH 7.0-8.0) via intraperitoneal injection every two days for 12 weeks. The mice were sacrificed in the twentieth week. Tumor numbers were calculated, and tumors were imaged after longitudinal dissection of the small intestine and colon. Then, the small intestines and colons were sectioned and stained for immunohistochemistry.

### Histological and immunohistochemical analyses

Xenograft tumor tissues were fixed in 4% paraformaldehyde for 24 h, embedded in paraffin, and sectioned with a semiautomated rotary microtome (Leica, RM2235). Morphology was evaluated by hematoxylin and eosin (H&E) staining. The protein levels of H3K9Ac and Ki67 were analyzed by immunohistochemistry according to standard protocols with specific primary antibodies (anti-H3K9Ac, 1:100 dilution, ab32129, Abcam; anti-Ki67, 1:200 dilution, ab15580, Abcam). All assays were followed by staining with HRP-conjugated rabbit secondary antibody (KIT-9707, Maixin Biotechnology), and samples were then counterstained with hematoxylin and mounted. Images were captured with a Nikon Ti-S microscope.

### Transcriptome profiling

RNA purification, reverse transcription, library construction, and sequencing were performed at WuXi NextCODE in Shanghai following the manufacturer's instructions (Illumina). Total RNA was extracted from 5 × 10^6^ HCT116 cells treated in triplicate with DMSO or 10 μM MDL-811 for 48 h. Samples containing approximately 500 ng of high-quality RNA (OD_260/280_ = 1.9-2.0, RIN ≥ 8) were used to construct the sequencing library. The mRNA-focused sequencing libraries were prepared from total RNA using an Illumina TruSeq® RNA sample preparation kit. PolyA mRNA was purified from total RNA using oligo dT-conjugated magnetic beads and fragmented in fragmentation buffer. These short fragments were used as templates for synthesis of first-strand cDNA using reverse transcriptase and random primers, followed by synthesis of second-strand cDNA. Then, the synthesized cDNA was subjected to end repair, phosphorylation and 'A' base addition according to Illumina's library construction protocol. Then, Illumina sequencing adapters were added to cDNA fragments of both sizes. After PCR amplification for DNA enrichment, AMPure XP Beads (Beckman) were used to clean up the target fragments of 200-300 bp. After library construction, a Qubit 2.0 fluorometric dsDNA HS Assay (Thermo Fisher Scientific) was used to quantify the concentrations of the resulting sequencing libraries, while the size distribution was analyzed by an Agilent BioAnalyzer 2100 (Agilent). Sequencing was performed using an Illumina system following Illumina-provided protocols for 2 × 150 paired-end sequencing in WuXi NextCODE.

### GSEA

Gene expression differences in biological pathways in MDL-811- or DMSO-treated HCT116 cells were analyzed by GSEA version 3.0 software 39 (http://www.broadinstitute.org/gsea/msigdb/index.jsp) under the default parameters with reference gene sets from the Molecular Signatures Database (MSigDB) C2 (CP: Kyoto Encyclopedia of Genes and Genomes [KEGG]) gene sets. The number of permutations was set at 1,000. Gene sets were considered significantly enriched if they satisfied the following cutoffs: nominal *P* < 0.05 and false discovery rate (FDR) < 0.25.

### RNA extraction and RT-qPCR

Total RNA was extracted with TRIzol reagent (Invitrogen) according to the manufacturer's instructions. For cDNA synthesis, 1 μg of total RNA was reverse transcribed using a FastKing RT Kit (Tiangen). RT-qPCRs were run in triplicate using SYBR Green master mix (Roche) in an ABI 7900 Thermal Cycler (Applied Biosystems) according to the manufacturer's instructions. RT-qPCRs were performed in triplicate, and the mean cycle threshold (Ct) values were calculated for expression analysis. The mRNA levels of target genes were calculated using the 2^-ΔΔCt^ method 40. The expression data were normalized to β-actin expression levels and are presented as the mean ± s.e.m. of three independent experiments. The RT-qPCR primer sequences are listed in Table S7.

### Transfection

HCT116 cells were transiently transfected with empty vector, pcDNA3.1-CYP24A1-Flag or pLVX-SIRT6-HA using Neofect™ DNA transfection reagent following the manufacturer's protocol. Cells were subjected to the following experiments 48 h after transfection.

### ChIP assay

ChIP assays were performed following the Chip-IT Express Kit (Active Motif) protocol. Briefly, HCT116 cells were treated with the indicated concentrations of MDL-811 for 48 h; the final DMSO concentration in the medium was 0.1%. Then, the cells were cross-linked for 10 min with 1% formaldehyde, and the reaction was stopped with 0.125 M glycine. Samples were sonicated into DNA fragments of 200-500 bp with 6 pulses at an amplitude of 25% using an EpiShear™ Cooled Sonication Platform (Active Motif). Each pulse consisted of sonication for 20 s followed by a rest on ice for 30 s. Then, 4% of soluble chromatin was retained as the input control. The remaining chromatin was incubated with the indicated antibodies and 25 μL of magnetic beads at 4 °C overnight with rotation. Information regarding the primary antibodies is provided in Table S8. Following reverse crosslinking at 65 °C for 8 h, bound DNA and input controls were purified using a MinElute PCR Purification Kit (Qiagen) following the manufacturer's instructions and were finally resuspended in 50 μL of ddH_2_O. ChIP-associated sequences were detected by RT-qPCR as described above. The ChIP-qPCR primers were designed based on the DNA sequence of each peak region of H3K9Ac, H3K18Ac or H3K56Ac from ChIP-seq data extracted from the Cistrome database (Figure S10, B to D). The ChIP-qPCR primer sequences for the indicated regions are provided in Table S9.

### Combination drug treatment

1,25(OH)_2_D_3_ was purchased from TargetMol. For cell proliferation assays, 1,25(OH)_2_D_3_ was dissolved in DMSO, stored at -20 °C and protected from light. The maximum final DMSO concentration used in all treatment conditions was 0.25%. CRC cell lines were exposed to MDL-811 at concentrations ranging from 5 to 10 μM plus 1,25(OH)_2_D_3_ at concentrations ranging from 1 to 10 μM. Then, cell proliferation assays were performed using the CCK-8 method. Combination effects were analyzed by the Chou-Talalay combination index (CI) method 41. The CI values and the fraction of cells affected values were generated using CompuSyn Version 1.0 software. CI values of < 1, = 1, and > 1 indicate synergism, additivity, and antagonism between the drugs, respectively. For combination treatment in the HCT116 CDX model, six-week-old male BALB/c nude mice (n = 8) were intraperitoneally treated every two days for 14 days with either vehicle (5% DMSO, 30% PEG-400, and 65% saline [pH 7.0-8.0]), 1,25(OH)_2_D_3_ alone (50 μg/kg), MDL-811 alone (15 mg/kg), or a combination of the two drugs.

### Statistical analysis

Significance was evaluated with ANOVA or two-tailed Student's t-tests unless otherwise indicated in the figure legends. *P* values less than 0.05 were considered significant (*, *P* < 0.05; **, *P* < 0.01; ***, *P* < 0.001). The graphs and error bars show the mean ± s.d. of independent biological experiments unless stated otherwise. All statistical analyses were performed using Microsoft Office Excel 2016 or GraphPad Prism V7.

Synthesis, NMR spectra, and the HPLC analysis data of MDL analogs are provided in Chemical Characterization of Supplemental Information.

## Results

### Identification of a potent and selective SIRT6 activator, MDL-811

Our previous efforts have focused on optimizing the two side phenyl rings of the screened hit scaffold, yielding MDL-800 and MDL-801 42. According to our co-crystal structure of MDL-801 and SIRT6 (PDB ID: 5Y2F), there is a sub-pocket surrounded by Phe82, Thr84, Thr85 and Phe86 around the C3 position of the central phenyl ring of MDL-801 (Figure S1), where MDL compounds may be modified to improve the characteristics of the SIRT6 activators. Guided by the structure, we synthesized a series of compounds at the C3 position by introducing moieties with different sizes and properties, leading to 12 MDL analogs (Table 1). Evaluated by the Fluor de Lys (FDL) assays with an acetylated peptide (RHKK-Ac-AMC), these compounds showed their half-maximal effective concentration (EC_50_) values from 5.7 to 43.6 μM. We tried to use a docking model to prompt the potential binding poses of these MDL analogs at the SIRT6 site (Figure S1 and Table 1). For example, the dimethylamine group at the C3 position could not form any positive interaction with the residues of the SIRT6 site (MDL-818, Figure S1A), but three MDL analogs with extended carbon chains of dimethylamine probably increased hydrophobic contacts with the residues in the site, such as the N-ethyl-N-methylbutan-1-amine group of MDL-813 with Phe82 and Thr85 (Figure S1B). Compared with the five-membered heterocycle (MDL-822, Figure S1C), we found that the size of a six-membered heterocycle (MDL-821) may make a more favorable adaption with the site (Figure S1D).

However, the conformation of the SIRT6 site is rather flexible based on the superimposed crystal structures and its high Wilson factors so that the docking simulation that generally treats a protein as a rigid structure 43-45 may be difficult to accurately predict the binding poses of MDL-811 and its analogs at the site, thus future co-crystals will be of great help to reveal the binding poses of the compounds on SIRT6.

As a drug-like six-membered heterocycle, morpholine and 3-methylmorpholine groups have been commonly used for desirable drug-like properties 46, 47. Thus, we used 3-methylmorpholine at the C3 position, leading to MDL-811. MDL-811 potently activated SIRT6 deacetylation (EC_50_ = 5.7 ± 0.8 μM), with two-fold greater activity than MDL-800 (EC_50_ = 12.3 ± 0.7 μM) (Figure S2A). Additionally, MDL-811 significantly enhanced bioavailability (F%, 92.96%) compared with MDL-800 (71.33%) (Table S1), which could improve the anti-tumor effect on CRC *in vivo*. In the docking model, the 3-methylmorpholine of MDL-811 could form a hydrogen bond with the backbone of Phe86 as well as participate in hydrophobic interactions with Phe82, Thr85, and Phe86 of SIRT6, which confers a more favorable binding mode than that of MDL-800 (Figure S1E). However, long-chain substituted morpholine could not further improve activity (MDL-815 and MDL-816). In addition, MDL analogs with a larger moiety, such as dipiperidine (MDL-814), at the C3 position were also explored to accommodate the hydrophobic pocket. Compared with MDL-811, the larger substituted dipiperidine of MDL-814 extended the pocket outward the pocket with fewer hydrophobic contacts, although the scaffold of the compound maintained the same conformation (Figure S1F), which could be the reason for its reduced activity. Collectively, MDL-811 could be promising for further *in vitro* and *in vivo* investigation.

**Figure 1:**
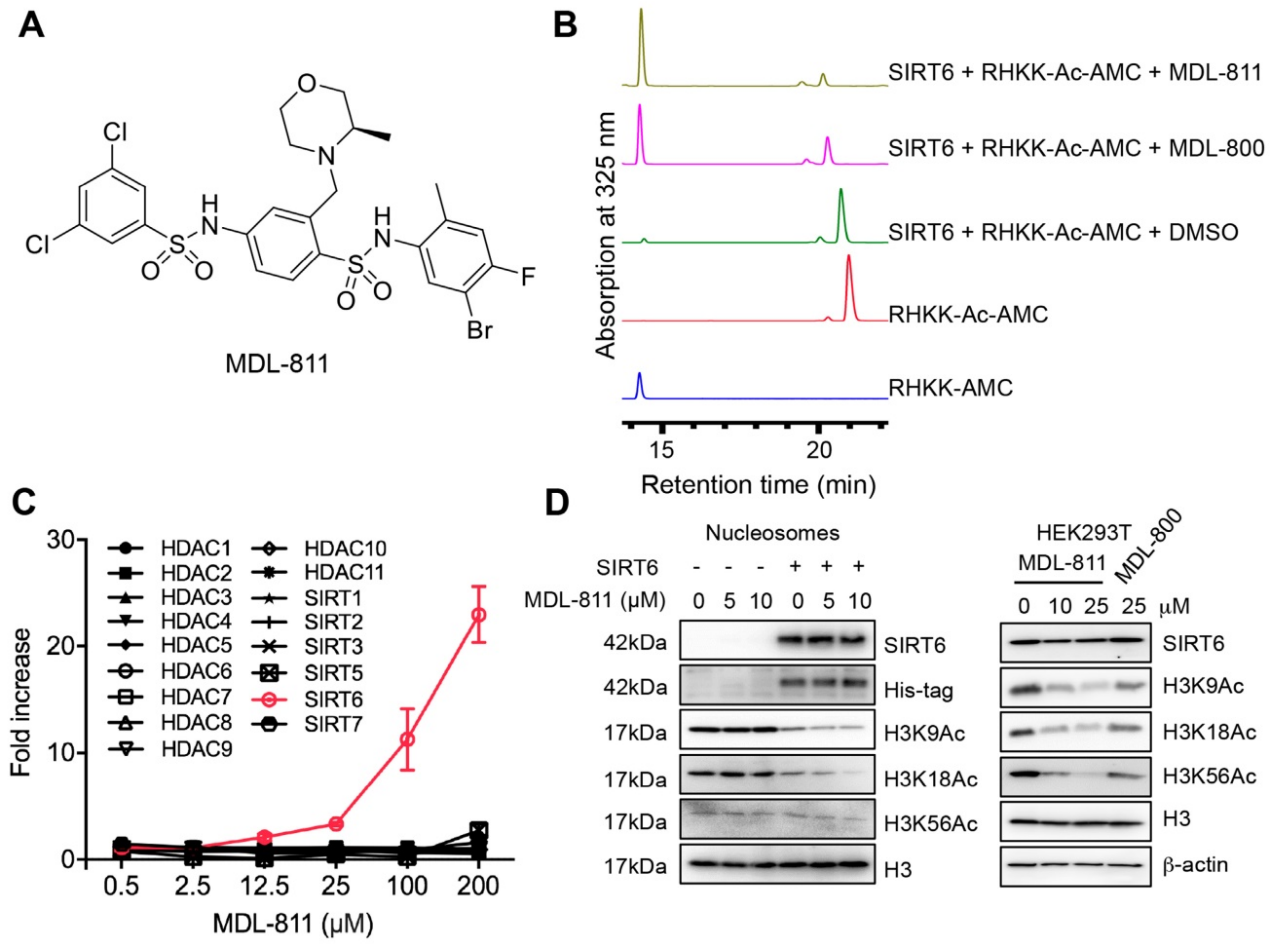
** Identification of MDL-811, a potent and selective allosteric SIRT6 activator.** (A) Chemical structure of MDL-811. (B) Effects of SIRT6 deacetylation on RHKK-Ac-AMC with DMSO, 25 μM MDL-811 or MDL-800, as determined by HPLC. The data are representative of the results of three independent experiments. (C) Target selectivity of MDL-811 among histone deacetylase enzymes. Assays were performed using the indicated concentrations of fluorogenic HDAC substrates for the indicated time. The data are presented as the mean ± s.d. of three independent experiments. (D) Representative western blots of the dose-dependent effects of MDL-811 on H3K9Ac, H3K18Ac, and H3K56Ac in nucleosomes and HEK293T cells. Histone H3 was the internal control, and β-actin was the loading control.

We further assessed the reproducibility of the activating effect using HPLC with RHKK-Ac-AMC as the substrate. The HPLC assay results confirmed that MDL-811 increased the catalytic efficiency for SIRT6 deacetylation to a higher level than MDL-800 (Figure 1B and Figure S2B). To determine whether MDL-811 might affect SIRT6 deacylase activity, we used a synthetic TNFα peptide containing a myristoyl group (EALPKK-Myr-AMC) to evaluate the effect of MDL-811 using an FDL assay as previously described 34, 48-50. The result showed no apparent effect of MDL-811 on SIRT6 deacylation (Figure S3).

The selectivity of MDL-811 was assessed across a panel of HDAC family members with deacetylase activity. MDL-811 potently enhanced the deacetylase activity of SIRT6 in a dose-dependent manner but showed little effect on other histone deacetylase enzymes at concentrations up to 100 μM (Figure 1C and Table S2), suggesting that MDL-811 selectively activated SIRT6 among the HDAC family members. More importantly, MDL-811 significantly enhanced the deacetylation of SIRT6 (H3K9Ac, H3K18Ac, and H3K56Ac) in natural nucleosomes and HEK293T cells in a dose-dependent manner (Figure 1D). Having confirmed the enhanced effect of MDL-811 on SIRT6 deacetylation, we next used this activator to evaluate the pharmacological activation of SIRT6 in CRC.

### MDL-811 exhibits broad antiproliferative effects on CRC cells

**Figure 2:**
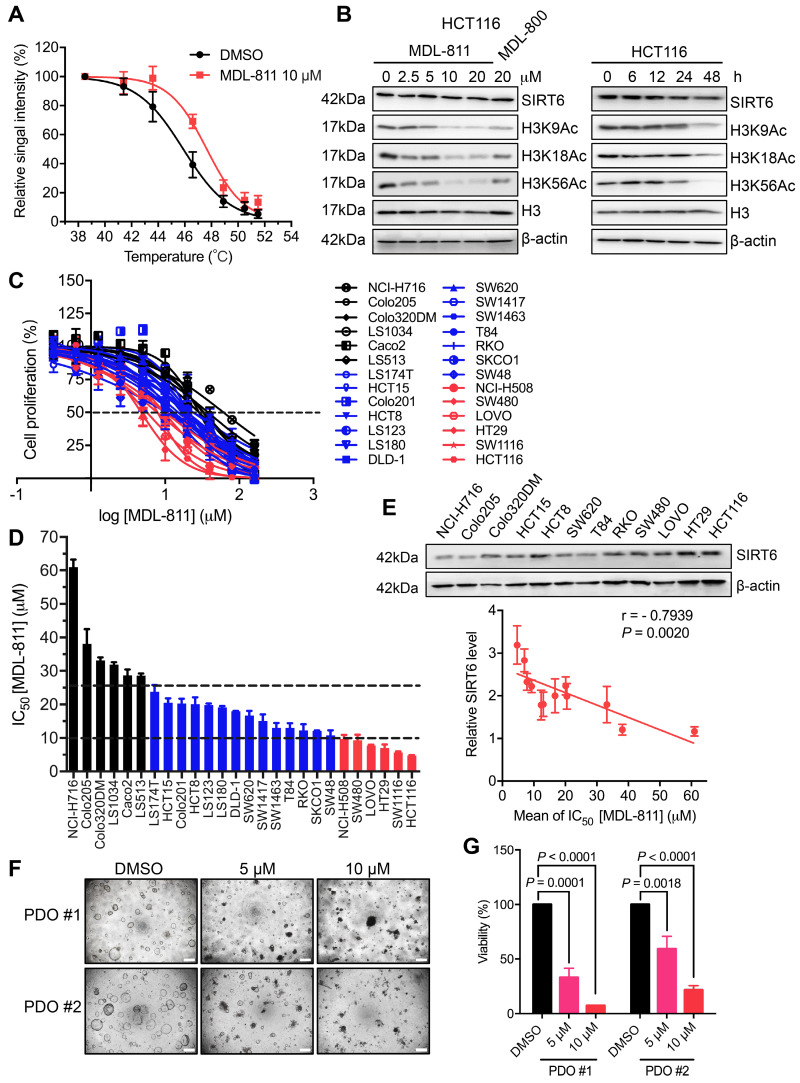
** MDL-811 exhibits broad antiproliferative effects on CRC**. (A) Graphical summary of the CETSA results in HCT116 cells treated with DMSO or 10 μM MDL-811 for 24 h. The data are plotted as the mean ± s.e.m. of four independent experiments. (B) Representative western blots of SIRT6 and H3K9Ac, H3K18Ac, and H3K56Ac levels in HCT116 cells treated with the indicated concentrations of MDL-811 or 20 μM MDL-800 for 48 h and treated with 10 μM MDL-811 for the indicated times. Histone H3, internal control, and β-actin, loading control. (C) Dose response of the proliferation of various CRC cell lines exposed to MDL-811 for 48 h was normalized to the proliferation of the corresponding DMSO-treated controls. Cell proliferation was determined by a CCK-8 assay. Black lines, CRC cell lines with IC_50_ values greater than 25 μM; blue lines, between 10 and 25 μM; red lines, less than 10 μM. The points indicate the mean ± s.d. of two or three independent experiments. (D) The IC_50_ values of MDL-811 in various CRC cell lines following 48 h of treatment. The data are presented as the mean ± s.d. of two or three independent experiments. (E) Representative western blots of SIRT6 expression in twelve CRC cell lines (upper panel). Correlation analysis of the SIRT6 percentage relative to that of β-actin for twelve CRC cell lines with IC_50_ values of MDL-811 at 48 h (lower panel). Each IC_50_ value is the average value of two or three independent experiments. Quantification of the SIRT6 level was calculated by ImageJ V4. The SIRT6 level in each CRC cell line is presented as the mean ± s.e.m. of three independent experiments. The SIRT6 level and IC_50_ value of MDL-811 were subjected to Pearson correlation analysis (r = -0.7939, *P* = 0.0020, n = 12). (F) Representative photomicrographs of two CRC PDOs (PDO #1-2) treated with DMSO or with 5 or 10 μM MDL-811 (40× magnification). Scale bars, 200 μm. (G) Two CRC PDOs (PDO #1-2) were treated with the indicated concentrations of MDL-811 for 72 h, and cell viability was measured by CellTiter-Glo. Relative viability was normalized to that of the DMSO controls. Error bar, s.d. of two replicates; *P* values were determined by two-way ANOVA.

To pharmacologically evaluate the role of SIRT6 in CRC, we first tested the target engagement of MDL-811 in cells using a cellular thermal shift assay (CETSA) 37. HCT116 cells were incubated with 10 μM MDL-811 and then heated at the indicated temperatures to denature proteins (Figure 2A). MDL-811 increased the thermal stability of SIRT6, with an increase in the melting temperature from 45.8 °C to 47.6 °C (ΔT_m_ = 1.8 ± 0.2 °C) (Figure S4A and Table S3), indicating that MDL-811 can bind and stabilize its target, SIRT6, in CRC cells. Because SIRT6 catalyzes the deacetylation of H3K9Ac, H3K18Ac, and H3K56Ac, a cell-permeable activator of SIRT6 would be expected to decrease the levels of H3K9Ac, H3K18Ac, and H3K56Ac. We assessed the acetylation levels of these H3 markers after MDL-811 treatment by western blotting in a panel of human CRC cell lines and found that MDL-811 effectively enhanced the dose-dependent deacetylation of H3K9Ac, H3K18Ac, and H3K56Ac in almost every CRC cell line (Figure 2B, Figure S4, B and C). Notably, MDL-811-induced deacetylation of H3K9Ac, H3K18Ac, and H3K56Ac in HCT116, HT29, and SW480 cells was significantly stronger than that in other cell lines (Figure 2B and Figure S4B), consistent with the higher protein levels of SIRT6 in these three cell lines (Figure 2E) and supporting a positive association between the action of MDL-811 and the protein level of SIRT6 in CRC cells.

Recent findings have demonstrated that SIRT6 can suppress tumorigenesis by reducing the levels of H3K9Ac and H3K56Ac 10, 11, 23, 30. To quantitatively evaluate whether this association has a pharmacological effect on tumorigenesis, we assessed the anticancer effects of MDL-811 in the 26 CRC cell lines. As expected, MDL-811 extensively reduced CRC cell proliferation in a dose-dependent manner (Figure 2C), with half-maximum inhibitory concentration (IC_50_) values ranging from 4.7 to 61.0 μM (Figure 2D and Table S4). Importantly, the proliferation of more than 75% (20 of 26) of the CRC cell lines was substantially inhibited by MDL-811 (IC_50_ < 25 μM). Further analysis revealed that the IC_50_ values of MDL-811 in the CRC cell lines negatively correlated with not only the protein levels of SIRT6 (Pearson correlation, r = -0.7939, *P* = 0.0020, n = 12) (Figure 2E) but also the deacetylation of H3K9Ac (r = -0.4734, *P* = 0.0146, n = 26) and H3K56Ac (r = -0.4631, *P* = 0.0172, n = 26) after treatment with MDL-811 (Figure S4D). Among the CRC cell lines, the higher the SIRT6 protein level in the CRC cell line, the greater was the effect of MDL-811 treatment on both the activation of SIRT6-mediated deacetylation and the anti-CRC effect. Collectively, these results reveal the common role of SIRT6 in the proliferation of various CRC cell lines and indicate that the pharmacological effect of MDL-811 is due to reduced histone H3 acetylation through the activation of SIRT6 deacetylase activity.

To investigate the function of MDL-811 in CRC cells, we assessed cell viability in the presence of 10 μM MDL-811 using a live-dead double staining assay. MDL-811 did not cause observable cell death but led to a significant decrease in the number of CRC cells at the pharmacological concentration (Figure S5A). The results of lactate dehydrogenase (LDH) assays confirmed the minimal cytotoxic effect of MDL-811 at these concentrations (Figure S5B), implying that MDL-811 could induce an anti-tumor effect by suppressing CRC cell proliferation instead of via cytotoxicity. Moreover, we evaluated the antiproliferative and cytotoxic effects of MDL-811 in the non-cancerous colon cell line FHC using CCK8 and LDH assays. Our results showed that the IC_50_ of FHC cells was approximately 5 times higher than that of CRC cell line HCT116 in CCK8 assays (Figure S6). Consistent with the relatively low cytotoxic effect of MDL-811 in CRC cell lines, we also observed a low cytotoxic effect of MDL-811 in FHC cells in LDH assays (Figure S5B). Moreover, MDL-811 induced marked G_0_/G_1_ cell cycle arrest in CRC cells (Figure S7A and Table S5), consistent with recent findings that SIRT6 activation suppresses cell cycle progression in tumor cells 21, 23, 27, 51.

Patient-derived organoids (PDOs) more closely recapitulate the genetic and pathological properties of the original tumor than do adherent monolayer cultures and allow for the prediction of patient responses to drug treatment with improved accuracy 38. To evaluate the clinical potential of MDL-811, we measured the efficacy of MDL-811 in two organoids derived from two independent CRC tumors (PDO #1-2). The relative viability of the organoids was determined using a CellTiter-Glo assay. Consistent with the results of our CRC cell line-based assays, MDL-811 significantly repressed the growth of these two CRC PDOs in a dose-dependent manner (Figure 2, F and G), suggesting the potential clinical efficacy of MDL-811. Taken together, our data demonstrate that MDL-811 pharmacologically exerts broad antiproliferative effects on CRC cells.

### MDL-811 elicits pharmacological inhibition on CRC *in vivo*

**Figure 3:**
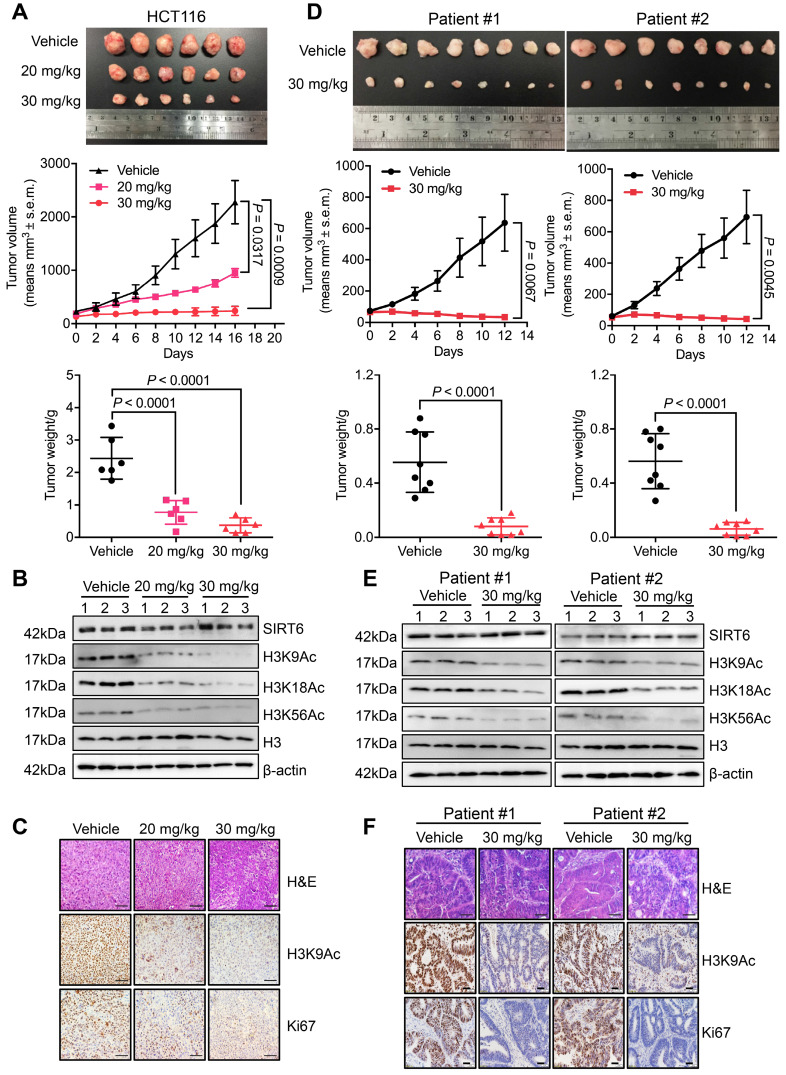
** MDL-811 exhibits robust anti-tumor efficacy in CRC CDX and PDX models.** (A) Representative image of HCT116 xenograft tumors dissected from nude mice in different groups (upper panel). Volumetric measurements of HCT116 xenograft tumors treated intraperitoneally with vehicle or with 20 or 30 mg/kg MDL-811 every other day for 16 days (middle panel). The tumor volumes are plotted as the mean ± s.e.m. of n = 6 mice per group. Tumor weights in different groups of mice (bottom panel) are shown. The data are presented as the mean ± s.d. of n = 6 mice per group. *P* values were determined by one-way ANOVA. (B) Representative western blots of SIRT6, H3K9Ac, H3K18Ac, and H3K56Ac in three representative xenograft tumors from each group. Histone H3, internal control, and β-actin, loading control. (C) Representative tissue sections from HCT116 xenograft tumors with H&E, H3K9Ac, and Ki67 staining after treatment with vehicle or MDL-811. Representative images (40× magnification) are shown. Scale bars, 200 μm. (D) Representative images of PDX tumors from CRC patients #1 and #2 dissected from mice of different groups (upper panel). Volumetric measurements of PDX tumors from CRC patients #1 and #2 treated intraperitoneally with vehicle or 30 mg/kg MDL-811 every other day for 12 days (middle panel). The tumor volumes are plotted as the mean ± s.e.m. of n = 8 mice per group. Tumor weights in different groups of mice (bottom panel) are shown. The data are presented as the mean ± s.d., and *P* values were determined by a two-tailed Student's unpaired t-test (n = 8). (E) Representative western blots of SIRT6, H3K9Ac, H3K18Ac, and H3K56Ac in three representative PDX tumors from CRC patients #1 and #2 in each group of mice. (F) Representative tissue sections from PDX tumors of CRC patients #1 and #2 with H&E, H3K9Ac, and Ki67 staining after treatment with vehicle or 30 mg/kg MDL-811. Representative images are shown (40× magnification). Scale bars of H&E staining, 200 μm; Scale bars of H3K9Ac and Ki67 staining, 20 μm.

To evaluate the pharmacological effect of MDL-811 *in vivo*, we generated both CDX and PDX models. In the HCT116 CDX model, MDL-811 treatment strongly suppressed tumor growth in a dose-dependent manner (Figure 3A), indicating robust anti-CRC activity of MDL-811 *in vivo*. Western blot analyses of xenograft tissues showed that SIRT6-dependent deacetylation of histone H3 marks was significantly increased in xenografts treated with MDL-811 (Figure 3B). Moreover, immunohistochemical (IHC) staining provided additional evidence that CRC tumor regression was associated with decreased proliferation (Ki67) and reduced H3K9Ac levels in xenografts after treatment with MDL-811 (Figure 3C). To further confirm the therapeutic effect of MDL-811, we established two PDX models from tumors resected from two individual CRC patients. MDL-811 treatment led to strong tumor regression in both PDX models (Figure 3D), with decreased Ki67 staining and reduced SIRT6-specific histone H3 acetylation marks (Figure 3, E and F), consistent with the pharmacological effect of MDL-811 in the CDX model. In particular, we did not observe any significant weight loss or other abnormal behavioral signs in the mice treated with MDL-811, indicating that MDL-811 has little toxicity and is well-tolerated at the efficacy dosage *in vivo* (Figure S8). These xenograft models clearly reveal the therapeutic efficacy of MDL-811-induced SIRT6 activation in CRC *in vivo*.

**Figure 4:**
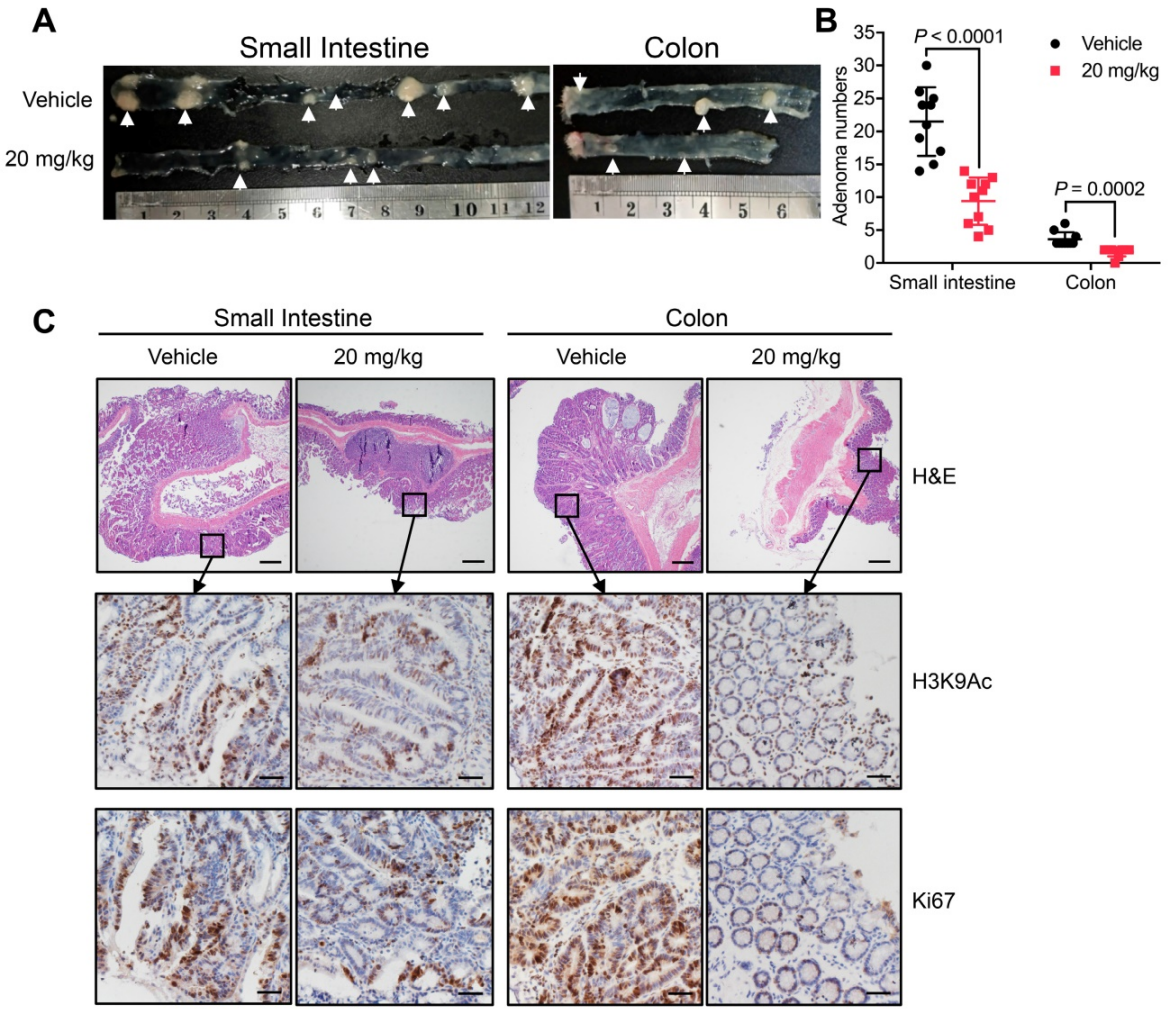
** MDL-811 inhibits tumorigenesis in the APC^min/+^ model of spontaneous CRC.** (A) Representative image of intestine and colon sections from APC^min/+^ mice. Mice received an intraperitoneal injection of vehicle alone or 20 mg/kg MDL-811 every two days for 12 weeks. The white arrows indicate the presence of polyps. (B) Adenoma numbers in the small intestines and colons of APC^min/+^ mice. The error bars indicate the mean ± s.d., and *P* values were determined by a two-tailed unpaired Student's t-test (n = 10 mice per group). (C) H&E staining showing the morphology of the small intestine or colon from the indicated mice (4× magnification). Scale bars, 200 μm. The lower four panels of each figure are zoomed-in images of the areas enclosed in the rectangles in the top two panels. The expression of H3K9Ac or Ki67 in the tumor tissues was analyzed by IHC staining. Representative images are shown (40× magnification). Scale bars, 20 μm.

In addition to xenograft models, models of spontaneous CRC can provide further evidence supporting the therapeutic potential of MDL-811. Thus, APC^min/+^ mice were treated with either vehicle or 20 mg/kg MDL-811 by intraperitoneal injection every two days for 12 weeks. MDL-811 significantly reduced the number and size of adenomas in both the small intestines and colons of APC^min/+^ mice (Figure 4, A and B). Moreover, moderate decreases in the levels of Ki67 and H3K9Ac were observed after MDL-811 treatment relative to these levels after treatment with vehicle control, as evaluated by IHC staining (Figure 4C). Our pharmacological results agree well with recent reports stating that conditional intestinal ablation of SIRT6 increases the size and number and promotes the aggressiveness of adenomas in APC^min/+^ CRC mice 10. Collectively, our results demonstrate that MDL-811 effectively elicits pharmacological inhibition of CRC *in vivo*.

### MDL-811 suppresses the transcription of CYP24A1 in CRC

**Figure 5:**
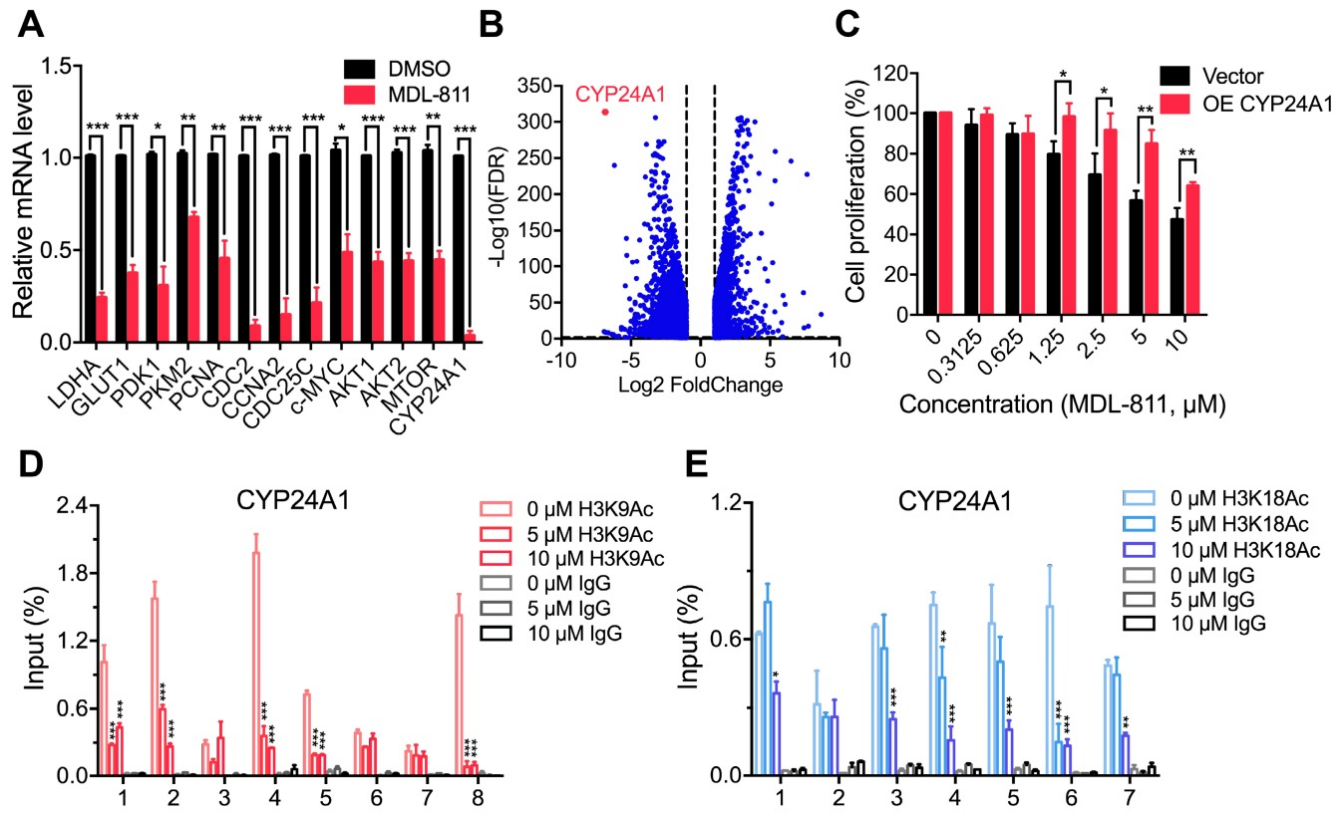
** MDL-811 suppresses CYP24A1 gene transcription in CRC.** (A) RT-qPCR analyses showing the mRNA levels of candidate genes in HCT116 cells treated with DMSO or 10 µM MDL-811 for 48 h. The data are normalized to the β-actin levels and presented as the mean ± s.e.m. of three independent experiments. *P* values were determined by a two-tailed unpaired Student's t-test (*, *P* < 0.05; **, *P* < 0.01; ***, *P* < 0.001). (B) Volcano plot showing differentially expressed genes in HCT116 cells after treatment with DMSO or 10 μM MDL-811 for 48 h (absolute log2 fold change > 1, FDR q < 0.05). (C) Effect of CYP24A1 overexpression on the suppressive effect of MDL-811 in HCT116 cells. HCT116 cells were transiently transfected with vector or pcDNA3.1-CYP24A1-Flag for 48 h before being treated with MDL-811 for 48 h. The data are presented as the mean ± s.d. of three independent experiments. *P* values were determined by two-tailed unpaired Student's t-test (*, *P* < 0.05; **, *P* < 0.01; ***, *P* < 0.001). (D and E) ChIP assays using anti-H3K9Ac (D) or anti-H3K18Ac (E) antibody to detect H3K9Ac or H3K18Ac occupancy in the indicated regions of CYP24A1 in HCT116 cells treated with DMSO or with 5 or 10 μM MDL-811 for 48 h. The data are presented as the mean values of the percentage of input ± s.e.m. from one of three independent experiments with technical triplicates. *P* values were determined by two-way ANOVA (*, *P* < 0.05; **, *P* < 0.01; ***, *P* < 0.001).

To explore the mechanism of MDL-811 in CRC, we performed RNA sequencing analysis to investigate the gene expression profiles of MDL-811- and DMSO-treated HCT116 cells. Many studies have shown that SIRT6 can be recruited to the chromatin to repress gene transcription and affect genomic stability through histone deacetylation at specific genomic regions 10, 11, 23, 25, 26, 28-30. Gene Set Enrichment Analysis (GSEA) showed that MDL-811 induced significant changes in the expression of genes associated with the downregulation of DNA replication and cell cycle activity (Figure S7B), consistent with the effect of MDL-811 on cell cycle arrest in CRC (Figure S7A and Table S5). Regarding the differentially expressed genes after treatment with MDL-811, real-time quantitative PCR (RT-qPCR) assays revealed that the transcription levels of the genes known to exhibit SIRT6-specific repression were considerably decreased (Figure 5A). These genes included LDHA, GLUT1, PDK1, and PKM2 (involved in Hif1α-mediated glycolytic metabolism) 28, 52; PCNA, CDC2, CCNA2, and CDC25C (c-MYC target genes and cell cycle checkpoint regulators) 21; and AKT1, AKT2, and MTOR (IGF signaling-related genes) 29. These decreases were consistent with the decreases in the mRNA levels of these genes in HCT116 cells by SIRT6 overexpression (Figure S9, A and B).

Notably, CYP24A1 (or 1,25-dihydroxyvitamin D_3_ 24-hydroxylase) was one of the most strongly suppressed genes after MDL-811 treatment (Figure 5B). CYP24A1 is a member of the cytochrome P450 enzyme family involved in drug metabolism and the synthesis of cholesterol, steroids, and other lipids 53.

The RT-qPCR results confirmed that MDL-811 markedly decreased CYP24A1 mRNA expression levels (Figure 5A), consistent with the change in the mRNA expression level of CYP24A1 in HCT116 with SIRT6 overexpression (Figure S9C), implying that CYP24A1 could be a downstream gene regulated by SIRT6. To investigate whether CYP24A1 is a direct downstream target of SIRT6, we performed a ChIP-qPCR assay to detect the binding site of SIRT6 at the CYP24A1 gene locus in HCT116 cells. Our ChIP-qPCR results showed strong signals corresponding to histone H3 acetylation marks in the CYP24A1 genomic regions compared with those in the IgG control, and both MDL-811 treatment and SIRT6 overexpression resulted in the deacetylation of H3K9Ac and H3K18Ac at the CYP24A1 gene locus (Figure 5, D and E, Figure S9, D and E) but had a minimal effect on the level of H3K56Ac (Figure S10A). This pattern was similar to the results of analysis of ChIP-seq data from the Cistrome database 54, showing that the CYP24A1 gene exhibits strong H3K9Ac and H3K18Ac signals and a relatively weak H3K56Ac signal in the region surrounding the transcription start site (Figure S10, B to D). Then, we identified the direct binding of SIRT6 at the CYP24A1 gene locus in cells with not only endogenous SIRT6 but also overexpressed HA-SIRT6 (Figure S9F), suggesting that CYP24A1 is a newly identified direct downstream target gene of SIRT6 in CRC cells and that MDL-811 inhibits CYP24A1 gene transcription by decreasing the levels of H3K9Ac and H3K18Ac. Finally, we evaluated whether the anti-tumor efficacy of MDL-811 in CRC is mediated by CYP24A1 downregulation. We assessed the proliferation of HCT116 cells with CYP24A1 overexpression (Figure S11) and observed that compared with the control groups, CYP24A1-overexpressing cells were resistant to MDL-811 treatment (Figure 5C), indicating that CYP24A1 downregulation could mediate the decrease in CRC cell proliferation induced by MDL-811.

### MDL-811 enhances the anti-tumor activity of vitamin D_3_ in CRC

**Figure 6:**
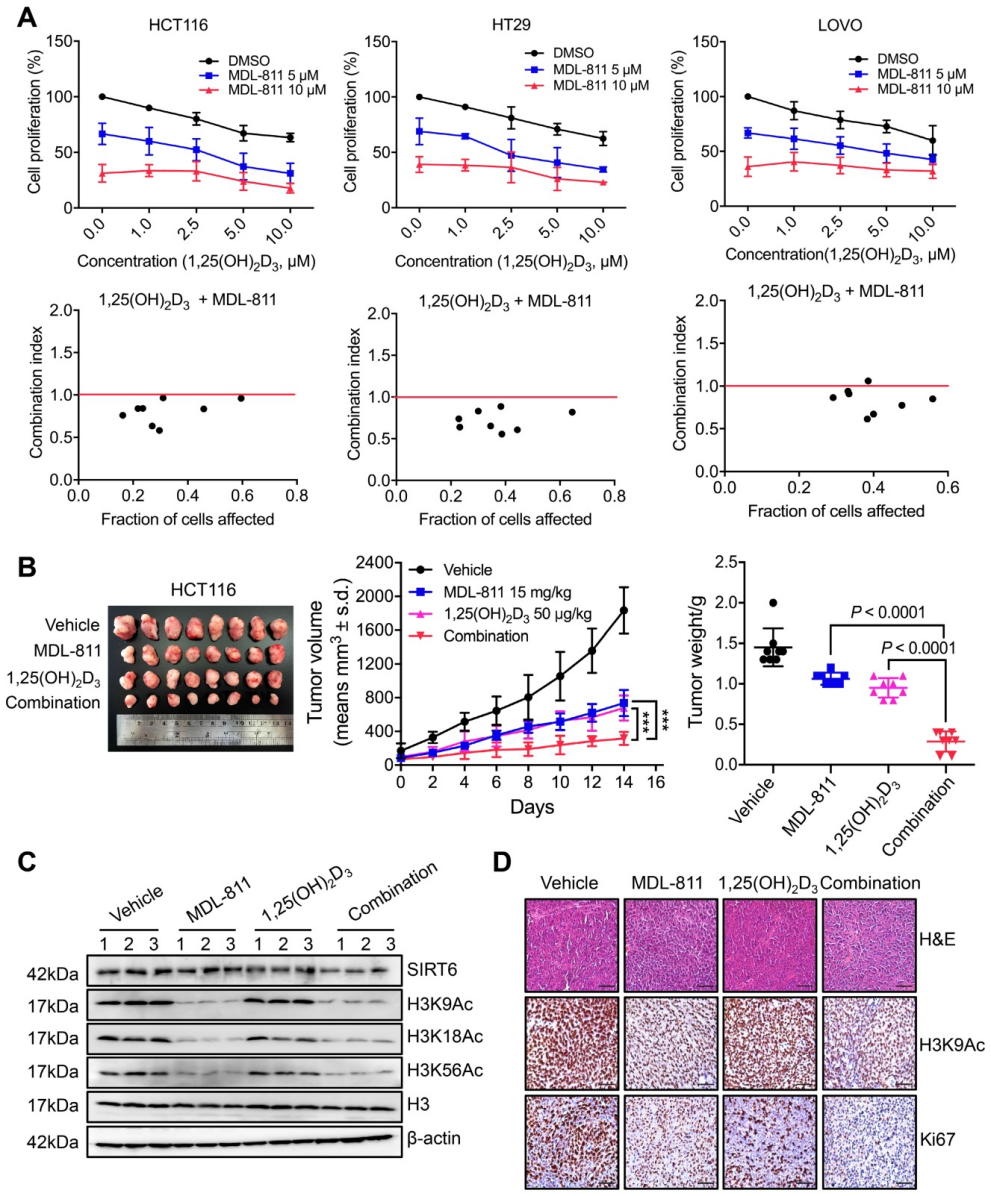
** MDL-811 enhances the anti-tumor activity of vitamin D_3_ in CRC**. (A) Cell proliferation was assessed following 48 h of exposure to the indicated concentrations of MDL-811, 1,25(OH)_2_D_3_ or the combination in HCT116, HT29, and LOVO cells. CI values for the various combinations were calculated using CompuSyn Version 1.0 software. Synergism, additivity, and antagonism are defined as CI < 1, = 1, and > 1, respectively. The data are presented as the mean ± s.d. of three independent experiments. (B) Representative image of HCT116 xenograft tumors dissected from nude mice in different groups (left). Tumor growth curves of HCT116 CDXs treated with intraperitoneal administration of vehicle, 15 mg/kg MDL-811, 50 μg/kg 1,25(OH)_2_D_3_ or the combination every other day for 14 days (middle). The data are presented as the mean ± s.d. of n = 8 mice per group (two-way ANOVA). Tumor weights in different groups of mice. The data are presented as the mean ± s.d. of n = 8 mice per group. *P* values were determined by one-way ANOVA. (C) Representative western blots of SIRT6, H3K9Ac, H3K18Ac, and H3K56Ac in three representative HCT116 xenograft tumors from each group. Histone H3, internal control, and β-actin, loading control. (D) Representative tissue sections from HCT116 xenograft tumors with H&E, H3K9Ac, and Ki67 staining after treatment with vehicle, 15 mg/kg MDL-811, 50 μg/kg 1,25(OH)_2_D_3_ or the combination. Representative images (40× magnification) are shown. Scale bars, 200 μm.

VD_3_, especially its most potent metabolite, 1,25-dihydroxyvitamin D_3_ (1,25(OH)_2_D_3_), shows anti-tumor efficacy in CRC through the inhibition of cell proliferation and induction of apoptosis by binding to the vitamin D receptor (VDR), and VD_3_/1,25(OH)_2_D_3_ deficiency is linked to a high incidence of neoplasia 55-57. CYP24A1 is both a crucial inactivating enzyme of VD_3_/1,25(OH)_2_D_3_ and a downstream transcriptional target of VDR; thus, transcriptional activation of CYP24A1 by 1,25(OH)_2_D_3_ can attenuate the anti-tumor effect of VD_3_ therapy 53, 58. Indeed, previous studies have shown that CYP24A1 inhibition markedly increases the anti-tumor activity of 1,25(OH)_2_D_3_ in CRC cells 59, 60, suggesting that inhibition of CYP24A1 transcription by MDL-811 may enhance the efficacy of 1,25(OH)_2_D_3_ for CRC treatment. Based on this hypothesis, we assessed the mRNA levels of CYP24A1 in CRC cells treated with 1,25(OH)_2_D_3_ and MDL-811. As expected, 1,25(OH)_2_D_3_ dramatically induced CYP24A1 transcription in HCT116 and HT29 cells, while this enhanced transcription of CYP24A1 in response to 1,25(OH)_2_D_3_ was effectively abolished by cotreatment with MDL-811 (Figure S12), indicating that MDL-811 disrupts the VD_3_-VDR-CYP24A1 negative feedback loop. In addition, combination treatment with 1,25(OH)_2_D_3_ and MDL-811 exhibited significantly higher efficacy in inhibiting proliferation than the single agent in three CRC cell lines (Figure 6A). Moreover, CI analyses via the Chou-Talalay method 41 indicated that MDL-811 synergistically enhanced the anti-tumor activity of 1,25(OH)_2_D_3_, with CI values of < 1 in three CRC cell lines (Figure 6A). Next, we examined the synergistic effect of MDL-811 and VD_3_ in the CDX model. Compared with vehicle or single-drug treatment, cotreatment with MDL-811 and 1,25(OH)_2_D_3_ dramatically enhanced tumor growth regression, consistent with the reduction in Ki67 staining (Figure 6, B and D). The results of both western blotting and IHC staining also confirmed the decrease in histone H3 acetylation marks mediated by SIRT6 after MDL-811 treatment (Figure 6, C and D). Collectively, these data support a rationale for the use of SIRT6 activators in combination with VD_3_ for CRC therapy.

## Discussion

Surgery, radiation, and chemotherapy are standard treatments for CRC, but overall clinical outcomes remain unsatisfactory 2, 3. Thus, the need to discover innovative drugs against refractory CRC is urgent. Epigenetic alteration is a hallmark of cancer, and epigenetic enzymes dynamically and reversibly control protein translational modifications rather than globally affecting the DNA sequence 61. Increasing numbers of drugs targeting epigenetic enzymes have recently been developed and introduced into the anticancer drug market, such as inhibitors of DNA methyltransferase (DNMTis), HDACs (HDACis), and bromodomain and extraterminal domain proteins (BETis). These epigenetic drugs exhibit improved clinical efficacy and tolerance in patients 61. SIRT6 is a crucial epigenetic regulator of gatekeeping physiological and pathological functions, including tumorigenesis, via SIRT6-mediated histone H3 deacetylation 10, 11, 23, 30. Therefore, SIRT6 as a new epigenetic target is currently attracting increasing interest to solve the current dilemma in cancer treatment. To date, only a few compounds have been identified to regulate the deacetylase activity of SIRT6. Several SIRT6 inhibitors have been reported 62-67, and they exhibit potential for the treatment of certain cancers in which elevated SIRT6 expression may lead to malignancy 16-20. For example, quinazolinedione derivatives and polyphenols 62-64, as well as a series of peptides and pseudopeptides 66, 67, were discovered to inhibit SIRT6 deacetylation but did not exhibit selectivity toward SIRT6.

Morpholine and 3-methylmorpholine groups have been widely found in various clinical drugs for favorable drug-like properties, such as facile synthetic routes, advantageous solubility, potency, and pharmacokinetic profiles 46, 47. For example, Gefitinib is a selective epidermal growth factor receptor tyrosine kinase inhibitor (EGFR-TKI) for the treatment of cancer that contains the morpholine moiety for prolonged half-life, improved bioavailability, and lack of toxic metabolites 68, 69. AZD2014, a potent and specific mTOR inhibitor with anti-tumor efficacy, incorporates the 3-methylmorpholine to increase solubility, oral bioavailability, and metabolic stability 70, 71. Therefore, we used 3-methylmorpholine at the C3 position to yield MDL-811. As expected, MDL-811 potently and selectively activated SIRT6 deacetylation, and significantly increased bioavailability, which contributes to the robust anti-CRC effect *in vivo*. We have previously demonstrated the specificity of the SIRT6 allosteric site 34. Sequence alignment of SIRT1-7 revealed the high evolutionary diversity of the SIRT6 allosteric site; overlapping structures between SIRT6 and the other four sirtuins (SIRT1, SIRT2, SIRT3, and SIRT5) of known structures showed that a single helix in the structures of the other sirtuins traverses the same location as the site of SIRT6 (Figure S13), precluding the presence of a similar site in the other sirtuins for MDL-811 binding. To further explain the selectivity of MDL-811 over other sirtuins, we docked MDL-811 into the site of SIRT6. The result showed that MDL-811 is too large to be accommodated in the area of SIRT1-3 and 5 (Figure S1E and S13), indicating the structural distinction of the SIRT6 site among the sirtuin family members that allows for the high specificity of MDL-811.

Though the role of SIRT6 in cancers is cell-context dependent, and SIRT6 plays an oncogenic role in some cancers, such as skin cancer, squamous cell carcinoma, prostate cancer, and acute myeloid leukemia 16-20, SIRT6 acts as a tumor suppressor in multiple cancers including CRC and could be a potential epigenetic drug target for CRC treatment. Accumulating clinical studies have shown that reduced SIRT6 expression correlates with tumor progression and poor prognosis in CRC and promotes CRC proliferation 6, 10, 21, 22. Furthermore, SIRT6 overexpression can inhibit the growth of CRC stem cells 23, suggesting that SIRT6 activation has therapeutic potential in CRC. Consistent with the clinical analysis results, the SIRT6 activator MDL-811 demonstrated the antiproliferative effect of SIRT6 activation on CRC, from cell line-based assays to three powerful preclinical models (PDO, PDX, and APC^min/+^ spontaneous CRC models). These results are the first to provide pharmacological evidence for targeting SIRT6 in CRC and for a promising lead compound as a SIRT6 activator for future preclinical and clinical studies of CRC treatment.

Here, we found that CYP24A1 is a direct downstream target gene of SIRT6 through histone deacetylation in CRC. Previous epidemiological and clinical reports have noted that CYP24A1 expression is aberrantly elevated in CRC and is closely related to tumor progression and poor clinical outcomes 72, 73. In addition, CYP24A1 overexpression in CRC cells and mouse xenografts grants a proliferative advantage and aggressiveness to tumors 74. However, the regulation of CYP24A1 in CRC remains unclear due to the lack of effective screening tools. In this study, the discovery of MDL-811 reveals that SIRT6 is an upstream regulator of CYP24A1 in CRC through regulating the transcriptional response of CYP24A1. Moreover, oncogenic CYP24A1 can promote tumor cell cycle progression by regulating genes involved in G_0_/G_1_ phase, such as cyclin A, c-Myc, p21, p27, and GADD45A 55, which is closely associated with the cell cycle arrest resulting from SIRT6 activation in CRC; these observations suggest that CYP24A1 could be a downstream target gene for SIRT6 responsible for cell cycle regulation. However, considering the complicated set of genes targeted for SIRT6-mediated histone deacetylation in CRC, more effort should be further directed in the future to understanding the therapeutic mechanism between CYP24A1 and other known genes from SIRT6 activation.

Combination therapy is a promising approach to improve drug efficacy and overcome drug resistance 75. Many studies have indicated that epigenetic aberrations are more likely than other modifications to cause tumor immune escape and drug resistance, implying that combining epigenetic drugs with conventional therapies increases the susceptibility of tumors to treatment 61; examples include the combination of decitabine (a DNMTi) and carboplatin and the combination of vorinostat (an HDACi) and oxaliplatin 76. Extensive clinical results indicate that VD_3_ therapy is linked to a decreased mortality risk and an improved survival rate in CRC patients 55-57, and its active metabolic product 1,25(OH)_2_D_3_ can exert anti-tumor effects on refractory CRC through the induction of G_0_/G_1_ cell cycle arrest and apoptosis as well as the inhibition of angiogenesis 55. In CRC, CYP24A1 not only can be upregulated by 1,25(OH)_2_D_3_ treatment but also inactivates 1,25(OH)_2_D_3_, leading to an attenuated anti-tumor effect of VD_3_ 53, 58. These findings indicate that inhibition of CYP24A1 can disrupt the VD_3_-CYP24A1 negative feedback loop and further enhance the efficacy of VD_3_ therapy. Here, we showed that MDL-811 may induce the transcriptional repression of CYP24A1 through the activation of SIRT6 deacetylation; thus, we rationally established a combination therapy approach to increase the sensitivity of CRC to VD_3_ therapy by the addition of the SIRT6 activator MDL-811. This approach can serve as a promising next-generation translational medicine platform for CRC therapy.

## Conclusion

Our study discovered a potent and selective activator of SIRT6, MDL-811, which exhibited strong antiproliferative efficacy against CRC in multiple cell-based assays (CRC cell lines and PDOs) and *in vivo* models (CDX, PDX, and APC^min/+^ models). Using MDL-811, we revealed CYP24A1 as a new downstream target of SIRT6 through histone H3 deacetylation. Based on this finding, we designed a combination strategy with MDL-811 to synergistically enhance the anti-CRC effect of vitamin D_3_. Collectively, we provide the first pharmacological evidence for supporting the future preclinical and clinical application of a promising lead compound as a SIRT6 activator, alone or combined with vitamin D_3_ for CRC treatment.

## Supplementary Material

Supplementary figures, data, and tables.

## Figures and Tables

**Table 1:**
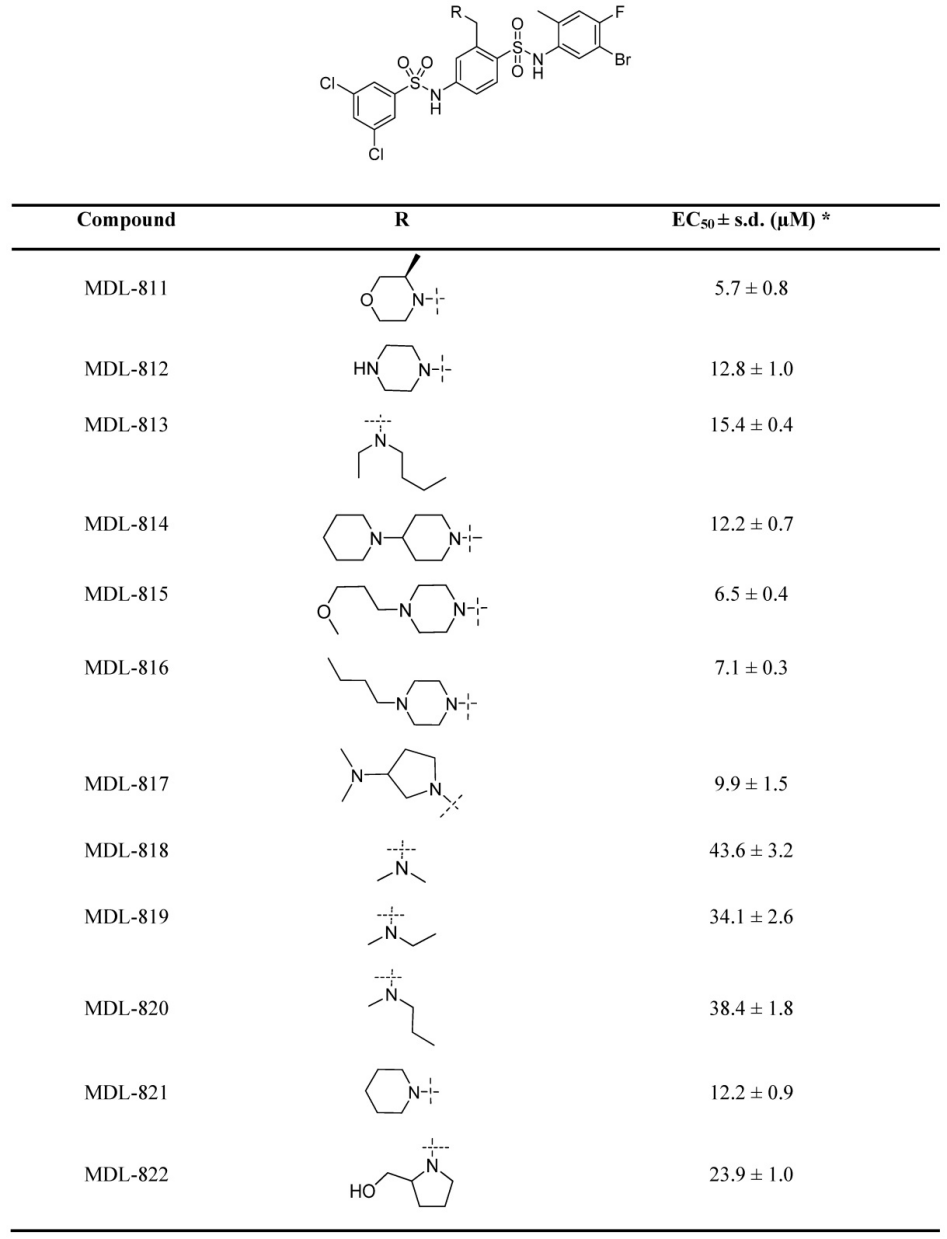
Chemical structures of MDL analogs and activity.

^*^ EC_50_ values were determined by FDL assays. The data are shown as mean ± s.d. from three independent experiments.

## Abbreviations

ANOVA: Analysis of variance
APC^min/+^: Adenomatous polyposis coli multiple intestinal neoplasia mouse
BETis: Bromodomain and extraterminal domain proteins inhibitors
BSA: Bovine serum albumin
CCK-8: Cell Counting Kit-8
CCNA2: Cyclin A2
CDC2: Cell division control protein 2
CDX: Cell line-derived xenograft
CETSA: Cellular thermal shift assay
ChIP: Chromatin immunoprecipitation
CI: Combination index
CRC: Colorectal cancer
Ct: Cycle threshold
CYP24A1: Cytochrome P450 family 24 subfamily A member 1
DMEM: Dulbecco's modified Eagle's medium
DMSO: Dimethyl sulfoxide
DNMTis: DNA methyltransferase inhibitors
EC_50_: half-maximal effective concentration
FBS: Fetal bovine serum
FDL: Fluor de Lys
GLUT1: Glucose transporter 1
GSEA: Gene Set Enrichment Analysis
HDAC: Histone deacetylase
H&E: Hematoxylin and eosin
HPLC: High-performance liquid chromatography
IC_50_: Half-maximum inhibitory concentration
IP: intraperitoneal
IV: Intravenous
KEGG: Kyoto Encyclopedia of Genes and Genomes
LDH: Lactate dehydrogenase
MSigDB: Molecular Signatures Database
NAD^+^: Nicotinamide adenine dinucleotide
OAADPr: 2'-O-acyl-ADP ribose
PCNA: Proliferating cell nuclear antigen
PDB: Protein Data Bank
PDO: Patient-derived organoid
PDK1: Pyruvate dehydrogenase 1
PDX: Patient-derived xenograft
PI: Propidium iodide
PKM2: Pyruvate kinase M2
RT-qPCR: Real-time quantitative polymerase chain reaction
SIRT6: Sirtuin6
STR: Short tandem repeat
SDS-PAGE: SDS-polyacrylamide gel electrophoresis
VD3: Vitamin D3
VDR: Vitamin D receptor
Wild-type: WT

## References

[1] Siegel RL, Miller KD, Jemal A (2019). Cancer statistics, 2019. CA Cancer J Clin.

[2] Kuipers EJ, Grady WM, Lieberman D, Seufferlein T, Sung JJ, Boelens PG (2015). Colorectal cancer. Nat Rev Dis Primers.

[3] Andre T, Boni C, Mounedji-Boudiaf L, Navarro M, Tabernero J, Hickish T (2004). Oxaliplatin, fluorouracil, and leucovorin as adjuvant treatment for colon cancer. N Engl J Med.

[4] Zamani M, Hosseini SV, Mokarram P (2018). Epigenetic biomarkers in colorectal cancer: premises and prospects. Biomarkers.

[5] Nakazawa T, Kondo T, Ma D, Niu D, Mochizuki K, Kawasaki T (2012). Global histone modification of histone H3 in colorectal cancer and its precursor lesions. Hum Pathol.

[6] Zhang Y, Nie L, Xu K, Fu Y, Zhong J, Gu K (2019). SIRT6, a novel direct transcriptional target of FoxO3a, mediates colon cancer therapy. Theranostics.

[7] Tang M, Lu X, Zhang C, Du C, Cao L, Hou T (2017). Downregulation of SIRT7 by 5-fluorouracil induces radiosensitivity in human colorectal cancer. Theranostics.

[8] Kugel S, Mostoslavsky R (2014). Chromatin and beyond: the multitasking roles for SIRT6. Trends Biochem Sci.

[9] Tasselli L, Zheng W, Chua KF (2017). SIRT6: Novel Mechanisms and Links to Aging and Disease. Trends Endocrinol Metab.

[10] Sebastian C, Zwaans BM, Silberman DM, Gymrek M, Goren A, Zhong L (2012). The histone deacetylase SIRT6 is a tumor suppressor that controls cancer metabolism. Cell.

[11] Kugel S, Sebastian C, Fitamant J, Ross KN, Saha SK, Jain E (2016). SIRT6 Suppresses Pancreatic Cancer through Control of Lin28b. Cell.

[12] Zhang G, Liu Z, Qin S, Li K (2015). Decreased expression of SIRT6 promotes tumor cell growth correlates closely with poor prognosis of ovarian cancer. Eur J Gynaecol Oncol.

[13] Chen X, Hao B, Liu Y, Dai D, Han G, Li Y (2014). The histone deacetylase SIRT6 suppresses the expression of the RNA-binding protein PCBP2 in glioma. Biochem Biophys Res Commun.

[14] Marquardt JU, Fischer K, Baus K, Kashyap A, Ma S, Krupp M (2013). Sirtuin-6-dependent genetic and epigenetic alterations are associated with poor clinical outcome in hepatocellular carcinoma patients. Hepatology.

[15] Azuma Y, Yokobori T, Mogi A, Altan B, Yajima T, Kosaka T (2015). SIRT6 expression is associated with poor prognosis and chemosensitivity in patients with non-small cell lung cancer. J Surg Oncol.

[16] Ming M, Han W, Zhao B, Sundaresan NR, Deng CX, Gupta MP (2014). SIRT6 promotes COX-2 expression and acts as an oncogene in skin cancer. Cancer Res.

[17] Lefort K, Brooks Y, Ostano P, Cario-Andre M, Calpini V, Guinea-Viniegra J (2013). A miR-34a-SIRT6 axis in the squamous cell differentiation network. EMBO J.

[18] Lu C-T, Hsu C-M, Lin P-M, Lai C-C, Lin H-C, Yang C-H (2014). The potential of SIRT6 and SIRT7 as circulating markers for head and neck squamous cell carcinoma. Anticancer Res.

[19] Liu Y, Xie QR, Wang B, Shao J, Zhang T, Liu T (2013). Inhibition of SIRT6 in prostate cancer reduces cell viability and increases sensitivity to chemotherapeutics. Protein Cell.

[20] Cagnetta A, Soncini D, Orecchioni S, Talarico G, Minetto P, Guolo F (2018). Depletion of SIRT6 enzymatic activity increases acute myeloid leukemia cells' vulnerability to DNA-damaging agents. Haematologica.

[21] Lin Z, Yang H, Tan C, Li J, Liu Z, Quan Q (2013). USP10 antagonizes c-Myc transcriptional activation through SIRT6 stabilization to suppress tumor formation. Cell Rep.

[22] Qi J, Cui C, Deng Q, Wang L, Chen R, Zhai D (2018). Downregulated SIRT6 and upregulated NMNAT2 are associated with the presence, depth and stage of colorectal cancer. Oncol Lett.

[23] Liu W, Wu M, Du H, Shi X, Zhang T, Li J (2018). SIRT6 inhibits colorectal cancer stem cell proliferation by targeting CDC25A. Oncol Lett.

[24] Pan PW, Feldman JL, Devries MK, Dong A, Edwards AM, Denu JM (2011). Structure and biochemical functions of SIRT6. J Biol Chem.

[25] Michishita E, McCord RA, Berber E, Kioi M, Padilla-Nash H, Damian M (2008). SIRT6 is a histone H3 lysine 9 deacetylase that modulates telomeric chromatin. Nature.

[26] Tasselli L, Xi Y, Zheng W, Tennen RI, Odrowaz Z, Simeoni F (2016). SIRT6 deacetylates H3K18ac at pericentric chromatin to prevent mitotic errors and cellular senescence. Nat Struct Mol Biol.

[27] Michishita E, McCord RA, Boxer LD, Barber MF, Hong T, Gozani O (2009). Cell cycle-dependent deacetylation of telomeric histone H3 lysine K56 by human SIRT6. Cell Cycle.

[28] Zhong L, D'Urso A, Toiber D, Sebastian C, Henry RE, Vadysirisack DD (2010). The histone deacetylase Sirt6 regulates glucose homeostasis via Hif1alpha. Cell.

[29] Sundaresan NR, Vasudevan P, Zhong L, Kim G, Samant S, Parekh V (2012). The sirtuin SIRT6 blocks IGF-Akt signaling and development of cardiac hypertrophy by targeting c-Jun. Nat Med.

[30] Ran LK, Chen Y, Zhang ZZ, Tao NN, Ren JH, Zhou L (2016). SIRT6 Overexpression Potentiates Apoptosis Evasion in Hepatocellular Carcinoma via BCL2-Associated X Protein-Dependent Apoptotic Pathway. Clin Cancer Res.

[31] Ghosh S, Liu B, Wang Y, Hao Q, Zhou Z (2015). Lamin A Is an Endogenous SIRT6 Activator and Promotes SIRT6-Mediated DNA Repair. Cell Rep.

[32] You W, Rotili D, Li T-M, Kambach C, Meleshin M, Schutkowski M (2017). Structural Basis of Sirtuin 6 Activation by Synthetic Small Molecules. Angew Chem Int Ed Engl.

[33] Schlicker C, Boanca G, Lakshminarasimhan M, Steegborn C (2011). Structure-based development of novel sirtuin inhibitors. Aging.

[34] Huang Z, Zhao J, Deng W, Chen Y, Shang J, Song K (2018). Identification of a cellularly active SIRT6 allosteric activator. Nat Chem Biol.

[35] Tong Z, Wang Y, Zhang X, Kim DD, Sadhukhan S, Hao Q (2016). SIRT7 Is Activated by DNA and Deacetylates Histone H3 in the Chromatin Context. ACS Chem Biol.

[36] Dirks WG, Faehnrich S, Estella IAJ, Drexler HG (2005). Short tandem repeat DNA typing provides an international reference standard for authentication of human cell lines. ALTEX.

[37] Molina DM, Jafari R, Ignatushchenko M, Seki T, Larsson EA, Dan C (2013). Monitoring Drug Target Engagement in Cells and Tissues Using the Cellular Thermal Shift Assay. Science.

[38] van de Wetering M, Francies HE, Francis JM, Bounova G, Iorio F, Pronk A (2015). Prospective derivation of a living organoid biobank of colorectal cancer patients. Cell.

[39] Subramanian A, Tamayo P, Mootha VK, Mukherjee S, Ebert BL, Gillette MA (2005). Gene set enrichment analysis: a knowledge-based approach for interpreting genome-wide expression profiles. Proc Natl Acad Sci U S A.

[40] Livak KJ, Schmittgen TD (2001). Analysis of relative gene expression data using real-time quantitative PCR and the 2(-Delta Delta C(T)) Method. Methods (San Diego, Calif).

[41] Chou TC (2010). Drug combination studies and their synergy quantification using the Chou-Talalay method. Cancer Res.

[42] Zhang J CY, Ruan C, Yang X, Wang C, Zhang Q, et al Compound Used As SIRT6 Small-molecule Allosteric Activator And Pharmaceutical Composition Thereof Patent PCT/CN2018/086766. 2019.2.14.

[43] Erickson JA, Jalaie M, Robertson Dh, Lewis Ra, Vieth M (2004). Lessons in molecular recognition: the effects of ligand and protein flexibility on molecular docking accuracy. J Med Chem.

[44] Zavodszky MI, Kuhn LA (2005). Side-chain flexibility in protein-ligand binding: the minimal rotation hypothesis. Protein Sci.

[45] Kong X, Pan P, Li D, Tian S, Li Y, Hou T (2015). Importance of protein flexibility in ranking inhibitor affinities: modeling the binding mechanisms of piperidine carboxamides as Type I1/2 ALK inhibitors. Phys Chem Chem Phys.

[46] Kourounakis AP, Xanthopoulos D, Tzara A (2020). Morpholine as a privileged structure: A review on the medicinal chemistry and pharmacological activity of morpholine containing bioactive molecules. Med Res Rev.

[47] Kumari A, Singh RK (2020). Morpholine as ubiquitous pharmacophore in medicinal chemistry: Deep insight into the structure-activity relationship (SAR). Bioorg Chem.

[48] Hu J, He B, Bhargava S, Lin H (2013). A fluorogenic assay for screening Sirt6 modulators. Org Biomol Chem.

[49] Zhang X, Khan S, Jiang H, Antonyak MA, Chen X, Spiegelman NA (2016). Identifying the functional contribution of the defatty-acylase activity of SIRT6. Nat Chem Biol.

[50] Sociali G, Liessi N, Grozio A, Caffa I, Parenti MD, Ravera S (2019). Differential modulation of SIRT6 deacetylase and deacylase activities by lysine-based small molecules. Mol Divers.

[51] Ardestani PM, Liang F (2012). Sub-cellular localization, expression and functions of Sirt6 during the cell cycle in HeLa cells. Nucleus.

[52] Bhardwaj A, Das S (2016). SIRT6 deacetylates PKM2 to suppress its nuclear localization and oncogenic functions. Proc Natl Acad Sci U S A.

[53] Prosser DE, Jones G (2004). Enzymes involved in the activation and inactivation of vitamin D. Trends Biochem Sci.

[54] Zheng R, Wan C, Mei S, Qin Q, Wu Q, Sun H (2019). Cistrome Data Browser: expanded datasets and new tools for gene regulatory analysis. Nucleic Acids Res.

[55] Pereira F, Larriba MJ, Munoz A (2012). Vitamin D and colon cancer. Endocr Relat Cancer.

[56] Ng K, Nimeiri HS, McCleary NJ, Abrams TA, Yurgelun MB, Cleary JM (2019). Effect of High-Dose vs Standard-Dose Vitamin D3 Supplementation on Progression-Free Survival Among Patients With Advanced or Metastatic Colorectal Cancer: The SUNSHINE Randomized Clinical Trial. JAMA.

[57] Ma Y, Zhang P, Wang F, Yang J, Liu Z, Qin H (2011). Association Between Vitamin D and Risk of Colorectal Cancer: A Systematic Review of Prospective Studies. J Clin Oncol.

[58] Meyer MB, Goetsch PD, Pike JW (2010). A downstream intergenic cluster of regulatory enhancers contributes to the induction of CYP24A1 expression by 1alpha,25-dihydroxyvitamin D3. J Biol Chem.

[59] Sun H, Jiang C, Cong L, Wu N, Wang X, Hao M (2018). CYP24A1 Inhibition Facilitates the Antiproliferative Effect of 1,25(OH)2D3 Through Downregulation of the WNT/beta-Catenin Pathway and Methylation-Mediated Regulation of CYP24A1 in Colorectal Cancer Cells. DNA Cell Biol.

[60] Kosa JP, Horvath P, Wolfling J, Kovacs D, Balla B, Matyus P (2013). CYP24A1 inhibition facilitates the anti-tumor effect of vitamin D3 on colorectal cancer cells. World J Gastroenterol.

[61] Jones PA, Issa JP, Baylin S (2016). Targeting the cancer epigenome for therapy. Nat Rev Genet.

[62] Parenti MD, Grozio A, Bauer I, Galeno L, Damonte P, Millo E (2014). Discovery of Novel and Selective SIRT6 Inhibitors. J Med Chem.

[63] Sociali G, Galeno L, Parenti MD, Grozio A, Bauer I, Passalacqua M (2015). Quinazolinedione SIRT6 inhibitors sensitize cancer cells to chemotherapeutics. Eur J Med Chem.

[64] Heger V, Tyni J, Hunyadi A, Horakova L, Lahtela-Kakkonen M, Rahnasto-Rilla M (2019). Quercetin based derivatives as sirtuin inhibitors. Biomed Pharmacother.

[65] Kokkonen P, Rahnasto-Rilla M, Mellini P, Jarho E, Lahtela-Kakkonen M, Kokkola T (2014). Studying SIRT6 regulation using H3K56 based substrate and small molecules. Eur J Pharm Sci.

[66] Kokkonen P, Rahnasto-Rilla M, Kiviranta PH, Huhtiniemi T, Laitinen T, Poso A (2012). Peptides and Pseudopeptides as SIRT6 Deacetylation Inhibitors. ACS Med Chem Lett.

[67] He B, Hu J, Zhang X, Lin H (2014). Thiomyristoyl peptides as cell-permeable Sirt6 inhibitors. Org Biomol Chem.

[68] Barker AJ, Gibson KH, Grundy W, Godfrey AA, Barlow JJ, Healy MP (2001). Studies leading to the identification of ZD1839 (iressa™): an orally active, selective epidermal growth factor receptor tyrosine kinase inhibitor targeted to the treatment of cancer. Bioorg Med Chem Lett.

[69] McKillop D, McCormick AD, Miles GS, Phillips PJ, Pickup KJ, Bushby N (2004). In vitro metabolism of gefitinib in human liver microsomes. Xenobiotica.

[70] Pike KG, Malagu K, Hummersone MG, Menear KA, Duggan HM, Gomez S (2013). Optimization of potent and selective dual mTORC1 and mTORC2 inhibitors: the discovery of AZD8055 and AZD2014. Bioorg Med Chem Lett.

[71] Guichard SM, Curwen J, Bihani T, D'Cruz CM, Yates JW, Grondine M (2015). AZD2014, an Inhibitor of mTORC1 and mTORC2, Is Highly Effective in ER+ Breast Cancer When Administered Using Intermittent or Continuous Schedules. Mol Cancer Ther.

[72] Hobaus J, Hummel DM, Thiem U, Fetahu IS, Aggarwal A, Mullauer L (2013). Increased copy-number and not DNA hypomethylation causes overexpression of the candidate proto-oncogene CYP24A1 in colorectal cancer. Int J Cancer.

[73] Sun H, Wang C, Hao M, Sun R, Wang Y, Liu T (2016). CYP24A1 is a potential biomarker for the progression and prognosis of human colorectal cancer. Hum Pathol.

[74] Hobaus J, Tennakoon S, Heffeter P, Groeschel C, Aggarwal A, Hummel DM (2016). Impact of CYP24A1 overexpression on growth of colorectal tumour xenografts in mice fed with vitamin D and soy. Int J Cancer.

[75] Nikolaou M, Pavlopoulou A, Georgakilas AG, Kyrodimos E (2018). The challenge of drug resistance in cancer treatment: a current overview. Clin Exp Metastasis.

[76] Crea F, Nobili S, Paolicchi E, Perrone G, Napoli C, Landini I (2011). Epigenetics and chemoresistance in colorectal cancer: an opportunity for treatment tailoring and novel therapeutic strategies. Drug Resist Updat.

