# Supplementary material for: Small-molecule activating SIRT6 elicits therapeutic effects and synergistically promotes anti-tumor activity of vitamin D3 in colorectal cancer
Source: Theranostics. 2020 Apr 27;10(13):5845–64. doi: 10.7150/thno.44043 (PMC7255010; doi:10.7150/thno.44043)
Supplement: Supplementary file 1 — Supplementary figures, data, and tables. [file thnov10p5845s1.pdf]

Supplemental Information

Small-molecule activating SIRT6 elicits therapeutic effects and synergistically promotes anti-tumor activity of vitamin D_3 in colorectal cancer

Jialin Shang[1,†], Zhehui Zhu[2,†], Yingyi Chen[1,†], Jinglue Song[2], Yuji Huang[2], Kun Song[1], Jie Zhong[1], Xinyuan Xu[1], Jiacheng Wei[1], Chengxiang Wang[1], Long Cui[2], Chen-Ying Liu[2,*], and Jian Zhang[1,3,4*]

[1]State Key Laboratory of Oncogenes and Related Genes, Shanghai Jiao-Tong University School of Medicine, Shanghai 200025, China.

[2]Department of Colorectal and Anal Surgery, Xinhua Hospital, Shanghai Jiao-Tong University School of Medicine, Shanghai 200092, China.

[3]Medicinal Bioinformatics Center, Shanghai Jiao-Tong University School of Medicine, Shanghai 200025, China.

[4]School of Pharmaceutical Sciences, Zhengzhou University, Zhengzhou 450001, China.

[†]These authors contributed equally to this work.

[*]Corresponding author.

CONTENTS

Supplementary Figures

Figure S1. Predicted binding modes of MDL-818, 813, 822, 821, 811 and 814 at the allosteric site on SIRT6.

Figure S2. MDL-800 and MDL-811 activated SIRT6 deacetylation on RHKK-Ac-AMC, as determined by FDL and HPLC assays.

Figure S3. Concentration-dependent effect of MDL-811 on SIRT6 demyristoylation.

Figure S4. Dose-dependent deacetylation effects of MDL-811 in CRC cell lines.

Figure S5. Cell cytotoxicity assays of CRC cell lines treated with MDL-811.

Figure S6. Cell proliferation of non-cancerous colon cell line treated with MDL-811.

Figure S7. Cell cycle distribution of CRC cell lines treated with MDL-811.

Figure S8. Body weights of xenograft models treated with MDL-811.

Figure S9. Effect of SIRT6 overexpression on gene expressions in HCT116 cells.

Figure S10. Effects of MDL-811 on the deacetylation of CYP24A1-binding histone marks in HCT116 cells.

Figure S11. Effect of CYP24A1 overexpression in HCT116 cells.

Figure S12. Effect of MDL-811 and 1,25$(OH)_2D_3$ cotreatment on CYP24A1 expression in CRC cell lines.

Figure S13. The superimposition of SIRT6 to SIRT1, SIRT2, SIRT3 and SIRT5 in cartoon mode.

Chemical Characterization

Synthesis of compounds MDL analogs.

NMR spectra of compounds MDL analogs.

HPLC analysis data of compounds MDL analogs.

Supplementary Tables

Table S1. Plasma pharmacokinetic parameters of MDL-800 and MDL-811 in C57BL/6J mice.

Table S2. Effect of MDL-811 among histone deacetylase enzymes.

Table S3. Melting temperatures of SIRT6 treated with or without MDL-811 in the CETSA.

Table S4. IC_{50} values of MDL-811 in CRC cell lines.

Table S5. Statistical data of cell cycle phase distribution in CRC cell lines treated with MDL-811 for 48 h.

Table S6. Primary antibodies used for western blots.

Table S7. Primer sequences used for RT-qPCR.

Table S8. Primary antibodies used for ChIP assays.

Table S9. Primer sequences used for ChIP-qPCR.

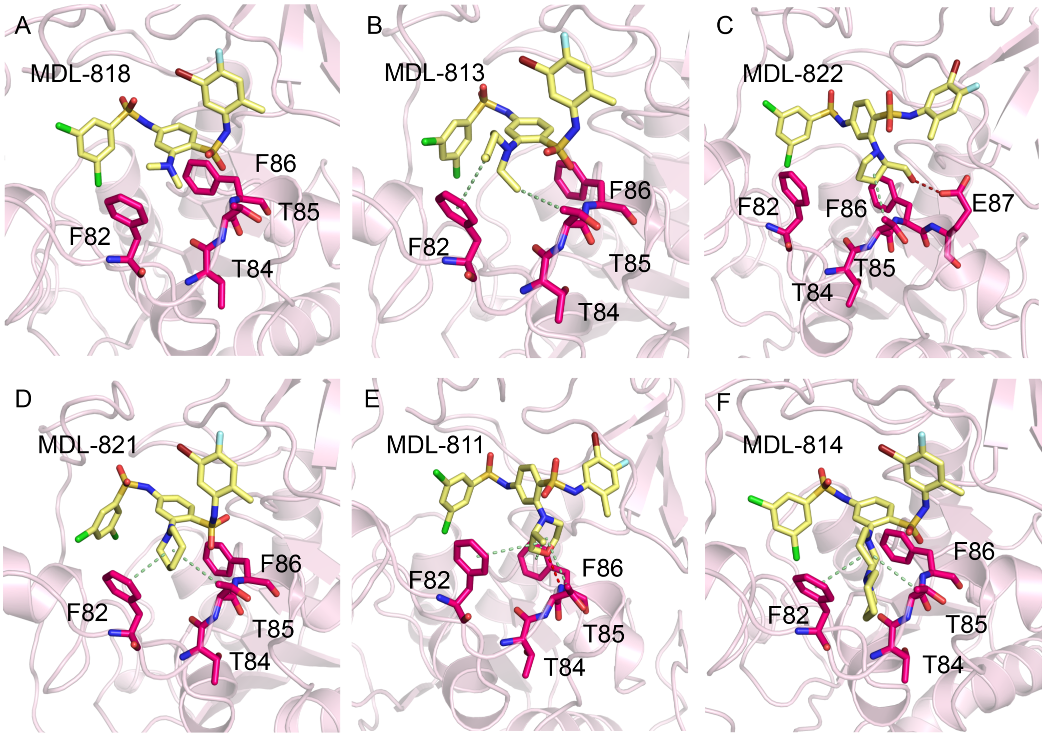

Figure S1. Predicted binding modes of MDL-818, 813, 822, 821, 811 and 814 at the allosteric site on SIRT6. The compounds and pocket residues are displayed as sticks and colored in yellow and hotpink. Hydrogen bonds are shown as red dashed lines and hydrophobic interactions are shown as palegreen dashed lines.

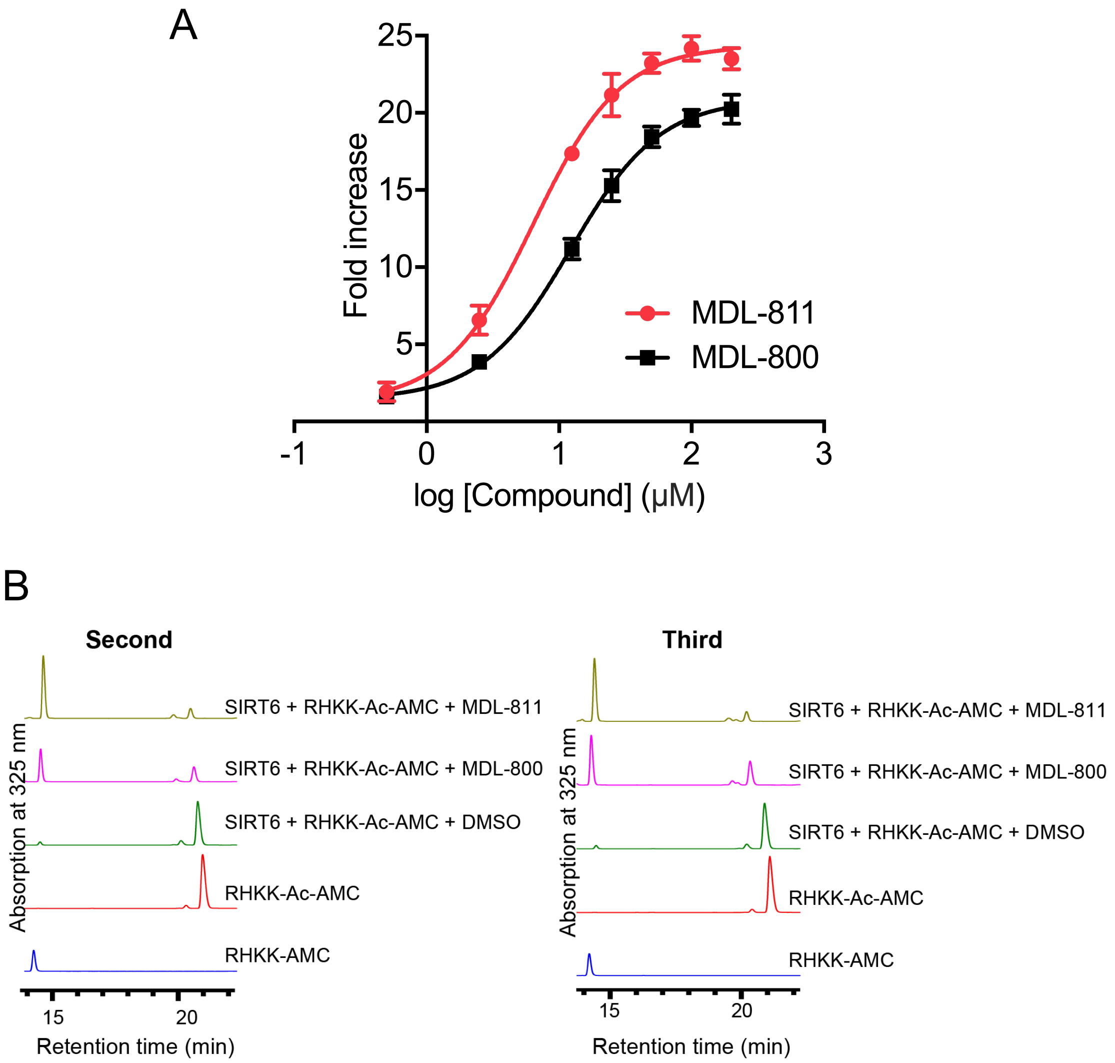

Figure S2. MDL-800 and MDL-811 activated SIRT6 deacetylation on RHKK-Ac-AMC, as determined by FDL and HPLC assays. (A) Concentration-dependent effects of MDL-800/MDL-811 on the activation of SIRT6 deacetylation were assessed by FDL assays with the acetylated peptide RHKK-Ac-AMC (75 μM). The data are shown as mean ± s.d. from three independent experiments (MDL-811: EC_{50} = 5.7 ± 0.8 μM; MDL-800: EC_{50} = 12.3 ± 0.7 μM). (B) HPLC traces of SIRT6 deacetylation on RHKK-Ac-AMC in the absence or presence of MDL-800/MDL-811 at 25 μM. Data are the other two of three independent experiments.

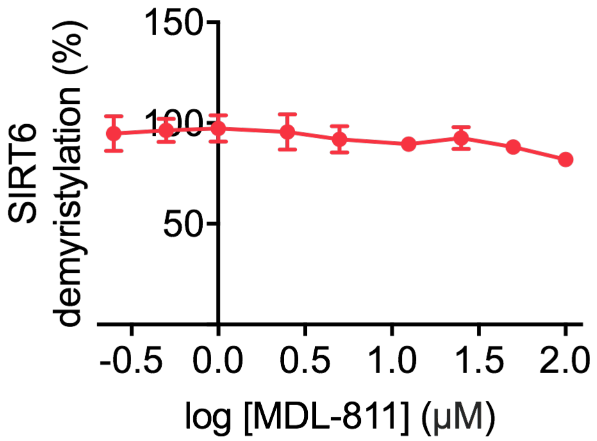

Figure S3. Concentration-dependent effect of MDL-811 on SIRT6 demyristoylation. The effect of MDL-811 was assessed by FDL assay with a TNFα peptide containing a myristoyl group (EALPKK-Myr-AMC) at various concentrations of MDL-811. The data are shown as mean ± s.d. from three independent experiments.

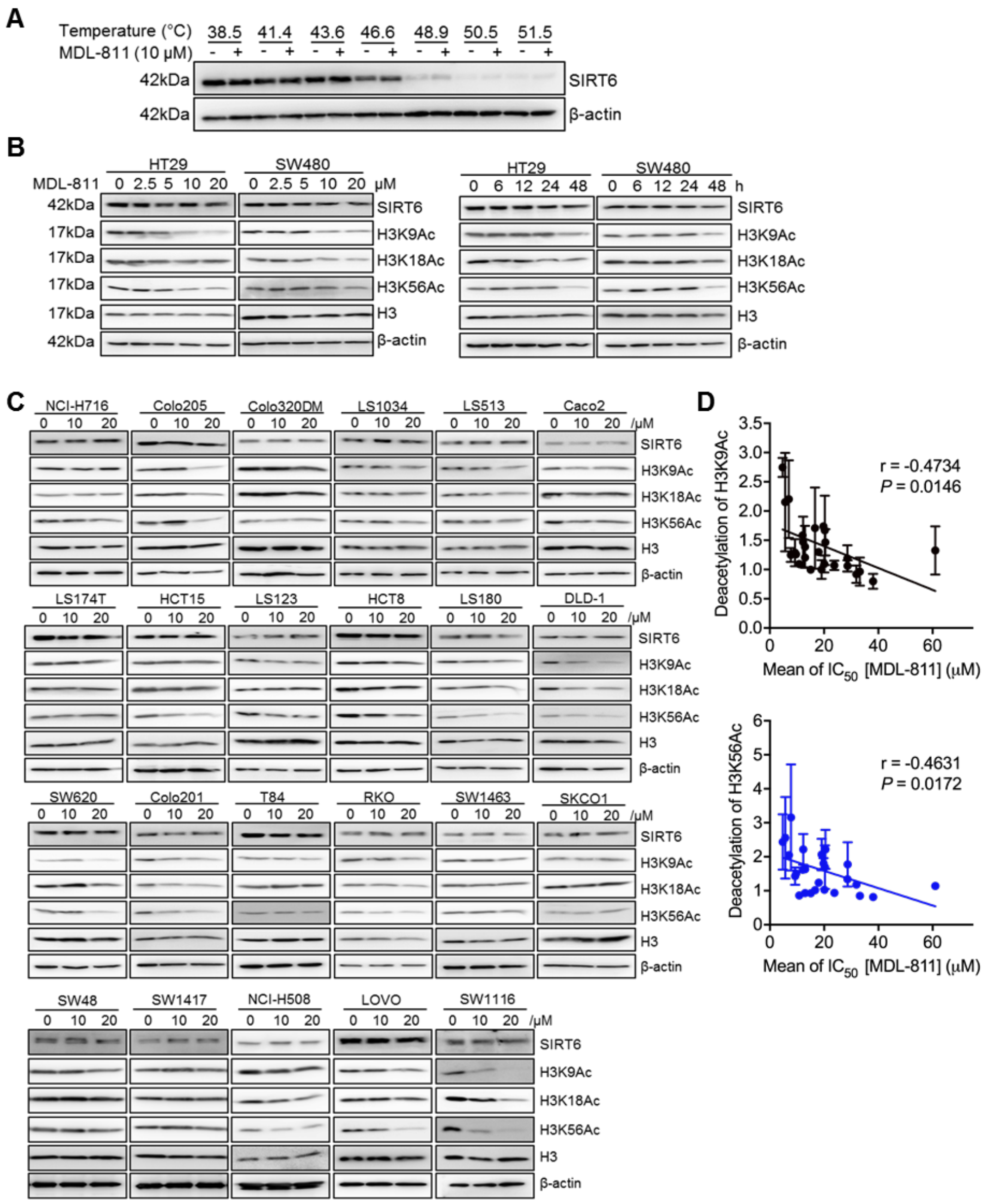

Figure S4. Dose-dependent deacetylation effects of MDL-811 in CRC cell lines. (A) Representative western blots indicating the thermal stabilization of the endogenous SIRT6 protein. The CETSA was performed in intact HCT116 cells in the absence or presence of 10 μM MDL-811 for 24 h. (B) Representative western blots of SIRT6, H3K9Ac, H3K18Ac, and H3K56Ac in HT29 and SW480 cells treated with the indicated concentrations of MDL-811 for 48 h, or treated with 10 μM MDL-811 at the indicated times. (C) Representative western blots of SIRT6, H3K9Ac, H3K18Ac, and H3K56Ac in various CRC cell lines treated with indicated concentrations of MDL-811 for 48 h. Histone H3 was used as the internal control, and β-actin was used as the loading control. (D) Pearson correlation analysis between the IC_{50} values of MDL-811 at 48 h and the levels of H3K9Ac or H3K56Ac deacetylation in various CRC cell lines after treatment with 10 μM MDL-811. Quantification of deacetylation levels was performed with ImageJ V4. Each deacetylation level was normalized to the corresponding histone H3 level. Each dot represents the mean ± s.e.m. of two or three independent experiments in n = 26 CRC cell lines. The data were subjected to Pearson correlation analysis (10 μM MDL-811: H3K9Ac, r = -0.4734, $P = 0.0146$; H3K56Ac, r = -0.4631, $P = 0.0172$).

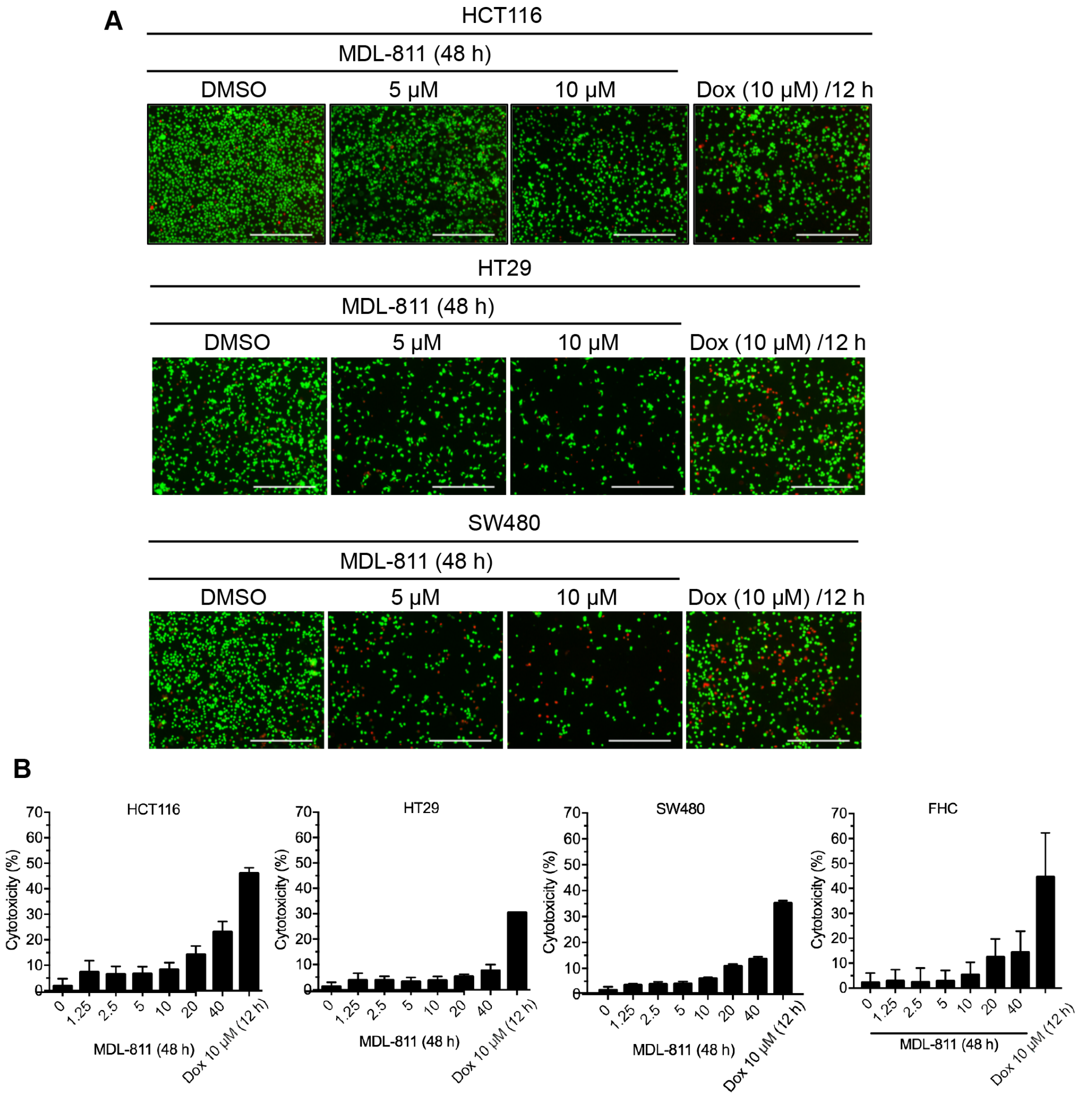

Figure S5. Cell cytotoxicity assays of CRC cell lines treated with MDL-811. (A) Live-dead double staining assays of CRC cells (HCT116, HT29, and SW480) treated with DMSO for 48 h, with 5 or 10 μM MDL-811 for 48 h or with 10 μM doxorubicin for 12 h. Live cells were stained with the green fluorescent dye Calcein-AM, and dead cells were stained with the red dye propidium iodide. The images are representative fields from two independent experiments. Representative images (10× magnification) are shown. Scale bars, 100 μm. (B) Cytotoxicity of MDL-811 to CRC cell lines (HCT116, HT29, SW480) and non-cancerous colon cell line FHC was confirmed by measuring the release of the cytosolic marker LDH. Cells were treated with the indicated concentrations of MDL-811 for 48 h or with 10 μM doxorubicin for 12 h. The data are presented as the mean ± s.d. of three independent experiments.

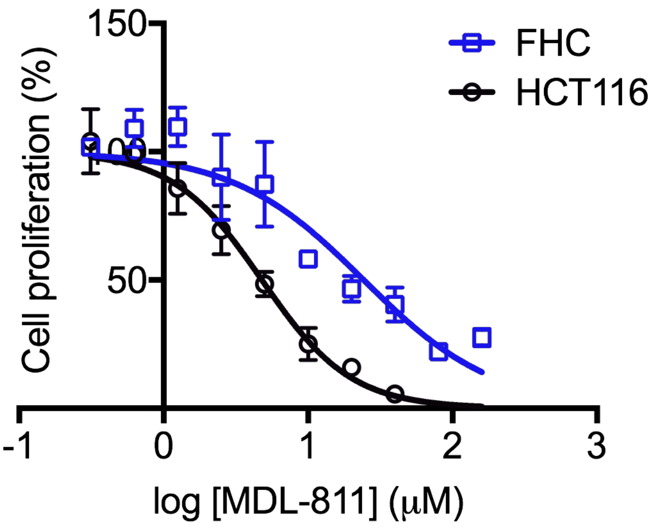

Figure S6. Cell proliferation of non-cancerous colon cell line treated with MDL-811. Dose response of the proliferation of non-cancerous colon cell line FHC and CRC cell line HCT116 exposed to MDL-811 for 48 h was normalized to the proliferation of the corresponding DMSO-treated controls. Cell proliferation was determined by CCK-8 assays. The data indicate the mean ± s.e.m. of three independent experiments (FHC: IC_{50} = 24.4 ± 0.5 μM; HCT116: IC_{50} = 4.8 ± 0.2 μM).

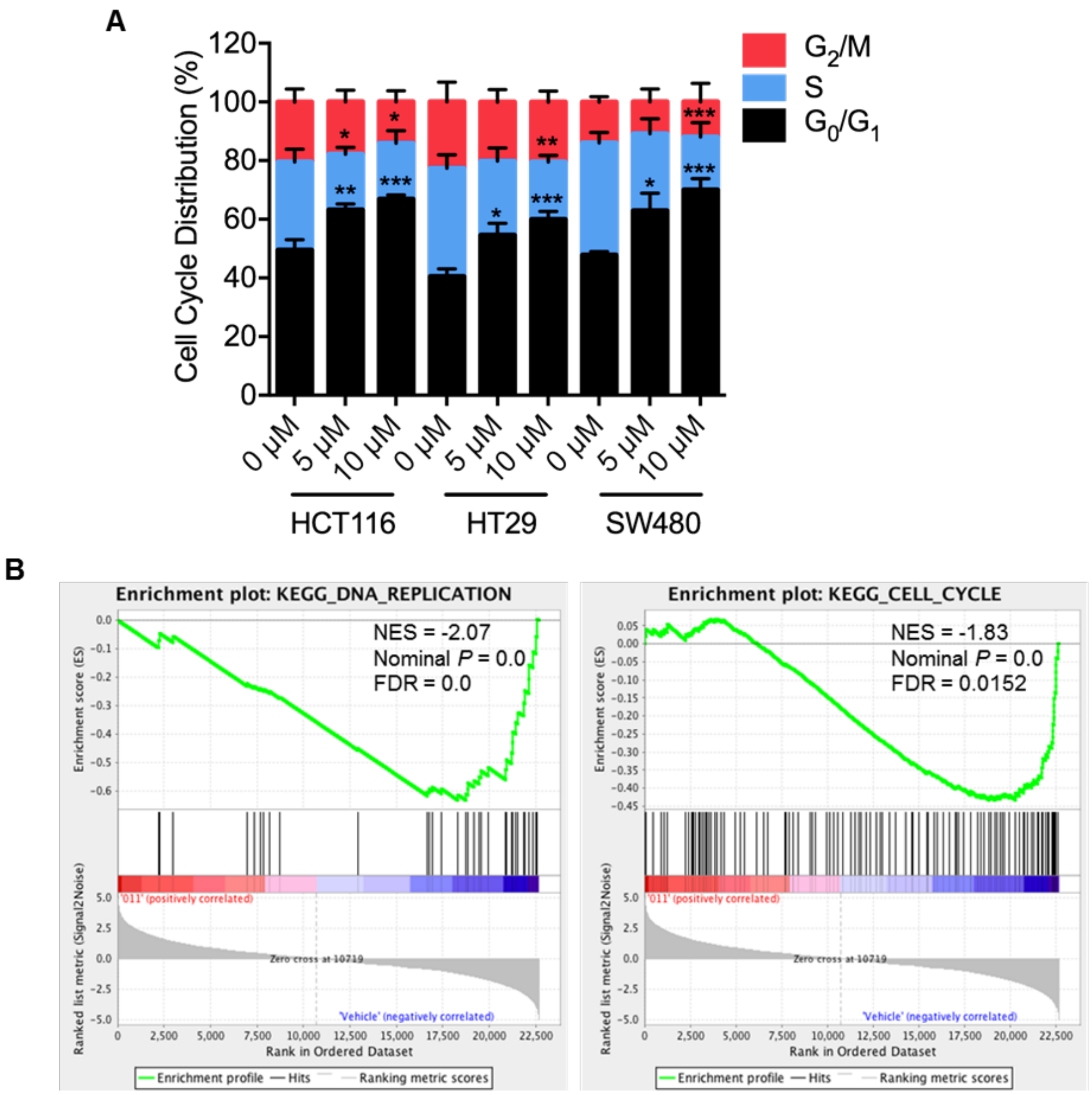

Figure S7. Cell cycle distribution of CRC cell lines treated with MDL-811. (A) Cell cycle distribution of HCT116, HT29, and SW480 cells treated with DMSO or with 5 or 10 μM MDL-811 for 48 h, as measured by PI staining. The data are presented as the mean ± s.d. of three independent experiments (*, $P < 0.05$; **, $P < 0.01$; ***, $P < 0.001$; two-way ANOVA). (B) GSEA plots showing enrichment of gene expression changes by exposure to 10 μM MDL-811 for 48 h in the DNA REPLICATION and CELL CYCLE signatures from the MSigDB v6.2 collection C2 curated gene sets (CP: KEGG gene sets). Positive normalized enrichment scores (NESs) indicate upregulation of gene sets, whereas negative NESs indicate downregulation of gene sets. FDR, false discovery rate.

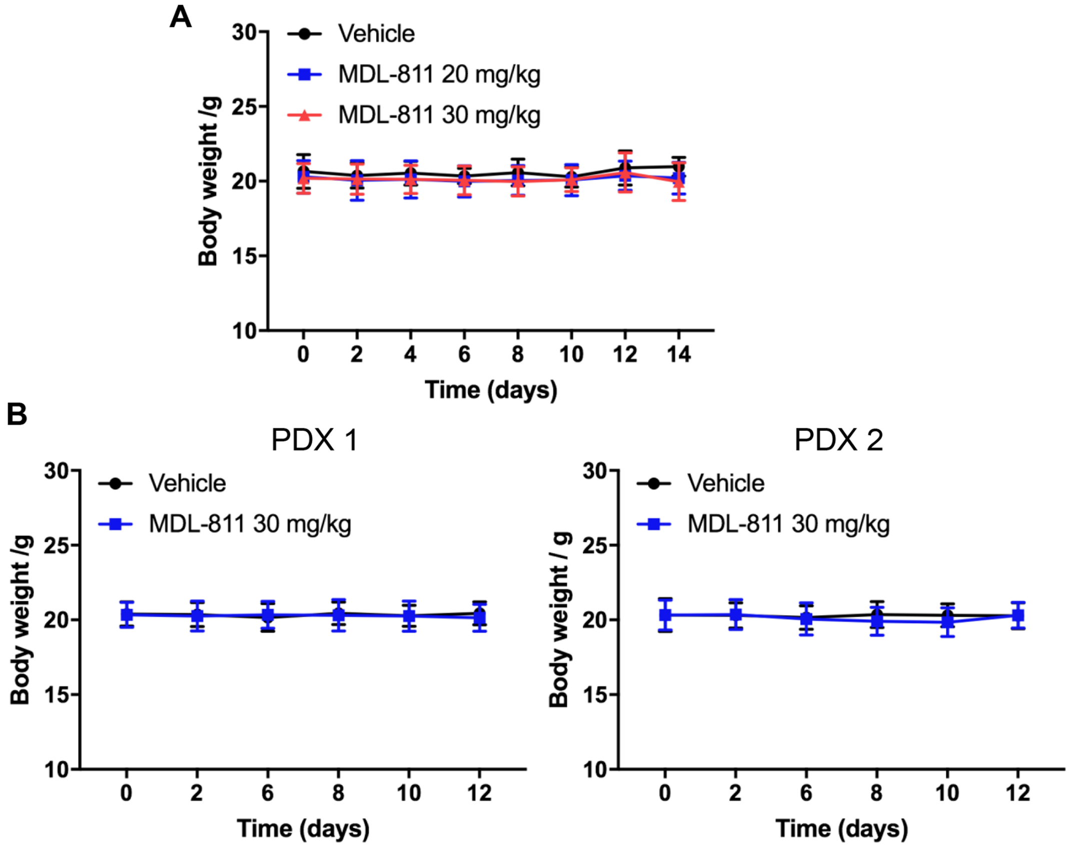

Figure S8. Body weights of xenograft models treated with MDL-811. (A) Body weights of HCT116 CDX mice treated with either vehicle or MDL-811 are shown (n = 6 mice per group). (B) Body weights of PDX mice treated with either vehicle or MDL-811 are shown (n = 8 mice per group). The data is plotted as the mean ± s.d..

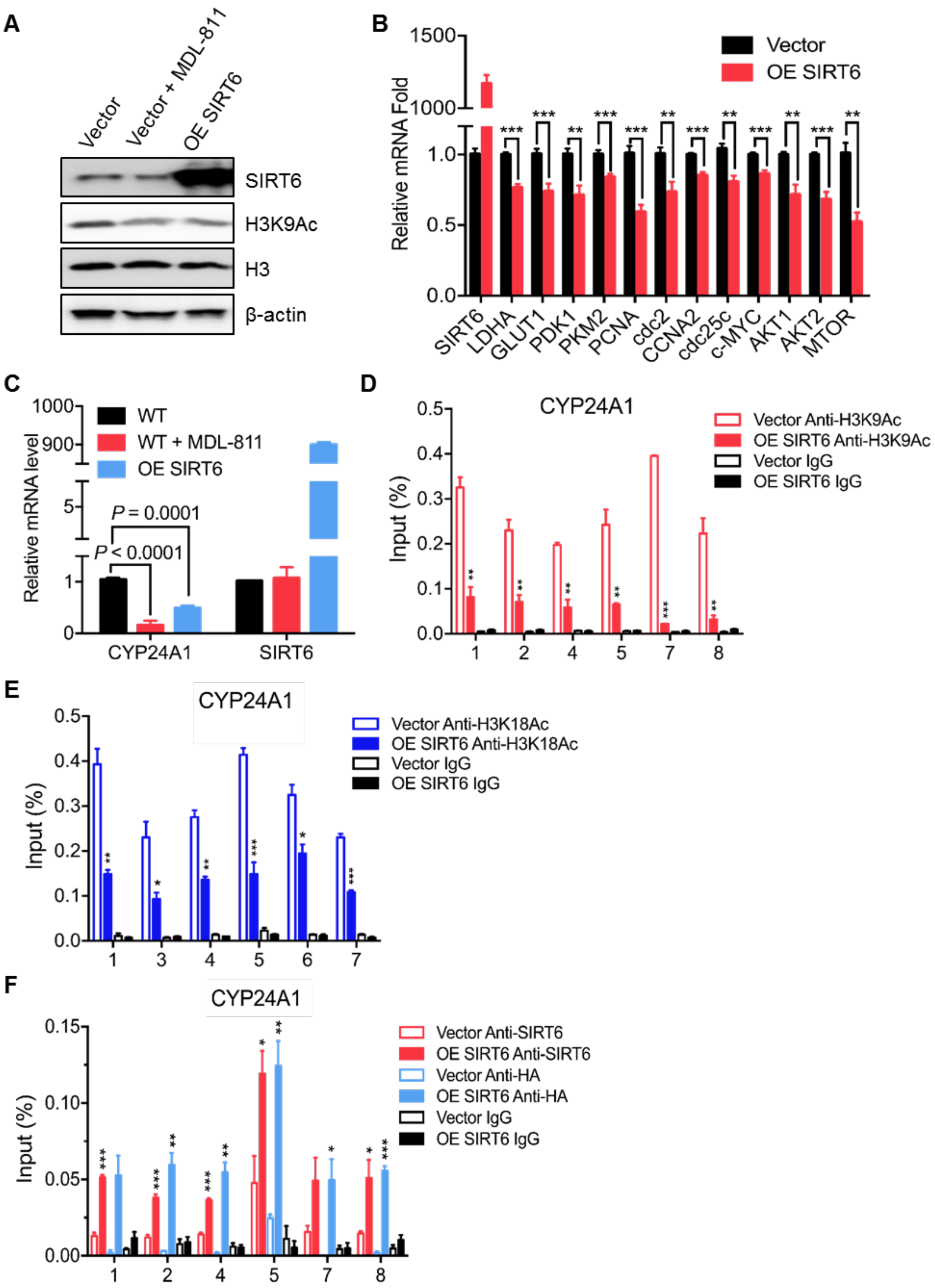

Figure S9. Effect of SIRT6 overexpression on gene expressions in HCT116 cells. (A) Western blots showing effects on HCT116 cells transiently transfected with vector or pLVX-SIRT6-HA. (B) RT-qPCR analyses showing the mRNA levels of candidate genes in HCT116 cells transiently transfected with vector or pLVX-SIRT6-HA before being treated with 10 μM MDL-811 for 48 h. The data are normalized to the β-actin levels and presented as mean ± s.e.m. of two or three independent experiments (*, $P < 0.05$; **, $P < 0.01$; ***, $P < 0.001$, two-tailed student's unpaired t-test). (C) RT-qPCR analyses showing the mRNA levels of CYP24A1 and SIRT6 in HCT116 cells

transfected with vector or pLVX-SIRT6-HA and then treated with DMSO or 10 μM MDL-811 for 48 h. The data are normalized to the β-actin levels and presented as mean ± s.d. of three experiments. (D), (E) and (F) ChIP assays of HCT116 cells transfected with vector or pLVX-SIRT6-HA. Anti-H3K9Ac (D), anti-H3K18Ac (E), anti-SIRT6 or anti-HA (F) antibodies were used to detect the deacetylation levels of these residues in the indicated regions of CYP24A1. The data are presented as the mean values of the percentage of input ± s.e.m. from one of two independent experiments with technical triplicates (*, $P < 0.05$; **, $P < 0.01$; ***, $P < 0.001$; two-tailed unpaired Student's t-test).

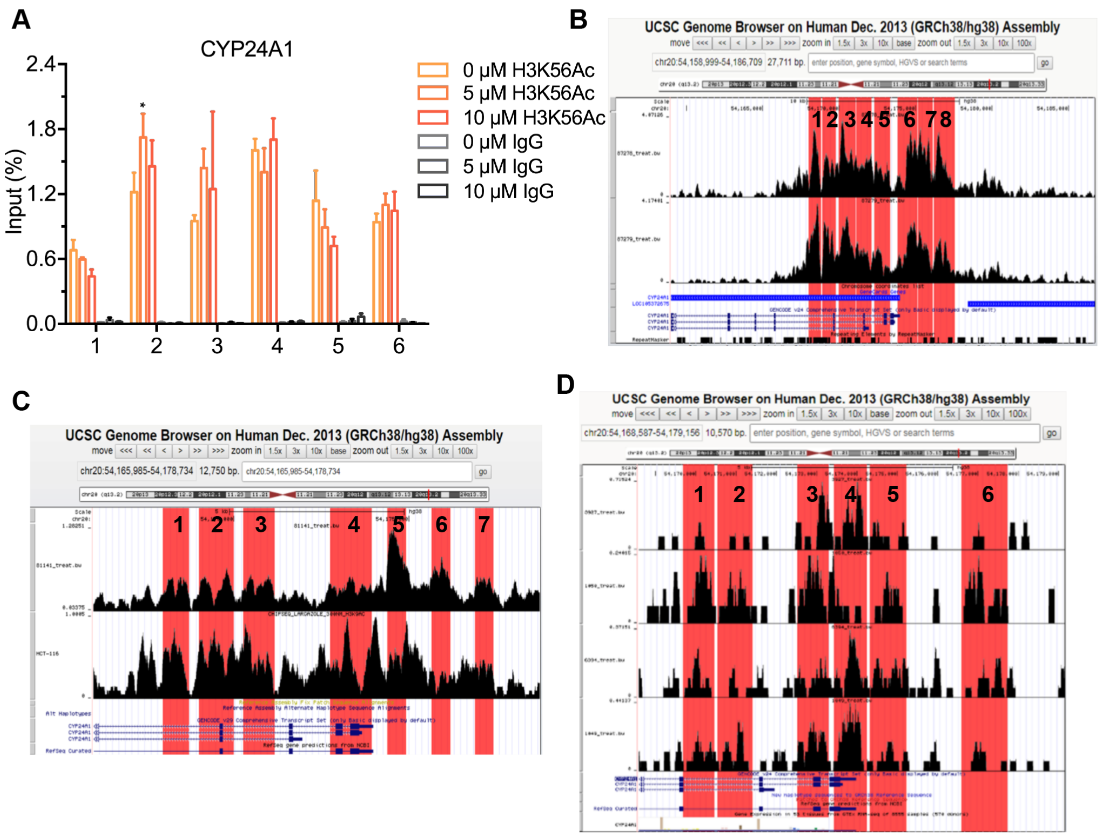

Figure S10. Effects of MDL-811 on the deacetylation of CYP24A1-binding histone marks in HCT116 cells. (A) ChIP assays using anti-H3K56Ac antibody to assess H3K56Ac occupancy in the indicated regions of CYP24A1 in HCT116 cells treated with DMSO or with 5 or 10 μM MDL-811. The data are presented as the mean values of the percentage of input ± s.e.m. from n = 3 independent experiments with technical triplicates. *P* values were determined by two-way ANOVA (*, $P < 0.05$). (B), (C) and (D) ChIP-qPCR primers were designed based on the CYP24A1 DNA sequence of peak regions of H3K9Ac (B), H3K18Ac (C) or H3K56Ac (D) using ChIP-seq data extracted from the Cistrome database.

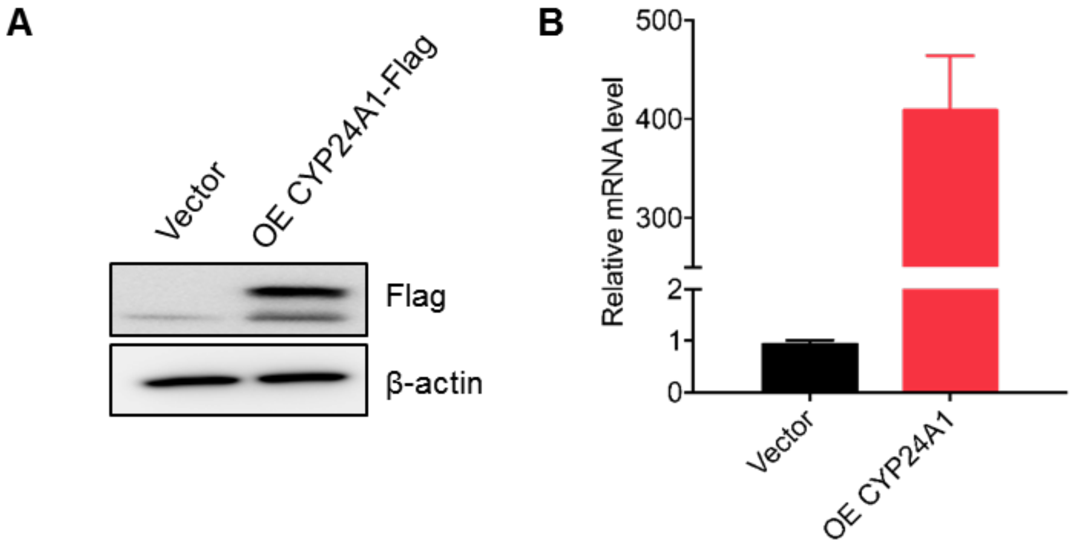

Figure S11. Effect of CYP24A1 overexpression in HCT116 cells. Western blots (A) and RT-qPCR (B) analysis of HCT116 cells transiently transfected with vector or pcDNA3.1-CYP24A1-Flag for 48 h. The data are normalized to the β-actin levels and presented as mean ± s.e.m. of two experiments.

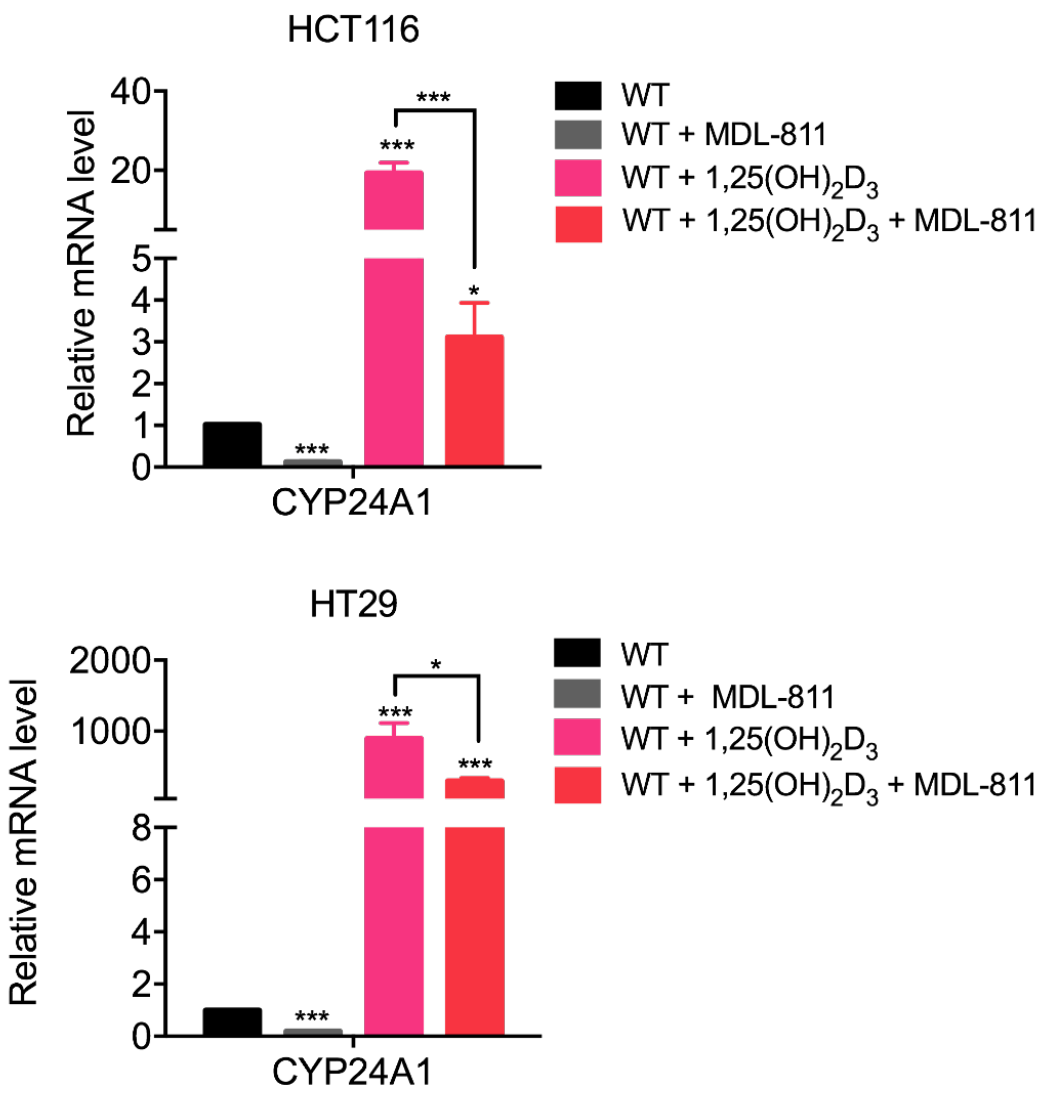

Figure S12. Effect of MDL-811 and 1,25$(OH)_2D_3$ cotreatment on CYP24A1 expression in CRC cell lines. RT-qPCR analysis showing the mRNA levels of CYP24A1 in HCT116 and HT29 cells treated with MDL-811 (5 μM), 1,25$(OH)_2D_3$ (5 μM) or the combination for 48 h. The data are normalized to the β-actin levels and presented as mean ± s.e.m of three experiments (*, $P < 0.05$; **, $P < 0.01$; ***, $P < 0.001$, two-tailed student's unpaired t-test).

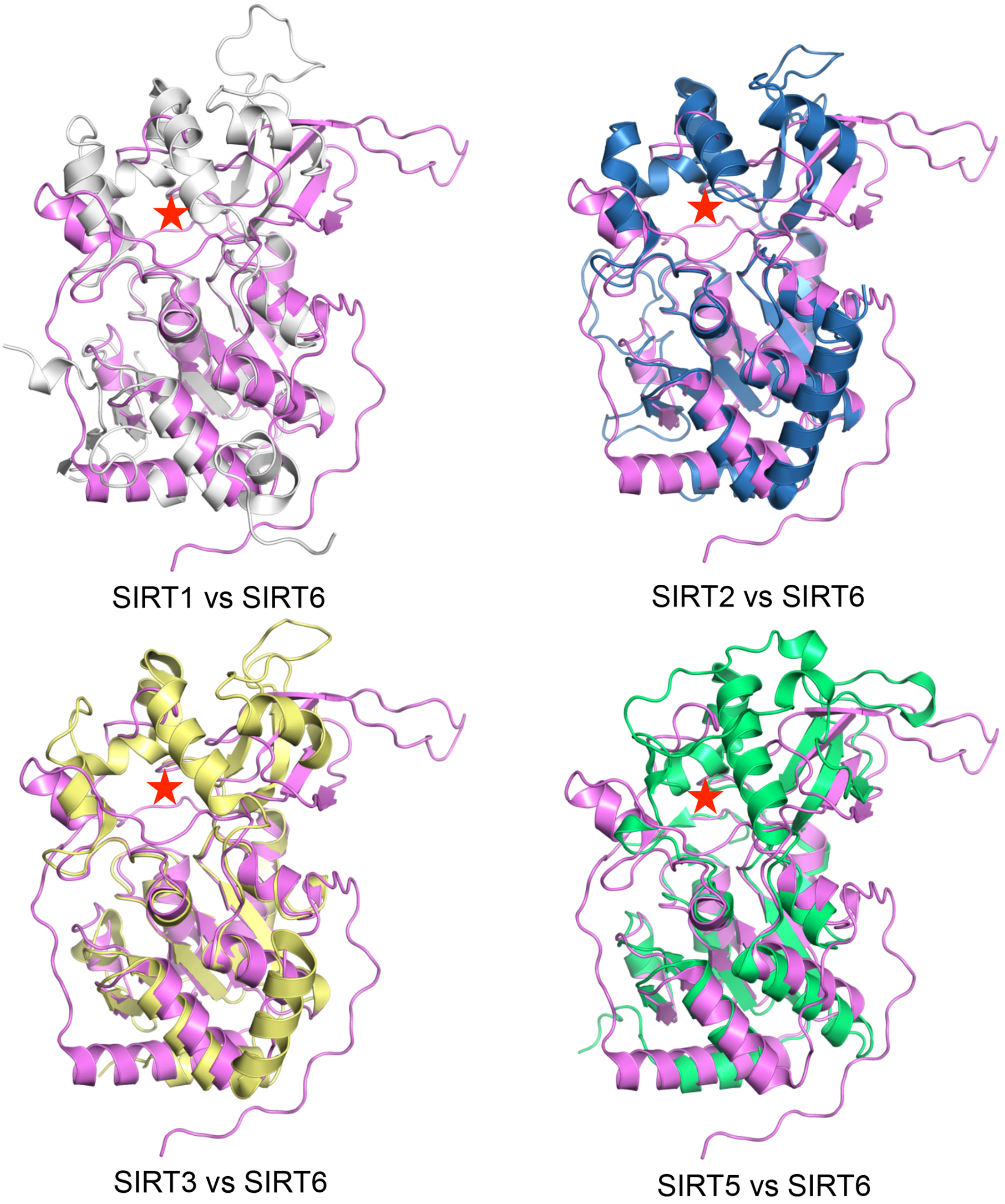

Figure S13. The superimposition of SIRT6 (violet) to SIRT1 (white), SIRT2 (skyblue), SIRT3 (paleyellow) and SIRT5 (limegreen) in cartoon mode. The allosteric site for MDL-811 is represented as red pentagram. The PDB codes of the SIRT1, SIRT2, SIRT3, SIRT5, and SIRT6 structures are 4KXQ, 3ZGV, 4BN4, 3RIY, and 5Y2F, respectively.

Chemical Characterization
Synthesis of Compounds MDL analogs

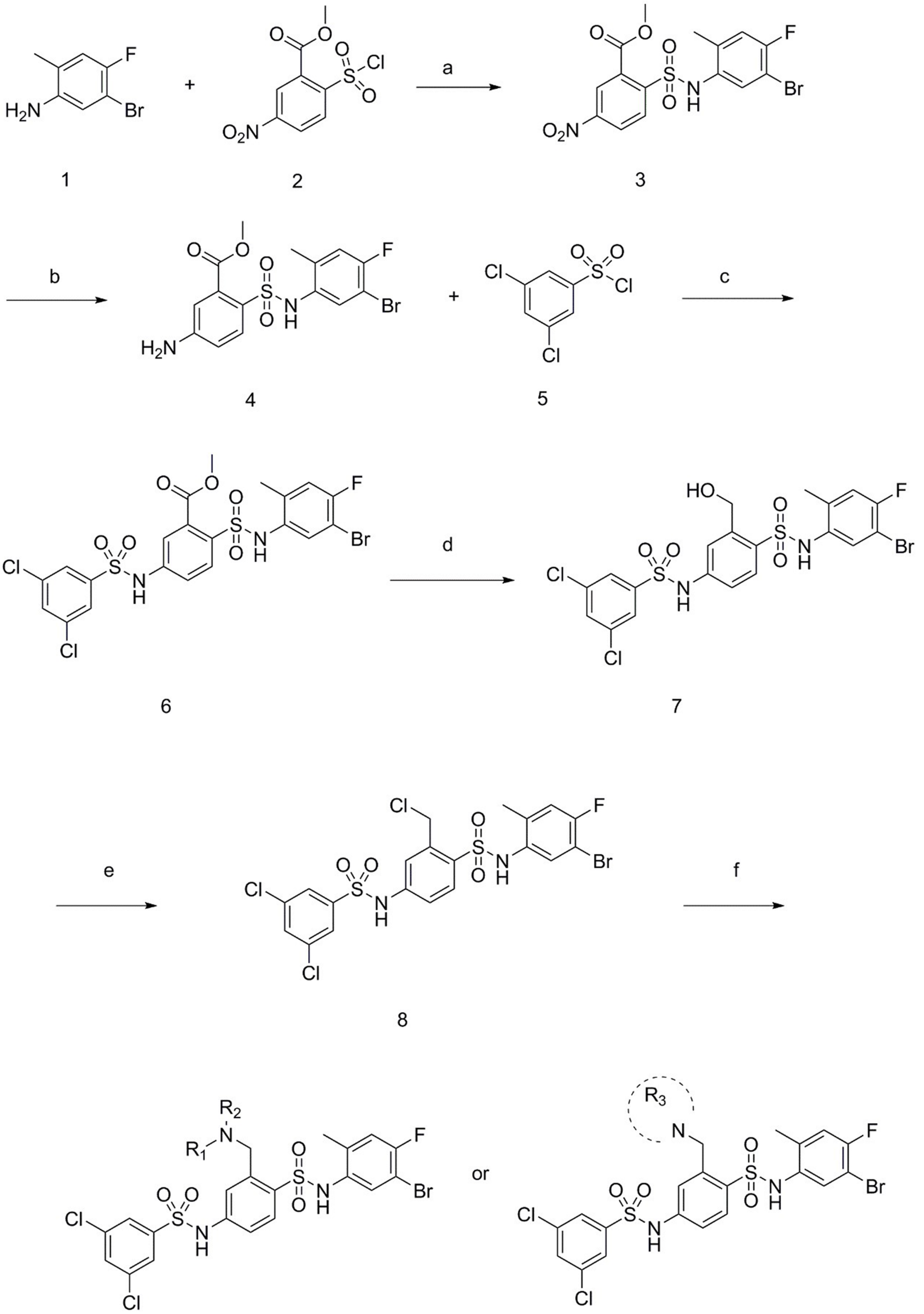

[a]Reagents and conditions: (a) pyridine, room temperature,8 h; (b) Fe, CH_3COOH, 50 °C 12 h; (c) pyridine, room temperature, 6 h; (d) LAH, THF, 50 °C, 12 h; (e) $SOCl_2$, 80 °C, 10 h; (f) secondary amine, THF, 50 °C, 6 h.

Methyl 2-(*N*-(5-bromo-4-fluoro-2-methylphenyl)sulfamoyl)-5-nitrobenzoate (3). To a solution of 5-bromo-4-fluoro-2-methylaniline (20 g, 98.0 mmol, **1**) in 20 mL pyridine was added methyl 2-(chlorosulfonyl)-5-nitrobenzoate (32.9 g, 117.6 mmol, **2**) under 0 °C and the reaction was stirred at the same temperature for about 1 hour. Then the reaction was moved to 25 °C and stirred for another 8 h. The reaction was cooled to 0 °C and adjusted the pH to 3-4 with 1 N hydrochloric acid solution. The precipitate formed was filtered, washed with water and dried to yield crude intermediate product 5, which was directly used to the next step without any purification. ^{1}H NMR (400 MHz, DMSO-d$_6$) δ 10.08 (s, 1H), 8.50-8.47 (m, 2H), 7.98-7.96 (dd, *J* = 2.0 Hz, *J* = 8.0 Hz, 1H), 7.36-7.34 (d, *J* = 8.0 Hz, 1H), 7.29-7.27 (d, *J* = 8.0 Hz, 1H), 3.78 (s, 3H), 1.96 (s, 3H).

Methyl 5-amino-2-(*N*-(5-bromo-4-fluoro-2-methylphenyl)sulfamoyl)benzoate (4). The crude intermediate product **3** (38.59 g, 86.53 mmol) dissolved in acetic acid was added iron powder (29.1 g, 519 mmol) at 50 °C. Then the solution was stirred at the same condition for 12 h. The system was filtered and the solvent was evaporated under reduced pressure. The residue was purified by column chromatography on silica gel (hexane: ethyl acetate = 2:1) to afford intermediate product **4** (29.66 g, two steps 73%) as a white powder. ^{1}H NMR (400 MHz, DMSO-d$_6$) δ 8.89 (s, 1H), 7.30-7.20 (m, 2H), 6.73 (s, 1H), 6.64-6.59 (m, 2H), 6.30 (s, 2H), 3.73 (s, 3H), 2.02 (s, 3H).

Methyl 2-(*N*-(5-bromo-4-fluoro-2-methylphenyl)sulfamoyl)-5-(3,5-dichlorophenylsulfonamido)benzoate (6). A solution of methyl 5-amino-2-(*N*-(5-bromo-4-fluoro-2-methylphenyl) sulfamoyl) benzoate (**4**, 13.9 g, 33.32 mmol) in 30 mL pyridine was added 3,5-dichlorobenzene-1-sulfonyl chloride (**5**, 16.45 g, 58.8 mmol) under 0 °C and the reaction was still stirred at the same temperature for about 1 hour. Then the reaction was moved to 25 °C and stirred for another 6 h. The reaction was cooled to 0 °C, and adjusted the pH to 3-4 with 2 N hydrochloric acid solution. The precipitate formed was filtered and subsequently purified by column chromatography on silica gel (hexane: ethyl acetate = 3:1) to afford **6** (17.6 g) as a white powder. Yield: 85%. ^{1}H NMR (400 MHz, DMSO-*d$_6$*) δ 11.36 (s, 1H), 9.46 (s, 1H), 8.01 (s, 1H), 7.81 (d, *J* = 2.0 Hz, 2H), 7.60-7.58 (d, *J* = 8.0 Hz, 1H), 7.44-7.40 (dd, *J* = 2.0 Hz, *J* = 8 Hz, 1H), 7.27 (d, *J* = 2.0 Hz, 1H), 7.24-7.22 (d, *J* = 8.0 Hz, 1H), 7.18-7.16 (d, *J* = 8.0 Hz, 1H), 3.74 (s, 3H), 1.82 (s, 3H).

***N*-(5-bromo-4-fluoro-2-methylphenyl)-4-(3,5-dichlorophenylsulfonamido)-2 (hydroxymethyl)benzenesulfonamide (7).** Within a flask was dissolved methyl 2-(*N*-(5-bromo-4-fluoro-2-methylphenyl)sulfamoyl)-5-(3,5-dichlorophenylsulfonamido)benzoate (**6**, 1 g, 1.59 mmol) in 20 mL of tetrahydrofuran. Then was added Lithium aluminum hydride (242 mg, 6.38 mmol) slowly added at ice bath and the contents were heated at 60 °C for about 12 h. The reaction was allowed to cool to room temperature and poured into ice bath, and adjusted the pH to 2-3 with 3N hydrochloric acid solution. The precipitate formed was filtered and dried to yield crude **7** as a yellow solid without any purification. ^{1}H NMR (DMSO-d$_6$, 400 MHz) δ 11.09 (s, 1H), 9.74 (s, 1H), 8.04-8.03 (t, 1H), 7.79-7.78 (d, 2H), 7.63-7.62 (d, *J* = 4.0

Hz, 1H), 7.45-7.43 (d, J = 8.0 Hz, 1H), 7.16-7.06 (m, 3H), 5.58-5.55 (t, 1H), 4.75-4.74 (d, J = 4.0 Hz. 2H), 1.77 (s, 3H). LRMS (ESI-) 594.9 (M-H).

***N*-(5-bromo-4-fluoro-2-methylphenyl)-2-(chloromethyl)-4-(3,5-dichlorophenylsulfonamido)benzenesulfonamide (8).** *N*-(5-bromo-4-fluoro-2-methylphenyl)-4-(3,5-dichlorophenylsulfonamido)-2-(hydroxymethyl)benzenesulfonamide (**7,** 580 mg, 0.969 mmol) was added 10 mL of thionyl chloride slowly at ice bath and the contents were heated at 60 °C for about 10 h. After the removal of the excess reagent, the crude product was recrystallized with ethyl acetate to afford 8 as a white solid (420 mg, two step 43%). ^{1}H NMR (DMSO-d_6, 400 MHz) δ 11.25 (s, 1H), 9.95 (s, 1H), 8.05-8.04 (t, 1H), 7.81-7.80 (d, 2H), 7.56-7.50 (m, 2H), 7.21-7.09 (m,3H), 4.99 (s, 2H), 1.78 (s, 3H) LRMS (ESI-) 612.8 (M-H).

(*R*)-*N*-(5-bromo-4-fluoro-2-methylphenyl)-4-(3,5-dichlorophenylsulfonamido)-2-((3-methylmorpholino)methyl)benzenesulfonamide (MDL-811). *N*-(5-bromo-4-fluoro-2-methylphenyl)-2-(chloromethyl)-4-(3,5-dichlorophenylsulfonamido)benzenesulfonamide (**8**, 100 mg, 0.162 mmol)was dissolved in 5 mL of dried THF, and then (R)-3-methylmorpholine (33 mg, 0.324 mmol) and TEA (41 mg, 0.405 mmol) was added. The reaction was stirred at 50 °C for about 6 h. After concentration under reduced pressure, the residue was partitioned between ethyl acetate and water. The organic layer was further washed with saturated aq $NaHCO_3$, brine, dried over sodium sulfate, evaporated in vacuo, and subsequently purified by column chromatography on silica gel (ether: ethyl acetate = 3:1) to afford **MDL-811** as a white solid (80 mg, 73%). ^{1}H NMR (DMSO-d_6, 400 MHz) δ 11.07 (s, 1H), 9.86 (s, 1H), 8.04 (s, 1H), 7.77 (s, 2H), 7.71 (s, 1H), 7.59-7.57 (d, J = 8.0 Hz, 1H), 7.20-7.11 (m, 2H), 7.01-7.99 (d, J = 8.0 Hz, 1H), 3.98-3.94 (d, J = 16.0 Hz, 1H), 3.69-3.67 (m, 2H), 3.50-3.46 (m, 2H), 3.22-3.17 (m, 1H), 2.51-2.47 (m, 1H), 2.34-2.32 (d, J = 8.0 Hz, 1H), 2.08 (s, 2H), 1.82 (s, 3H), 0.85-0.84 (d, J = 4.0 Hz, 3H). ^{13}C NMR (DMSO-d_6 100 MHz) 156.7, 154.3, 140.9, 140.1, 139.0, 136.4 (J = 7), 134.1, 131.9 (J = 2), 131.7 (J = 5), 130.5 (J = 2), 129.6, 124.1, 118.5, 117.1 (J = 25), 116.4, 103.2 (J = 22), 71.0, 65.3, 54.0, 52.6, 49.8, 15.8, 12.4 ppm. LRMS (ESI+) 680.0 $(M+H)^+$ HRMS (ESI+) m/z calcd. for $C_{25}H_{25}BrCl_2FN_3O_5S_2$ $(M+H)^+$ 679.9780, found 679.9850.

***N*-(5-bromo-4-fluoro-2-methylphenyl)-4-(3,5-dichlorophenylsulfonamido)-2-(piperazin-1-ylmethyl)benzenesulfonamide (MDL-812).** *N*-(5-bromo-4-fluoro-2-methylphenyl)-2-(chloromethyl)-4-(3,5-dichlorophenylsulfonamido)benzenesulfonamide (**8**, 100 mg, 0.162 mmol) was dissolved in 5 mL of dried THF, and then tert-butyl piperazine-1-carboxylate (60 mg, 0.324 mmol) and TEA (41 mg, 0.405 mmol) was added. The reaction was stirred at 50 °C for about 6 h. After concentration under reduced pressure, the residue was partitioned between ethyl acetate and water. The organic layer was further washed with saturated aq $NaHCO_3$, brine, dried over sodium sulfate, evaporated in vacuo, then the crude compound was dissolved in 3 mL of DCM and TFA (3 mL) was added at room temperature. The reaction was stirred at the same temperature for another 2 h. Then the solvent was almost moved out and subsequently purified by high performance liquid chromatography to afford **MDL-812** as a white solid (51.6

mg, 48%). ^{1}H NMR (DMSO-d$_6$, 400 MHz) δ 7.68-7.65 (m, 3H), 7.40-7.38 (d, *J* = 8.0 Hz, 1H), 7.27 (s, 1H), 7.15-7.13 (d, *J* = 8.0 Hz, 1H), 6.97-6.95 (d, *J* = 8.0 Hz, 1H), 6.90-6.89 (d, *J* = 4.0 Hz, 1H), 3.72 (s, 2H), 3.03-3.00 (m, 4H), 2.56-2.55 (m, 4H), 2.03 (s, 3H). ^{13}C NMR (DMSO-d$_6$ 100 MHz) 172.1, 157.8, 155.8, 150.7, 148.6, 138.0 (*J* = 6), 137.2, 134.9, 133.6, 131.5, 130.7 (*J* = 23), 128.7, 122.5, 118.7, 118.4 (*J* = 18), 104.4 (*J* = 18), 59.0, 51.0, 44.3, 17.7 ppm. LRMS (ESI+) 665.0 $(M+H)^+$ HRMS (ESI+) m/z calcd. for $C_{24}H_{24}BrCl_2FN_4O_4S_2$ $(M+H)^+$ 664.9783, found 664.9858.

***N*-(5-bromo-4-fluoro-2-methylphenyl)-2-((butyl(ethyl)amino)methyl)-4-(3,5-dichlorophenylsulfonamido)benzenesulfonamide (MDL-813).**

N-(5-bromo-4-fluoro-2-methylphenyl)-2-(chloromethyl)-4-(3,5-dichlorophenylsulfonamido)benzenesulfonamide (**8**, 100 mg, 0.162 mmol) was dissolved in 5 mL of dried THF, and then N-ethylbutan-1-amine (33 mg, 0.324 mmol) and TEA (41 mg, 0.405 mmol) was added. The reaction was stirred at 50 °C for about 6 h. After concentration under reduced pressure, the residue was partitioned between ethyl acetate and water. The organic layer was further washed with saturated aq $NaHCO_3$, brine, dried over sodium sulfate, evaporated in vacuo, and subsequently purified by high performance liquid chromatography to afford **MDL-813** as a white solid (56 mg, 51%). ^{1}H NMR (DMSO-d$_6$, 400 MHz) δ 7.98 (s, 1H), 7.75-7.74 (d, *J* = 4.0, 2H), 7.60-7.58 (m, 2H), 7.19-7.11 (m, 2H), 6.95-6.93(d, J = 8.0, 1H) 3.78(s, 2H), 2.47-2.42 (m,4H), 1.87 (s, 3H), 1.34-1.30 (m, 2H), 1.23-1.18 (m, 2H), 0.97-0.93 (m, 3H), 0.84-0.80 (t, 3H). ^{13}C NMR (DMSO-d$_6$ 100 MHz) 157.9, 155.4, 137.6, 137.5, 135.7, 133.4 (*J* = 3), 133.2, 131.2, 131.0, 125.6, 125.6, 118.7, 118.4 (*J* = 9), 104.8, 104.6, 99.9, 54.8, 52.9, 47.8, 20.4, 19.3, 17.6, 14.3, 11.4 ppm. LRMS (ESI+) 680.02 $(M+H)^+$ HRMS (ESI+) m/z calcd. for $C_{26}H_{29}BrCl_2FN_3O_4S_2$ $(M+H)^+$ 680.0144, found 680.0216.

2-([1,4'-bipiperidin]-1'-ylmethyl)-*N*-(5-bromo-4-fluoro-2-methylphenyl)-4-(3,5-dichlorophenylsulfonamido)benzenesulfonamide (MDL-814).

N-(5-bromo-4-fluoro-2-methylphenyl)-2-(chloromethyl)-4-(3,5-dichlorophenylsulfonamido)benzenesulfonamide (**8**, 100 mg, 0.162 mmol)was dissolved in 5 mL of dried THF, and then 1,4'-bipiperidine (55 mg, 0.324 mmol) and TEA (41 mg, 0.405 mmol) was added. The reaction was stirred at 50 °C for about 6 h. After concentration under reduced pressure, the residue was partitioned between ethyl acetate and water. The organic layer was further washed with saturated aq $NaHCO_3$, brine, dried over sodium sulfate, evaporated in vacuo, and subsequently purified by high performance liquid chromatography to afford **MDL-814** as a white solid (55 mg, 46%). ^{1}H NMR (DMSO-d$_6$, 400 MHz) δ 7.72-7.71 (t, 1H), 7.64-7.63 (d, 2H), 7.23-7.22 (d, *J* = 4.0 Hz, 2H), 7.20 (s, 1H), 6.76-6.73 (m, 1H), 6.69-6.67 (d, *J* = 8.0 Hz, 1H), 3.64 (s, 2H), 2.84-2.81 (m, 6H), 2.03 (s, 3H), 1.87-1.85 (d, *J* = 8.0 Hz, 2H), 1.62-1.58 (m, 4H), 1.44-1.38 (m, 4H). ^{13}C NMR (DMSO-d$_6$ 100 MHz) 157.4, 154.9, 148.6, 137.9 (*J* = 8), 136.2, 134.1, 133.1, 130.6, 130.2, 129.6, 124.7, 122.1, 118.2, 117.9, 103.9, 103.7, 61.8, 58.9, 51.9, 49.4, 26.6, 24.1, 22.6, 17.3 ppm. LRMS (ESI+) 747.02 $(M+H)^+$ HRMS (ESI+) m/z calcd. for $C_{30}H_{34}BrCl_2FN_4O_4S_2$ $(M+H)^+$ 747.0637.

***N*-(5-bromo-4-fluoro-2-methylphenyl)-4-(3,5-dichlorophenylsulfonamido)-2-((4-(3-methoxypropyl)piperazin-1-yl)methyl)benzenesulfonamide (MDL-815).**

N-(5-bromo-4-fluoro-2-methylphenyl)-2-(chloromethyl)-4-(3,5-dichlorophenylsulfon amido)benzenesulfonamide (**8**, 100 mg, 0.162 mmol)was dissolved in 5 mL of dried THF, and then 1-(3-methoxypropyl)piperazine (51 mg, 0.324 mmol) and TEA (41 mg, 0.405 mmol) was added. The reaction was stirred at 50 °C for about 6 h. After concentration under reduced pressure, the residue was partitioned between ethyl acetate and water. The organic layer was further washed with saturated aq $NaHCO_3$, brine, dried over sodium sulfate, evaporated in vacuo, and subsequently purified by high performance liquid chromatography to afford **MDL-815** as a white solid (57 mg, 48%). ^{1}H NMR (DMSO-d_6, 400 MHz) δ7.79 (s, 1H), 7.68 (s, 2H), 7.30-7.21 (m, 3H), 6.86-6.84 (d, *J* = 8.0, 1H), 6.68-6.66 (d, *J* = 8.0, 1H), 3.67 (s, 2H), 3.32-3.30 (4, 2H), 3.2 (s, 3H), 2.39-2.31(m, 8H), 2.01 (s, 3H), 1.65-1.62(m, 2H). ^{13}C NMR (DMSO-d_6 100 MHz) ^{13}C NMR (100 MHz, DMSO) δ 157.6, 155.1, 146.9, 138.1 (*J* = 7), 136.5, 134.4, 132.7, 130.9, 130.5, 130.3, 127.6, 124.8, 121.9, 118.2, 117.9, 103.9 (*J* = 22), 69.9, 59.0, 57.8, 54.4, 52.4, 52.3, 26.2, 17.3 ppm. LRMS (ESI+) 737.04 $(M+H)^+$ HRMS (ESI+) m/z calcd. for $C_{28}H_{32}BrCl_2FN_4O_5S_2$ $(M+H)^+$ 737.0427.

***N*-(5-bromo-4-fluoro-2-methylphenyl)-2-((4-butylpiperazin-1-yl)methyl)-4-(3,5-dichlorophenylsulfonamido)benzenesulfonamide (MDL-816).**
N-(5-bromo-4-fluoro-2-methylphenyl)-2-(chloromethyl)-4-(3,5-dichlorophenylsulfon amido) benzenesulfonamide (**8**, 100 mg, 0.162 mmol) was dissolved in 5 mL of dried THF, and then 1-butylpiperazine (46 mg, 0.324 mmol) and TEA (41 mg, 0.405 mmol) was added. The reaction was stirred at 50 °C for about 6 h. After concentration under reduced pressure, the residue was partitioned between ethyl acetate and water. The organic layer was further washed with saturated aq $NaHCO_3$, brine, dried over sodium sulfate, evaporated in vacuo, and subsequently purified by high performance liquid chromatography to afford **MDL-816** as a white solid (50 mg, 43%). ^{1}H NMR (DMSO-d_6, 400 MHz) δ 7.87-7.86 (t, 1H), 7.73-7.72 (d, 2H), 7.40-7.36 (m, 2H), 7.19-7.17 (d, *J* = 8.0 Hz, 1H), 6.98-6.95 (m, 1H), 6.84-6.82 (d, *J* = 8.0 Hz, 1H), 3.69 (s, 2H), 2.70-2.52 (m, 4H), 2.51-2.44 (m, 4H), 1.90 (s, 1H), 1.47-1.41 (m, 2H), 1.30-1.20 (m, 3H), 0.93-0.83 (m, 4H). ^{13}C NMR (DMSO-d_6 100 MHz) 164.4, 155.8, 145.5, 138.3 (*J* = 5), 137.8, 135.3, 132.9 (*J* = 3), 131.9 (*J* = 22), 131.1, 125.6, 121.5, 118.7, 118.5, 118.3, 104.6 (*J* = 23), 100.0, 58.7, 57.1, 52.6, 51.9, 29.5, 27.8, 20.3, 19.3, 17.7, 14.2 ppm. LRMS (ESI+) 721.1 $(M+H)^+$ HRMS (ESI+) m/z calcd. for $C_{28}H_{32}BrCl_2FN_4O_4S_2$ $(M+H)^+$ 721.0482.

***N*-(5-bromo-4-fluoro-2-methylphenyl)-4-(3,5-dichlorophenylsulfonamido)-2-((3-(dimethylamino) pyrrolidin-1-yl) methyl) benzenesulfonamide (MDL-817).**
N-(5-bromo-4-fluoro-2-methylphenyl)-2-(chloromethyl)-4-(3,5-dichlorophenylsulfon amido) benzenesulfonamide (**8**, 100 mg, 0.162 mmol) was dissolved in 5 mL of dried THF, and then *N*,*N*-dimethylpyrrolidin-3-amine (37 mg, 0.324 mmol) and TEA (41 mg, 0.405 mmol) was added. The reaction was stirred at 50 °C for about 6 h. After concentration under reduced pressure, the residue was partitioned between ethyl acetate and water. The organic layer was further washed with saturated aq $NaHCO_3$, brine, dried over sodium sulfate, evaporated in vacuo, and subsequently purified by high performance liquid chromatography to afford **MDL-817** as a white solid (53 mg, 48%). ^{1}H NMR (DMSO-d_6, 400 MHz) δ 7.81-7.80 (t, 1H), 7.69-7.68 (d, 2H),

7.36-7.34 (d, J = 8.0, 1H), 7.24-7.17 (m, 2H), 6.92-6.87 (m, 2H), 3.82-3.71 (m, 2H), 3.29-3.19 (m, 1H), 2.69-2.59 (m, 2H), 2.55-2.52 (m, 1H), 2.42-2.36 (m, 7H), 2.01-1.93 (m, 4H), 1.79-1.74 (m, 1H). ^{13}C NMR (DMSO-d$_6$ 100 MHz) 157.4, 154.9, 146.7, 137.7, 137.4 (J = 8), 134.4, 132.7, 131.0, 130.7, 130.3, 127.9, 124.8, 120.9, 117.9 (J = 17), 117.7, 104.9 (J = 22), 64.3, 56.0, 55.9, 52.2, 41.7, 26.9, 17.1 ppm. LRMS (ESI+) 693.06 $(M+H)^+$ HRMS (ESI+) m/z calcd. for $C_{26}H_{28}BrCl_2FN_4O_4S_2$ $(M+H)^+$ 693.0167.

***N*-(5-bromo-4-fluoro-2-methylphenyl)-4-(3,5-dichlorophenylsulfonamido)-2-((dimethylamino)methyl)benzenesulfonamide (MDL-818).** *N*-(5-bromo-4-fluoro-2-methylphenyl)-2-(chloromethyl)-4-(3,5-dichlorophenylsulfonamido) benzenesulfonamide (**8**, 100 mg, 0.162 mmol) was dissolved in 5 mL dried THF, and then dimethylamine absolute in tetrahydrofuran solution (2 M, 0.245 mL) was added. The reaction was stirred at 50 °C for about 6 h. After concentration under reduced pressure, the residue was partitioned between ethyl acetate and water. The organic layer was further washed with saturated aq $NaHCO_3$, brine, dried over sodium sulfate, evaporated in vacuo, and subsequently purified by column chromatography on silica gel (petroleum ether: ethyl acetate = 2:1) to afford **MDL-818** as a white solid (66 mg, 65%). ^{1}H NMR (DMSO-d_6, 500 MHz) δ 7.92 (s, 1H), 7.76-7.75 (d, 2H), 7.49-7.48 (d, J = 5.0, 1H), 7.33 (s, 1H), 7.20-7.18 (d, J = 10.0, 1H), 7.10-7.07 (m, 1H), 6.91-6.89 (d, J = 10.0, 1H) 3.84 (s, 2H), 2.31 (s, 6H), 1.90 (s, 3H). ^{13}C NMR (DMSO-d_6, 125 MHz) 156.1 (J = 242.5), 136.96 (J = 9), 134.91, 132.96, 132.08, 130.57, 130.42, 129.55, 125.08, 122.09, 118.55, 118.03 (J = 22.5), 104.02 (J = 22.5), 59.62, 43.95, 17.09 ppm. LRMS (ESI+) 624.0 $(M+H)^+$, HRMS (ESI+) m/z calcd. for $C_{22}H_{22}BrCl_2FN_3O_4S_2^+$$(M+H)^+$623.9591, found 623.9585.

N-(5-bromo-4-fluoro-2-methylphenyl)-4-(3,5-dichlorophenylsulfonamido)-2-((ethyl(methyl)amino)methyl)benzenesulfonamide (MDL-819). *N*-(5-bromo-4-fluoro-2-methylphenyl)-2-(chloromethyl)-4-(3,5-dichlorophenylsulfonamido) benzenesulfonamide (**8**, 100 mg, 0.162 mmol) was dissolved in 5 mL of dried THF, and then N-methylethanamine (29 mg, 0.486 mmol) was added. The reaction was stirred at 50 ° C for about 6 h. After concentration under reduced pressure, the residue was partitioned between ethyl acetate and water. The organic layer was further washed with saturated aq $NaHCO_3$, brine, dried over sodium sulfate, evaporated in vacuo, and subsequently purified by column chromatography on silica gel (petroleum ether: ethyl acetate = 2:1) to afford **MDL-819** as a white solid (78 mg, 76%). ^{1}H NMR (DMSO-d_6, 500 MHz) δ7.65-7.62 (m, 3H), 7.23-7.21 (m, 2H), 7.00-6.99 (d, J = 5.0, 1H), 6.74-6.72 (m, 1H), 6.67-6.65 (d, J = 10.0, 1H), 3.70 (s, 2H), 2.47-2.43 (m, 2H), 2.11-2.10 (d, 6H), 0.98-0.95 (t, 3H). ^{13}C NMR (DMSO-d_6 125 MHz) 156.16(J = 241.3), 150.45, 137.53, 136.14, 134.45, 139.94 (J = 87.5), 129.41, 125.17, 124.16, 118.64, 118.44, 104.22 (J = 22.5), 60.06, 51.00, 41.04, 18.09, 11.87 ppm. LRMS (ESI+) 638.0 $(M+H)^+$, HRMS (ESI+) m/z calcd. for $C_{23}H_{24}BrCl_2FN_3O_4S_2^+$ $(M+H)^+$ 637.9747, found 637.9742.

***N*-(5-bromo-4-fluoro-2-methylphenyl)-4-(3,5-dichlorophenylsulfonamido)-2-((methyl(propyl)amino)methyl)benzenesulfonamide (MDL-820).** *N*-(5-bromo-4-fluoro-2-methylphenyl)-2-(chloromethyl)-4-(3,5-dichlorophenylsulfon

amido) benzenesulfonamide (**8**, 100 mg, 0.162 mmol) was dissolved in 5 mL of dried THF, and then N-methylpropan-1-amine (36 mg, 0.486 mmol) was added. The reaction was stirred at 50 °C for about 6 h. After concentration under reduced pressure, the residue was partitioned between dichloromethane and water. The organic layer was further washed with saturated aq $NaHCO_3$, brine, dried over sodium sulfate, evaporated in vacuo, and subsequently purified by column chromatography on silica gel(petroleum ether: ethyl acetate = 2:1) to afford **MDL-820** as a white solid (76 mg, 72%). ^{1}H NMR (DMSO-d_6, 500 MHz) δ7.96 (s, 1H), 7.76-7.75 (d, 2H), 7.55-7.53 (d, *J* = 10.0, 1H), 7.46 (s, 1H), 7.20-7.18 (d, *J* = 10.0, 1H), 7.13-7.11 (m, 1H), 6.90-6.89 (d, *J* = 5.0, 1H), 3.78 (s, 2H), 2.42-2.41 (m, 2H), 2.15 (s, 3H), 1.89 (s, 3H), 1.48-1.43 (m, 2H), 0.85-0.82 (t, 3H). ^{13}C NMR (DMSO-d_6 125 MHz) 156.74 (*J* = 242.5), 143.64, 137.81, 137.75, 135.58, 133.12 (*J* = 40), 131.25, 131.09, 125.66, 121.83, 118.64 (*J* = 22.5), 104.64 (*J* = 21.3), 59.30, 58.42, 41.60, 19.70, 17.62, 11.94 ppm. LRMS (ESI+) 651.9 $(M+H)^+$, HRMS (ESI+) m/z calcd. for $C_{24}H_{26}BrCl_2FN_3O_4S_2^+$ $(M+H)^+$ 651.9904, found 651.9904.

***N*-(5-bromo-4-fluoro-2-methylphenyl)-4-(3,5-dichlorophenylsulfonamido)-2-(piperidin-1-ylmethyl)benzenesulfonamide (MDL-821).**

N-(5-bromo-4-fluoro-2-methylphenyl)-2-(chloromethyl)-4-(3,5-dichlorophenylsulfonamido) benzenesulfonamide (**8**, 100 mg, 0.162 mmol) was dissolved in 5 mL of dried THF, and then piperidine (42 mg, 0.486 mmol) was added. The reaction was stirred at 50 °C for about 6 h. After concentration under reduced pressure, the residue was partitioned between ethyl acetate and water. The organic layer was further washed with saturated aq $NaHCO_3$, brine, dried over sodium sulfate, evaporated in vacuo, and subsequently purified by column chromatography on silica gel (petroleum ether: ethyl acetate = 2:1) to afford **MDL-821** as a white solid (69 mg, 64%). ^{1}H NMR (DMSO-d_6, 500 MHz) δ7.99 (s, 1H), 7.78-7.77 (d, 2H), 7.49-7.45 (m, 2H), 7.22-7.20 (d, *J* = 10.0, 1H), 7.11-7.09 (m, 1H), 6.71 (s, 1H), 3.79 (s, 2H), 2.44(s, 4H), 1.93 (s, 3H), 1.50-1.42 (m, 6H). ^{13}C NMR (DMSO-d_6 125 MHz) 156.98 (*J* = 243.8) 138.61 (*J* = 9.0), 135.63, 133.04, 131.51, 131.22, 125.70, 122.01, 118.75 (*J* = 22.5), 104.54 (*J* = 21.3), 59.63, 54.07, 25.40, 23.57, 17.71 ppm. LRMS (ESI+) 664.0 $(M+H)^+$, HRMS (ESI+) m/z calcd. for $C_{25}H_{26}BrCl_2FN_3O_4S_2^+$ $(M+H)^+$ 663.9904, found 663.9900.

N-(5-bromo-4-fluoro-2-methylphenyl)-4-(3,5-dichlorophenylsulfonamido)-2-((2-(hydroxymethyl)pyrrolidin-1-yl)methyl)benzenesulfonamide (MDL-822).

N-(5-bromo-4-fluoro-2-methylphenyl)-2-(chloromethyl)-4-(3,5-dichlorophenylsulfonamido) benzenesulfonamide (**8**, 100 mg, 0.162 mmol) was dissolved in 5 mL of dried THF, and then piperidine (49 mg, 0.486 mmol) was added. The reaction was stirred at 50 °C for about 6 h. After concentration under reduced pressure, the residue was partitioned between ethyl acetate and water. The organic layer was further washed with saturated aq $NaHCO_3$, brine, dried over sodium sulfate, evaporated in vacuo, and subsequently purified by column chromatography on silica gel (petroleum ether: ethyl acetate = 2:1) to afford **MDL-822** as a white solid (66 mg, 60%). ^{1}H NMR (DMSO-d_6, 500 MHz) δ7.98 (s, 1H), 7.77-7.76 (d, 2H), 7.50-7.48 (d, *J* = 10.0, 1H), 7.44 (s, 1H), 7.20-7.18 (d, *J* = 10.0, 1H), 7.08-7.06 (m, 1H), 6.94-6.93 (d, *J* = 5.0, 1H), 4.49-4.46 (d, *J* = 15.0, 1H), 3.62-3.60 (m, 2H), 3.49-3.43 (m, 3H), 2.75-2.60 (m, 2H), 2.18-2.13

(m, 1H), 1.90-1.88 (m, 4H), 1.72-1.57 (m, 2H). ^{13}C NMR (DMSO-d_6 125 MHz) 156.27 (J = 242.5), 137.79 (J = 9), 135.07, 132.51, 131.13, 130.72, 125.12, 121.41, 118.12 (J = 22.5), 104.96 (J = 22.5), 65.55, 62.20, 55.47, 53.76, 33.19, 27.13, 22.43, 17.12ppm. LRMS (ESI+) 680.0 $(M+H)^+$, HRMS (ESI+) m/z calcd. for $C_{25}H_{26}BrCl_2FN_3O_5S_2^+$ $(M+H)^+$ 679.9853, found 679.9841.

NMR spectra of compounds MDL analogs

N-(5-bromo-4-fluoro-2-methylphenyl)-4-(3,5-dichlorophenylsulfonamido)-2-(hydroxymethyl)benzenesulfonamide (7)

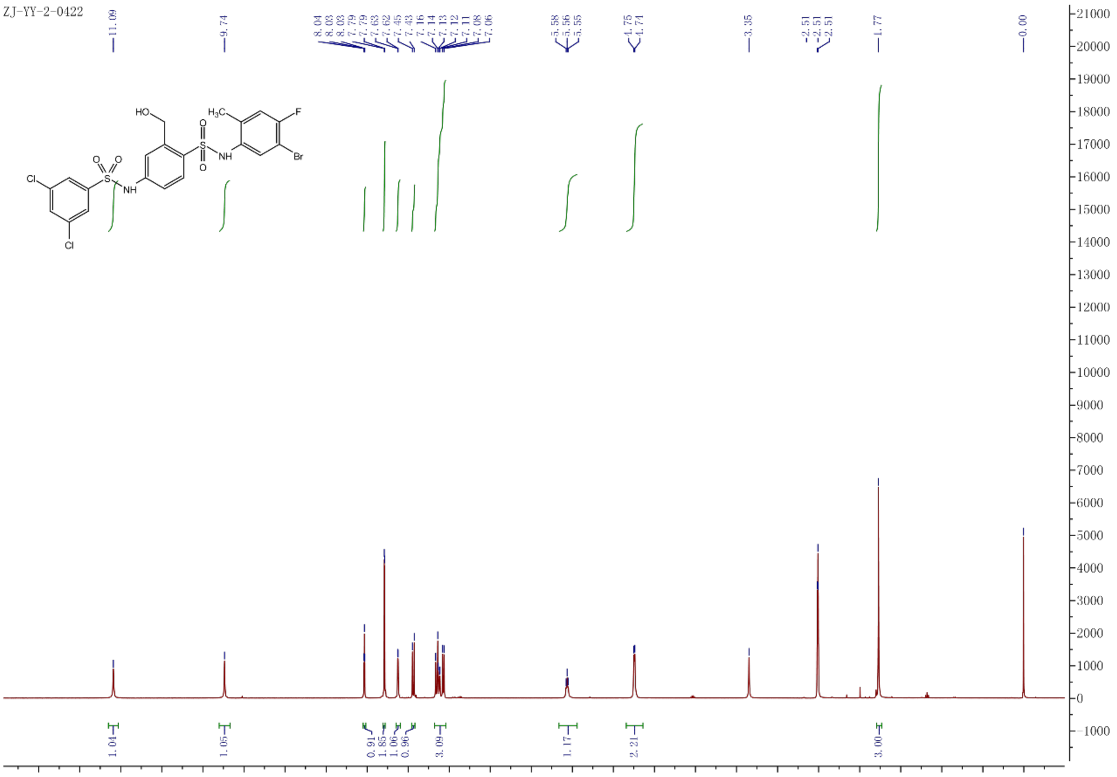

N-(5-bromo-4-fluoro-2-methylphenyl)-2-(chloromethyl)-4-(3,5-dichlorophenylsulfonamido)benzenesulfonamide (8)

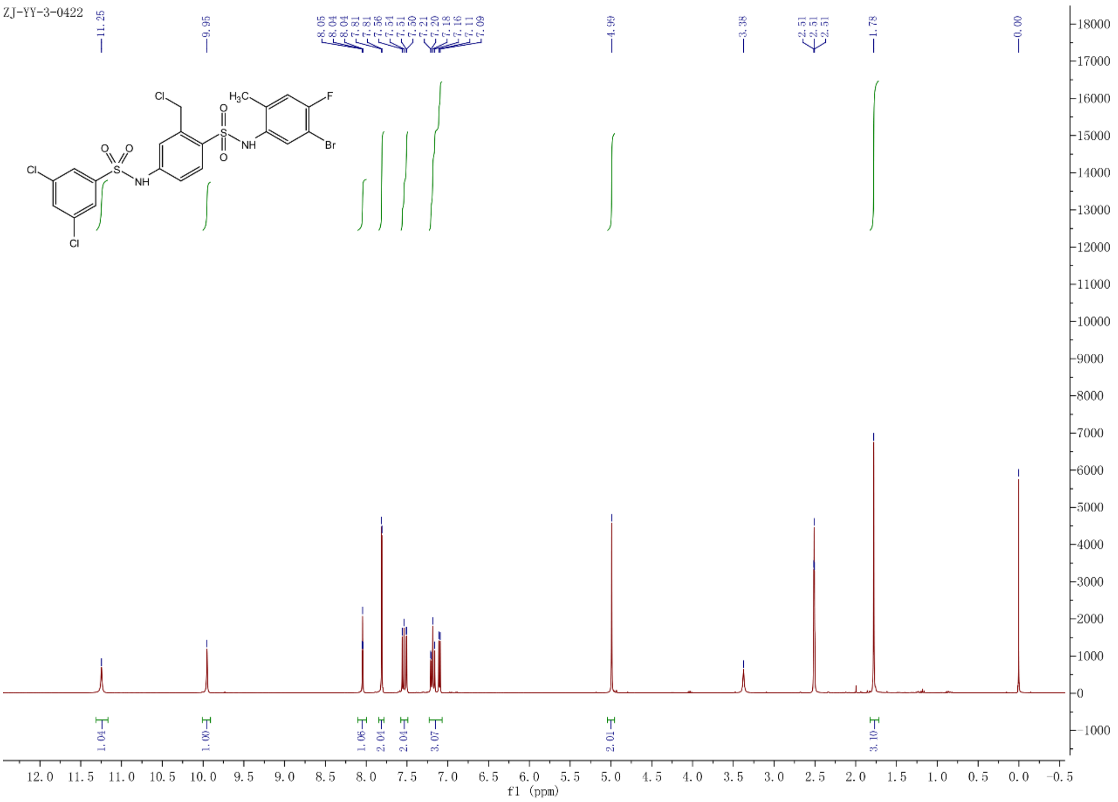

MDL-811

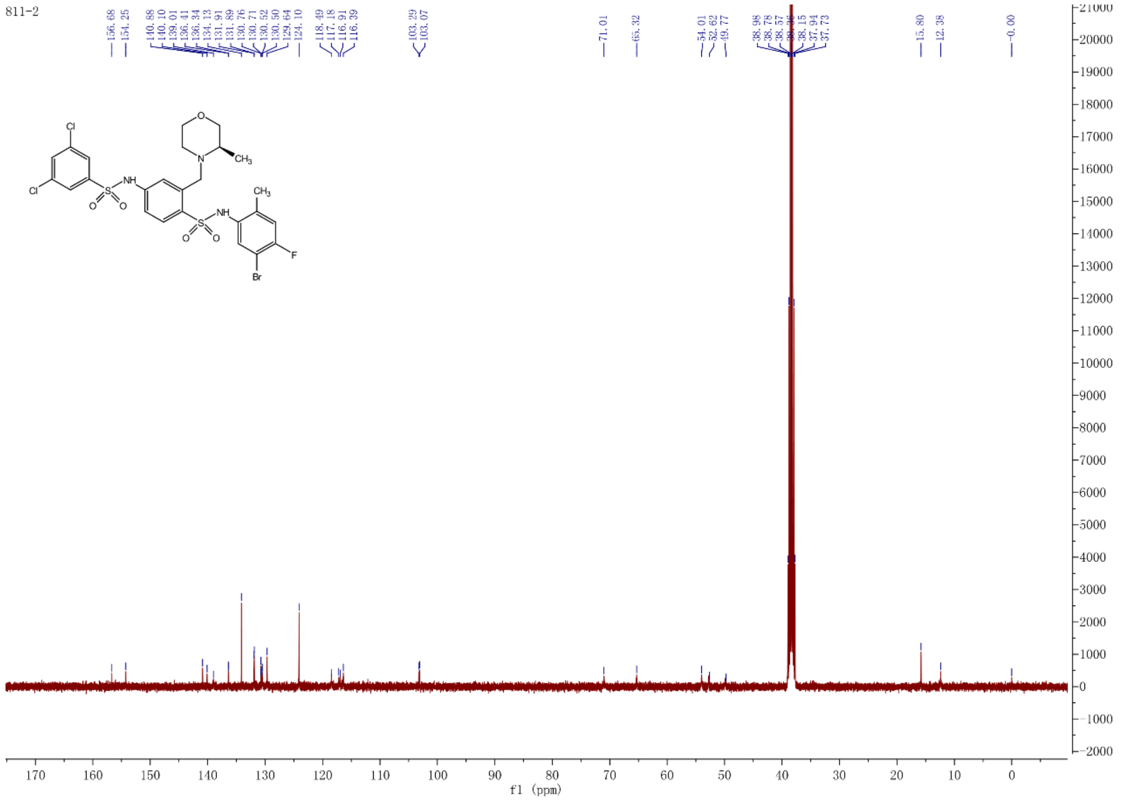

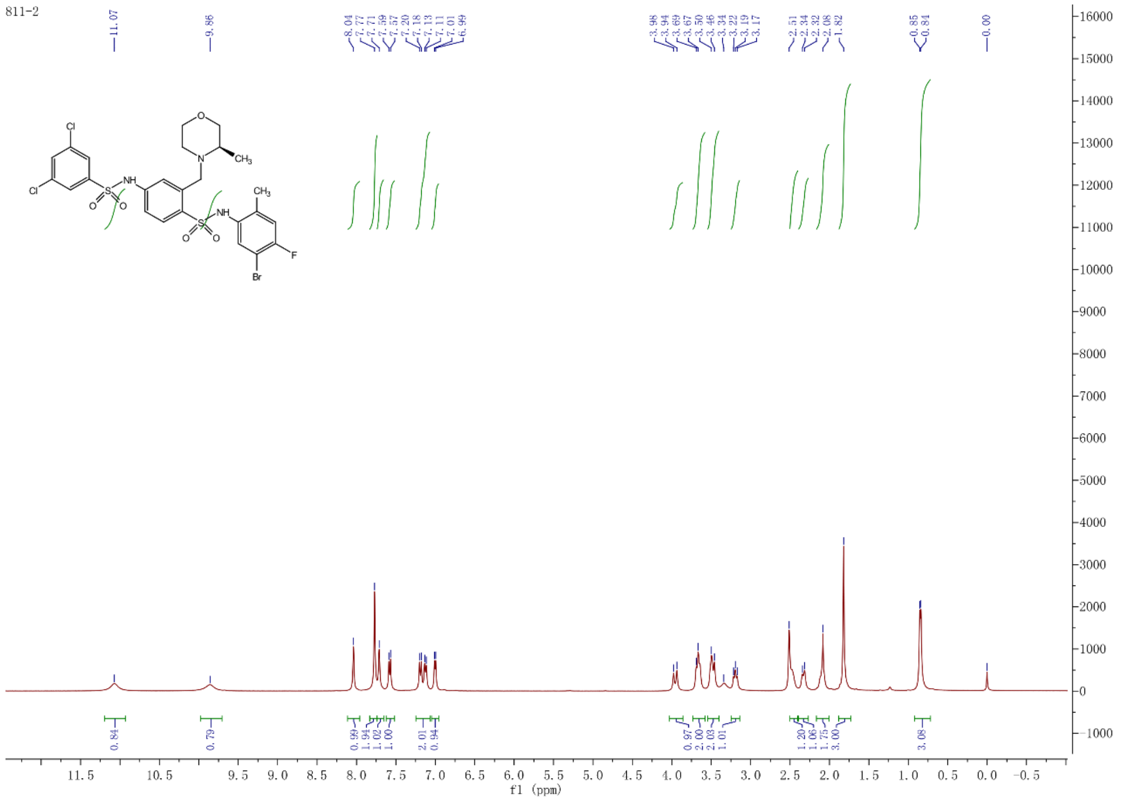

MDL-812

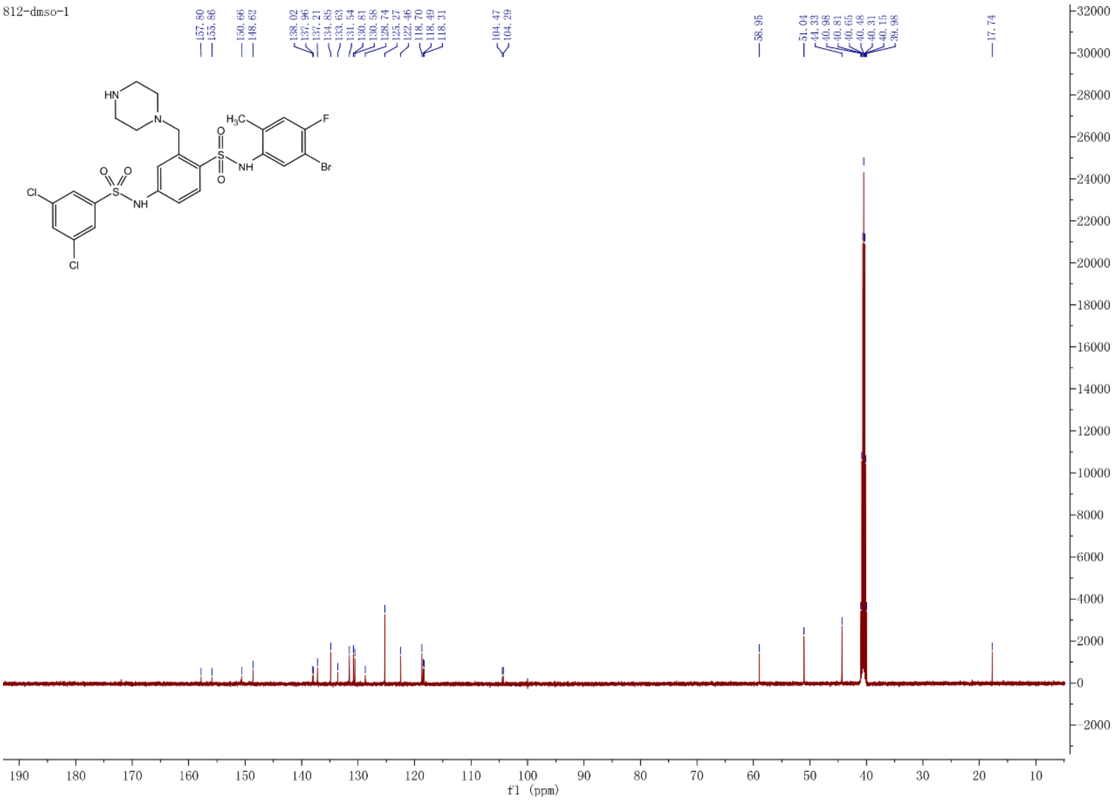
812-dmso-1
157.80
155.86
150.66
148.62
138.02
137.96
137.21
134.85
133.63
131.54
130.81
130.58
128.74
125.27
122.46
118.70
118.49
118.31
104.47
104.29
58.95
51.04
44.33
40.98
40.81
40.65
40.48
40.31
40.15
39.98
17.74
f1 (ppm)

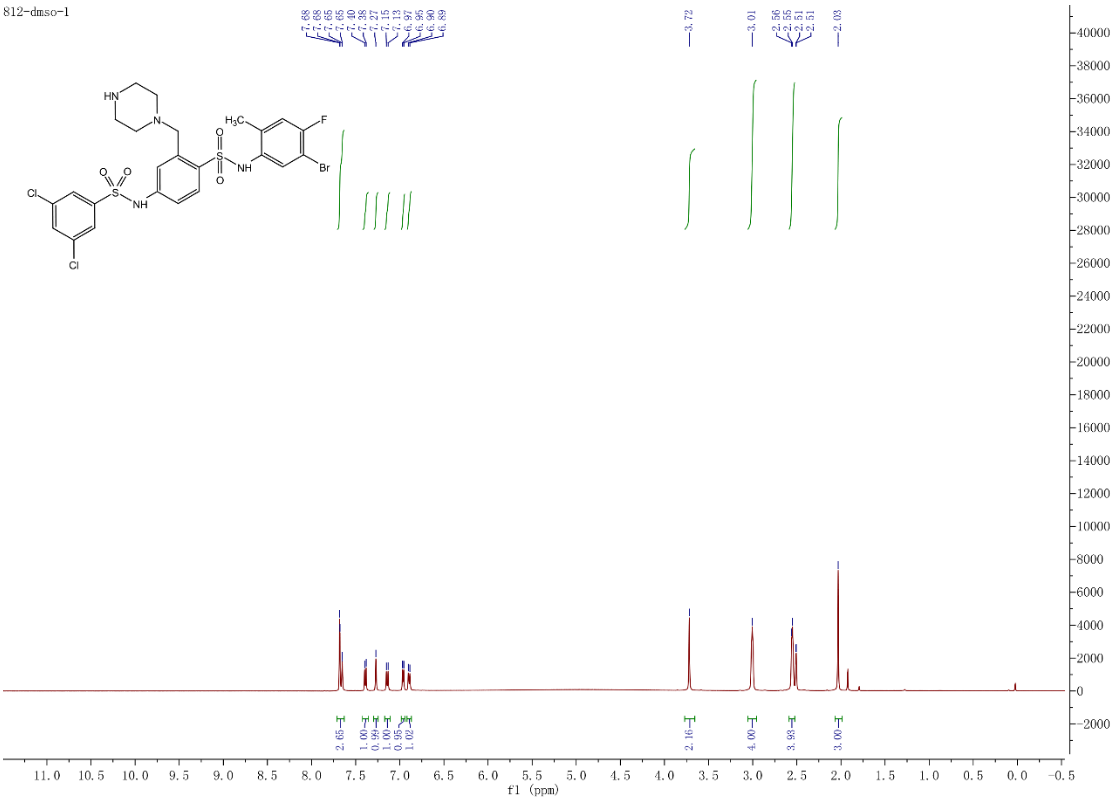
812-dmso-1
7.68
7.68
7.65
7.65
7.40
7.38
7.27
7.15
7.13
6.97
6.95
6.90
6.89
3.72
3.01
2.56
2.55
2.51
2.51
2.03
2.65
1.00
0.99
1.00
0.95
1.02
2.16
4.00
3.93
3.00
f1 (ppm)

MDL-813

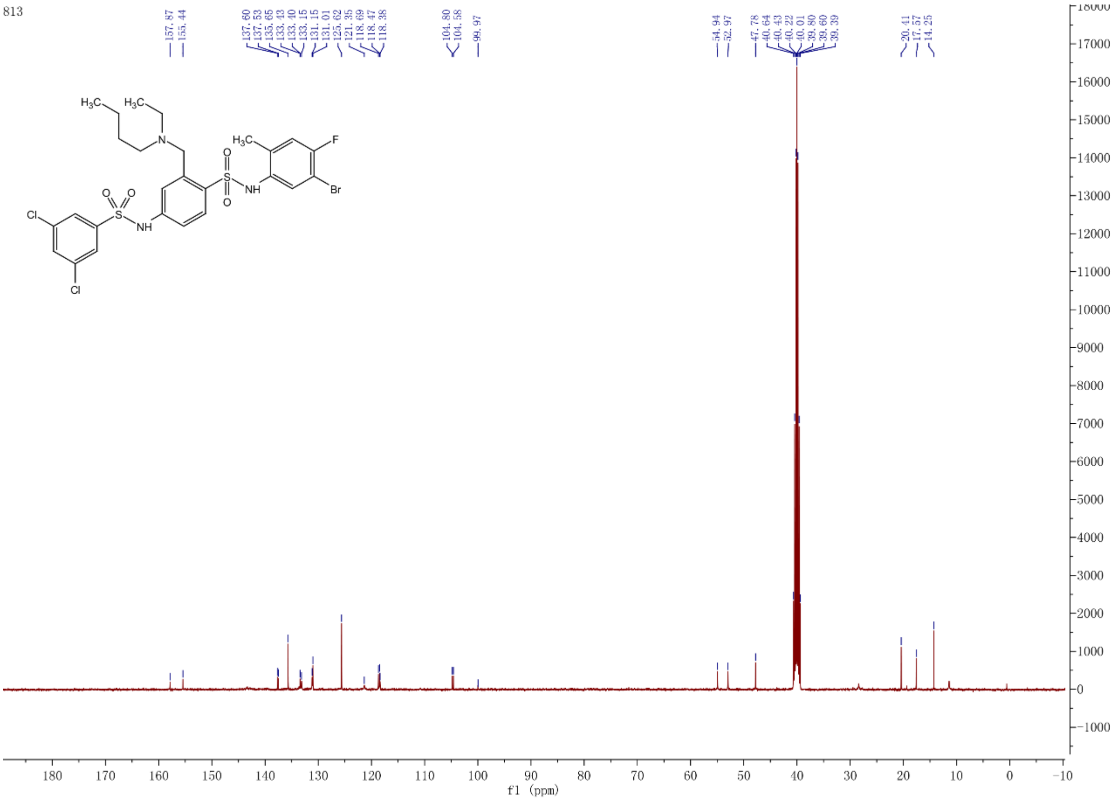

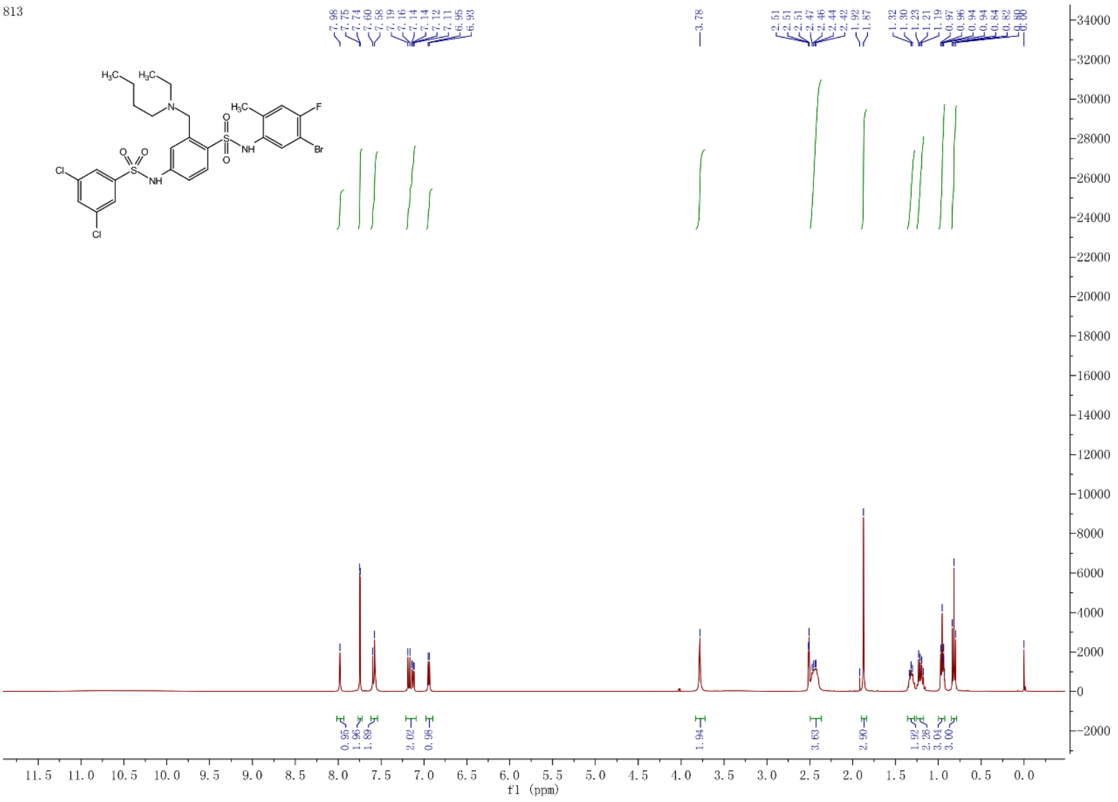

MDL-814

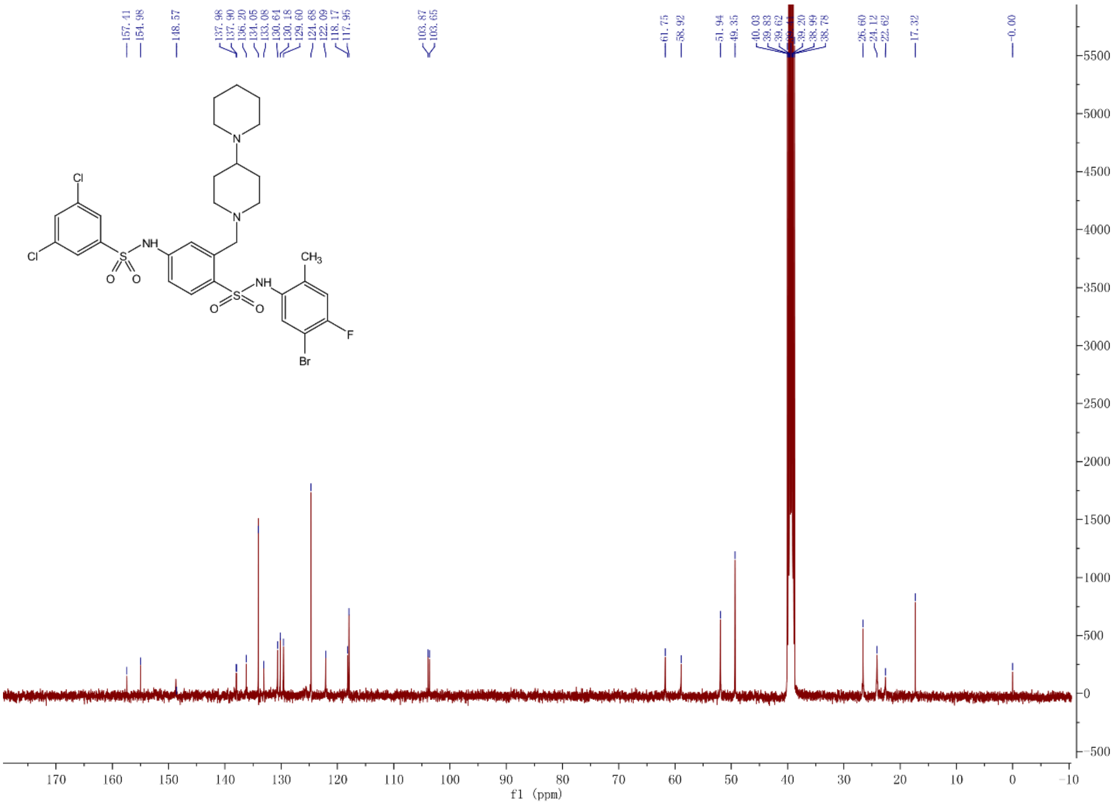
157.41
154.98
148.57
137.98
137.90
136.20
134.05
133.08
130.64
130.18
129.60
124.68
122.09
118.17
117.95
103.87
103.65
61.75
58.92
51.94
49.35
40.03
39.83
39.62
39.20
38.99
38.78
26.60
24.12
22.62
17.32
0.00
Cl
Cl
NH
S
O
O
N
N
CH3
NH
S
O
O
F
Br
f1 (ppm)

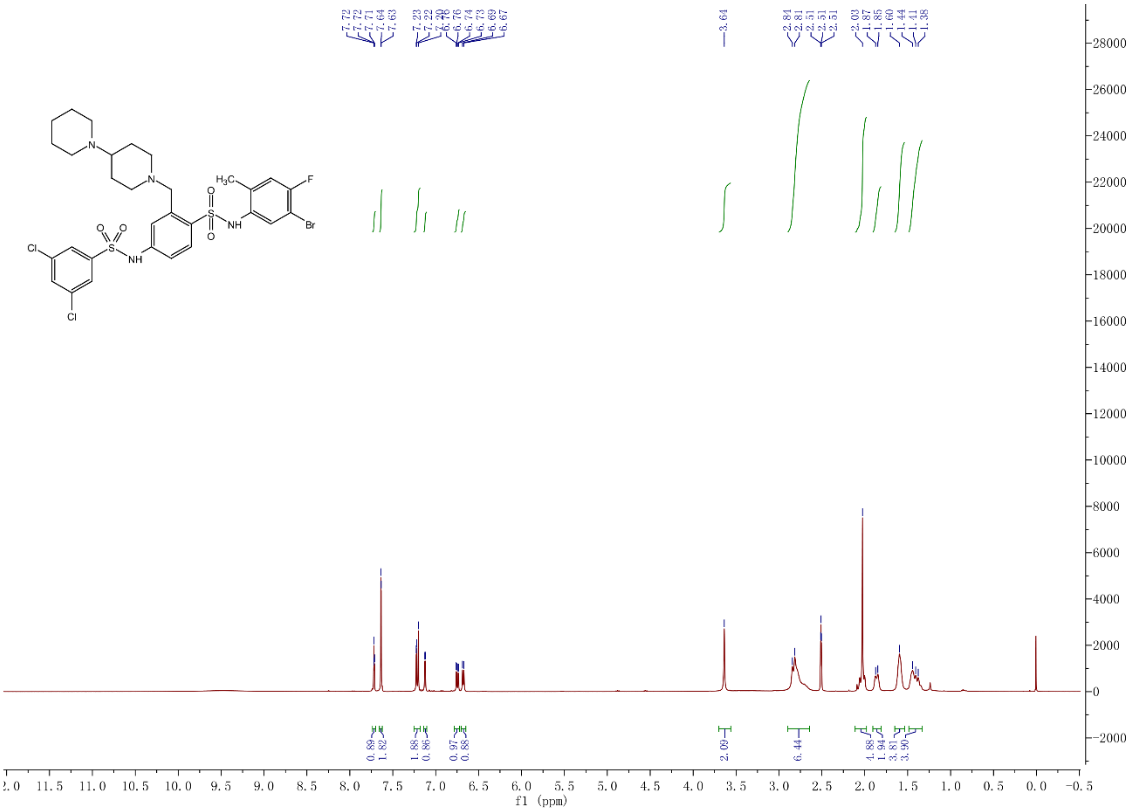
7.72
7.72
7.71
7.64
7.63
7.23
7.22
6.76
6.74
6.73
6.69
6.67
3.64
2.84
2.81
2.51
2.51
2.51
2.03
1.87
1.85
1.60
1.44
1.41
1.38
N
N
H3C
F
O
S
O
NH
Br
O
S
O
NH
Cl
Cl
0.89
1.82
1.88
0.86
0.97
0.88
2.09
6.44
4.88
1.94
3.81
3.90
f1 (ppm)

MDL-815

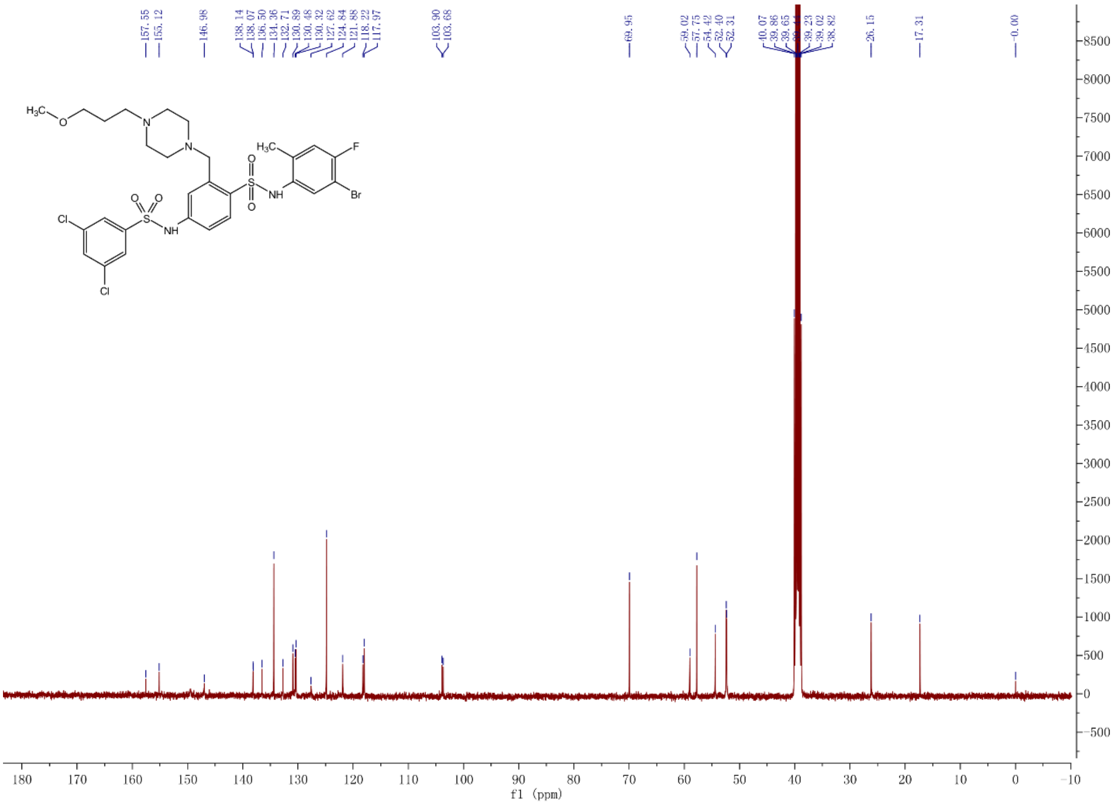
157.55
155.12
146.98
138.14
138.07
136.50
134.36
132.71
130.89
130.48
130.32
127.62
124.84
121.88
118.22
117.97
103.90
103.68
69.95
59.02
57.75
54.42
52.40
52.31
40.07
39.86
39.65
39.23
39.02
38.82
26.15
17.31
0.00
H_3C
O
N
N
O
S
O
NH
F
Br
Cl
Cl
f1 (ppm)

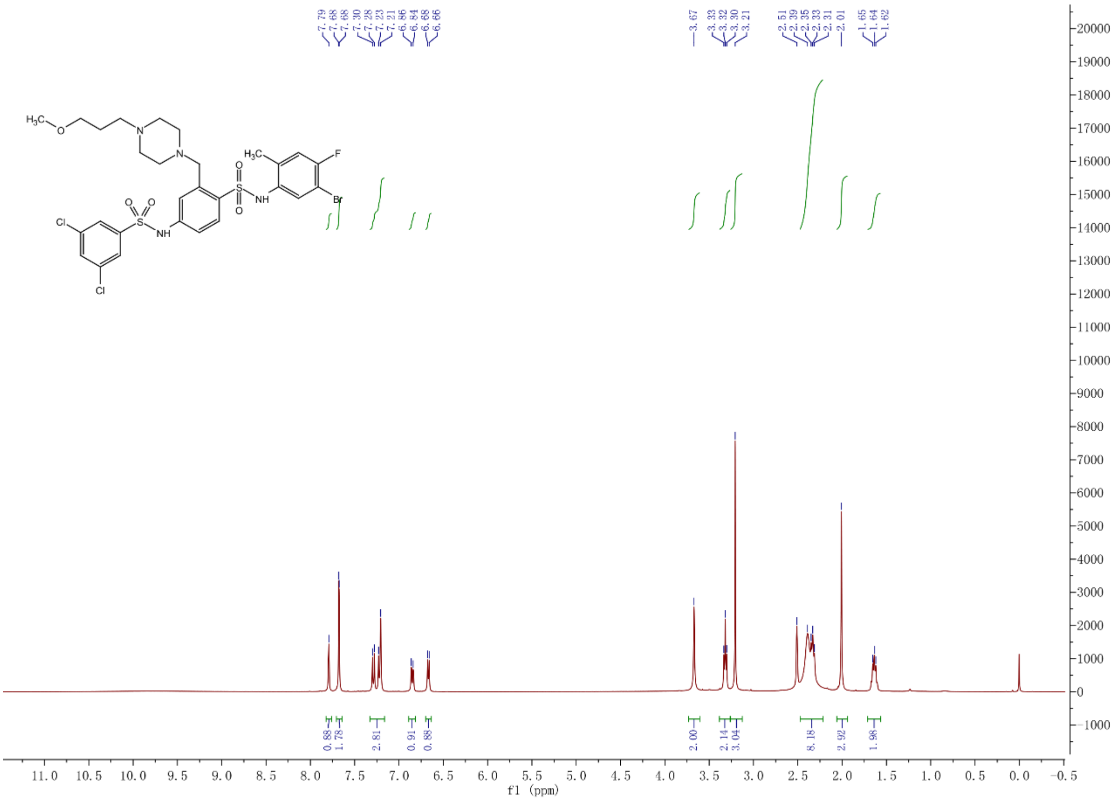
7.79
7.68
7.68
7.30
7.28
7.23
7.21
6.86
6.84
6.68
6.66
3.67
3.33
3.32
3.30
3.21
2.51
2.39
2.35
2.33
2.31
2.01
1.65
1.64
1.62
0.88
1.78
2.81
0.91
0.88
2.00
2.14
3.04
8.18
2.92
1.98
H_3C
F
Br
NH
Cl
Cl
f1 (ppm)

MDL-816

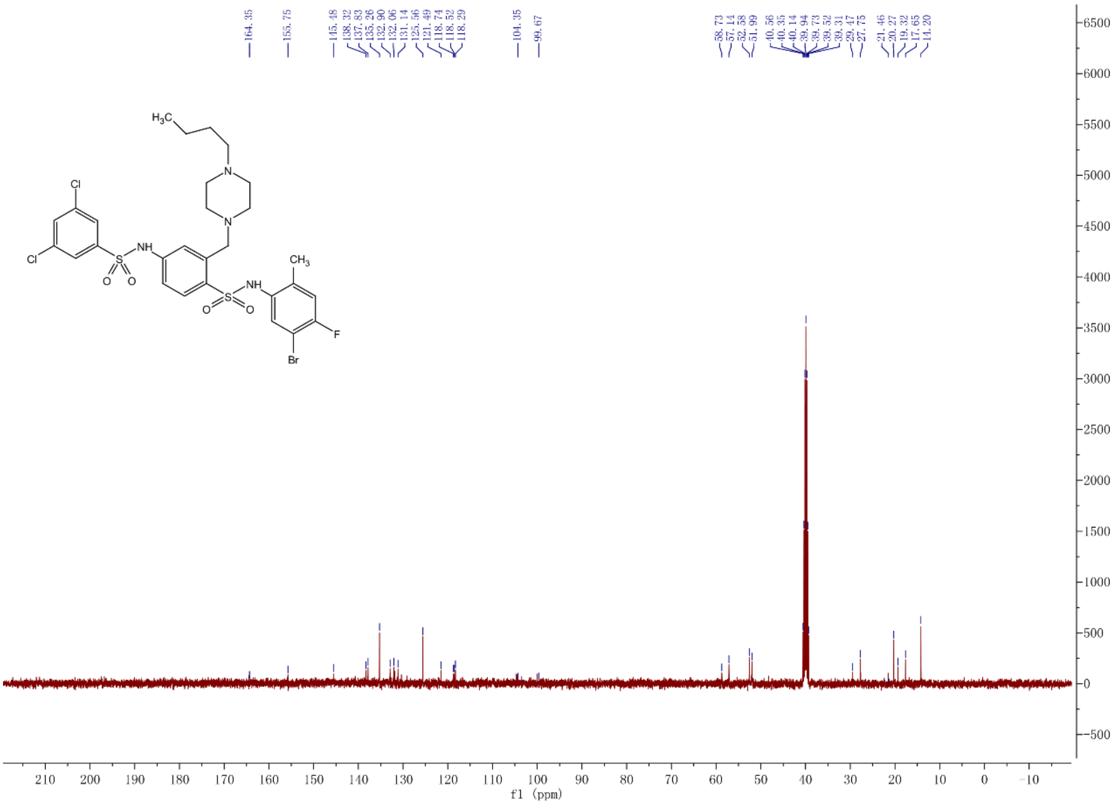

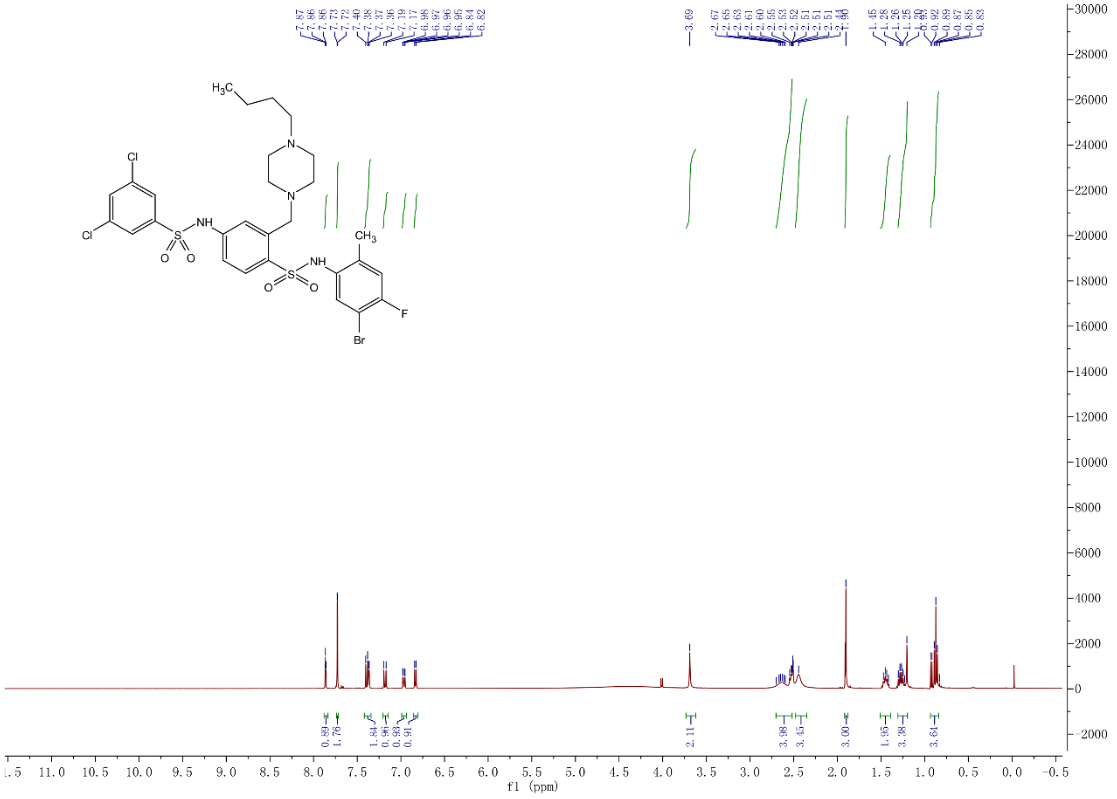

MDL-817

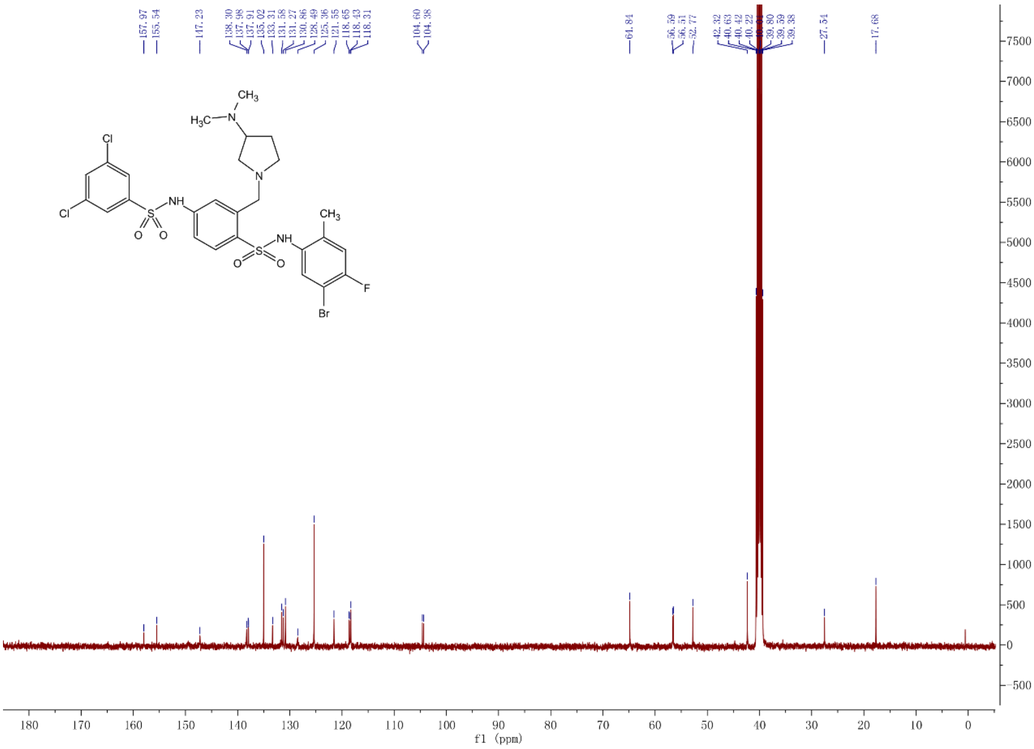

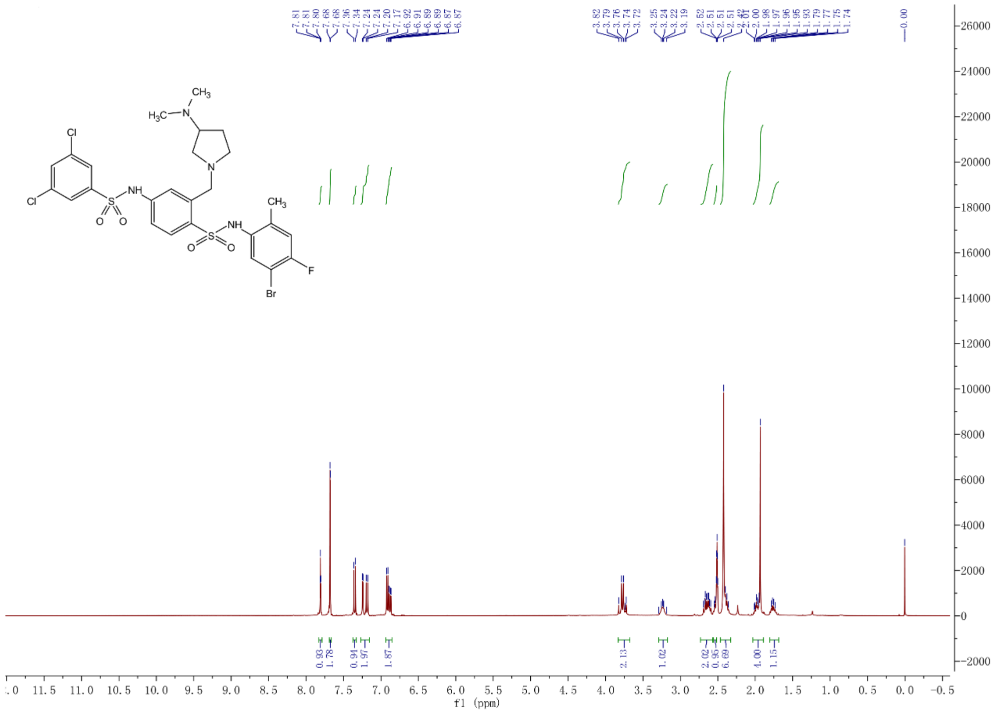

MDL-818

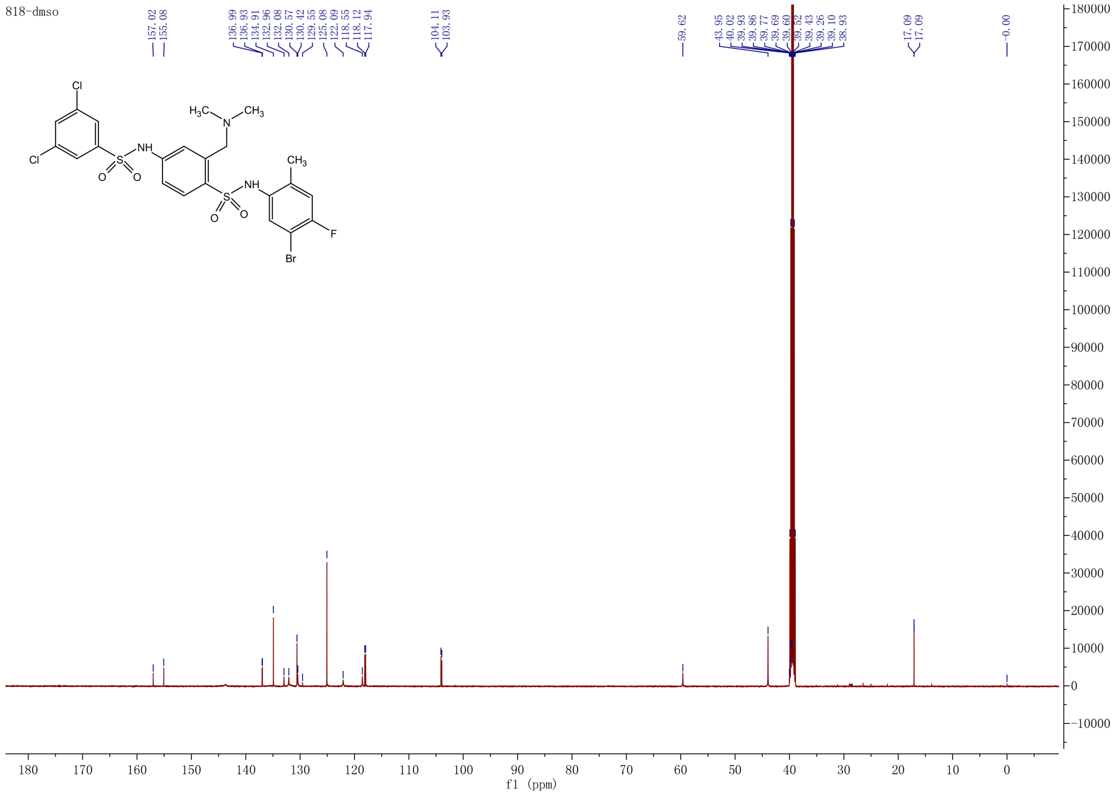
818-dmso
157.02
155.08
136.99
136.93
134.91
132.96
132.08
130.57
130.42
129.55
125.08
122.09
118.55
118.12
117.94
104.11
103.93
59.62
43.95
40.02
39.93
39.86
39.77
39.69
39.60
39.52
39.43
39.26
39.10
38.93
17.09
17.09
-0.00
f1 (ppm)

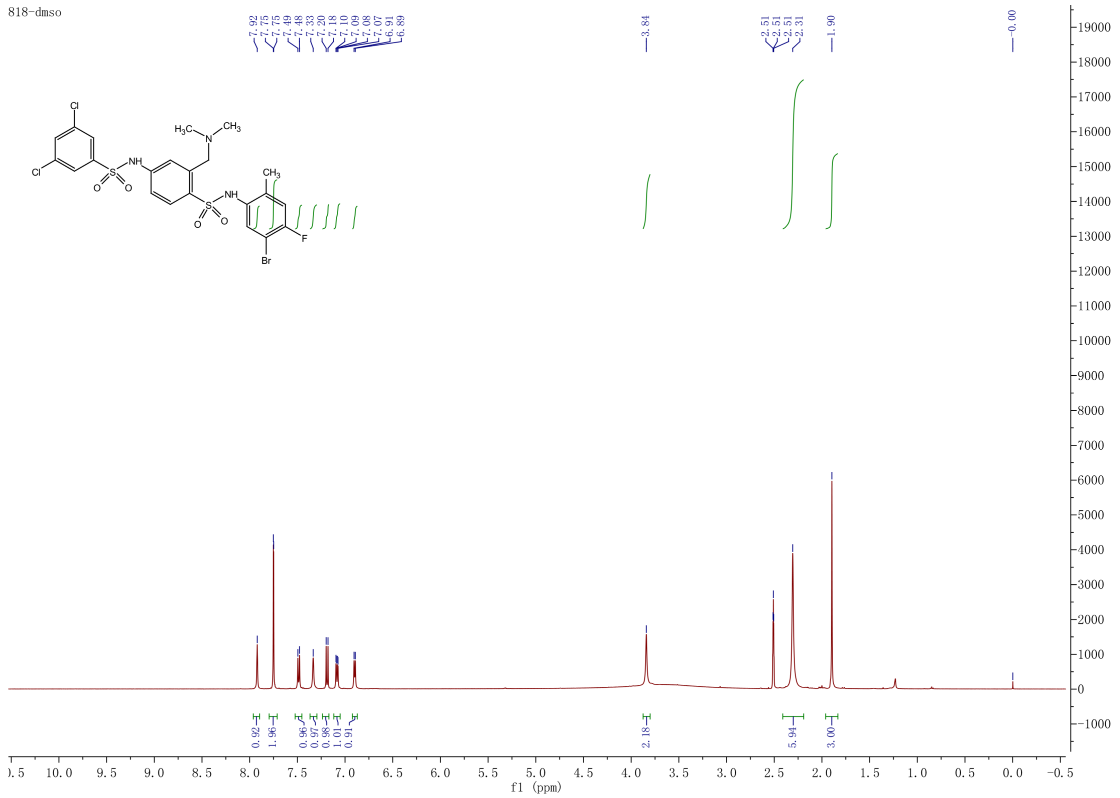
818-dmso
7.92
7.75
7.75
7.49
7.48
7.33
7.20
7.18
7.10
7.09
7.08
7.07
6.91
6.89
3.84
2.51
2.51
2.51
2.31
1.90
-0.00
0.92
1.96
0.96
0.97
0.98
1.01
0.91
2.18
5.94
3.00
f1 (ppm)

MDL-819

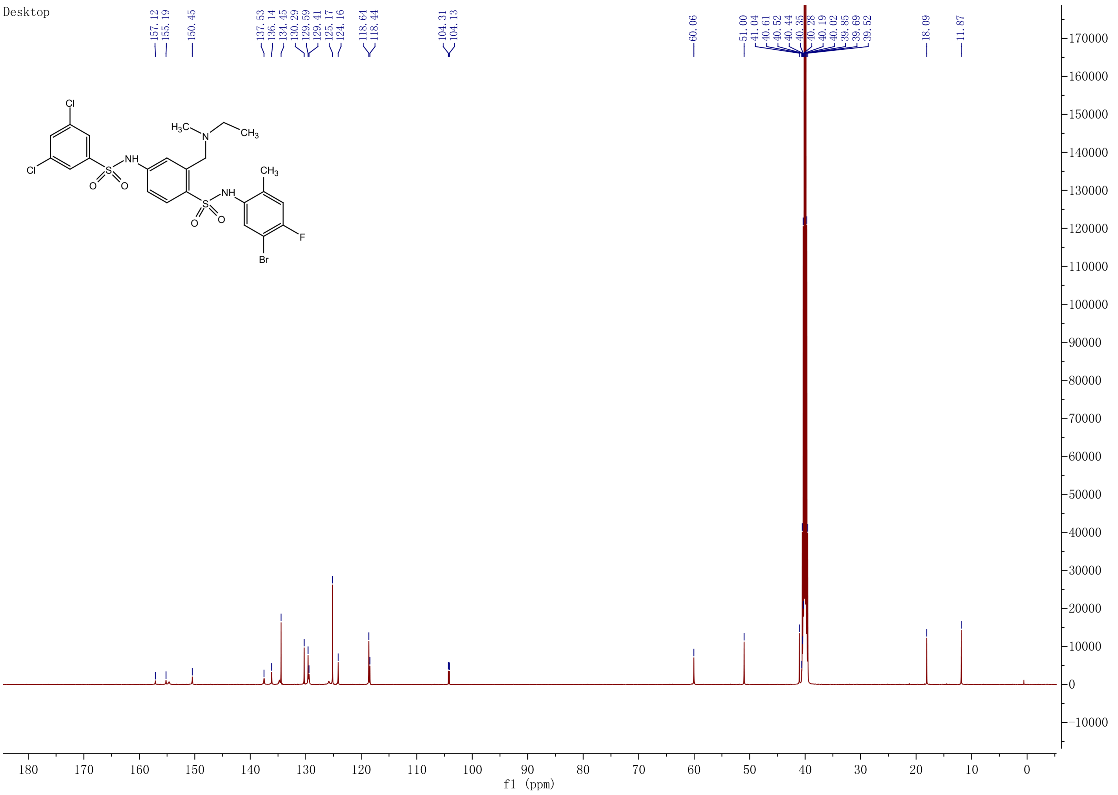
Desktop
157.12
155.19
150.45
137.53
136.14
134.45
130.29
129.59
129.41
125.17
124.16
118.64
118.44
104.31
104.13
60.06
51.00
41.04
40.61
40.52
40.44
40.35
40.28
40.19
40.02
39.85
39.69
39.52
18.09
11.87
Cl
Cl
NH
H3C
N
CH3
CH3
S
O
O
NH
F
Br
f1 (ppm)

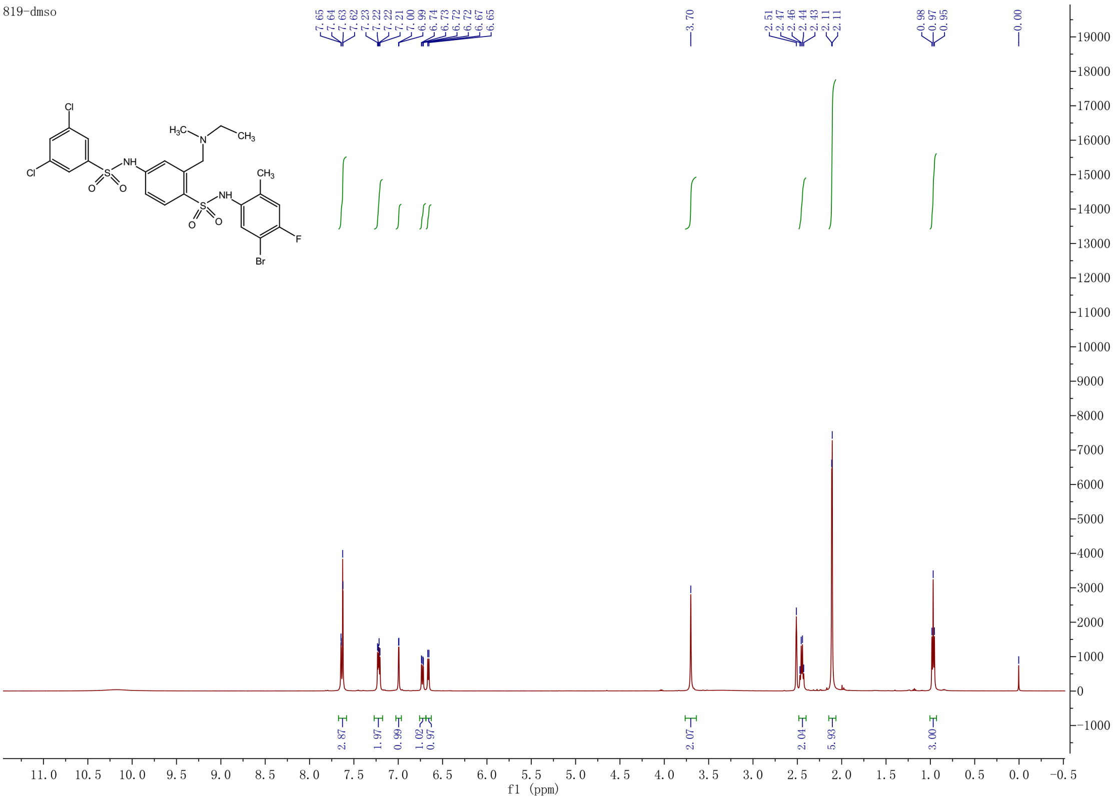
819-dmso
7.65
7.64
7.63
7.62
7.23
7.22
7.22
7.21
7.00
6.99
6.74
6.73
6.72
6.72
6.67
6.65
3.70
2.51
2.47
2.46
2.44
2.43
2.11
2.11
0.98
0.97
0.95
0.00
2.87
1.97
0.99
1.02
0.97
2.07
2.04
5.93
3.00
f1 (ppm)

MDL-820

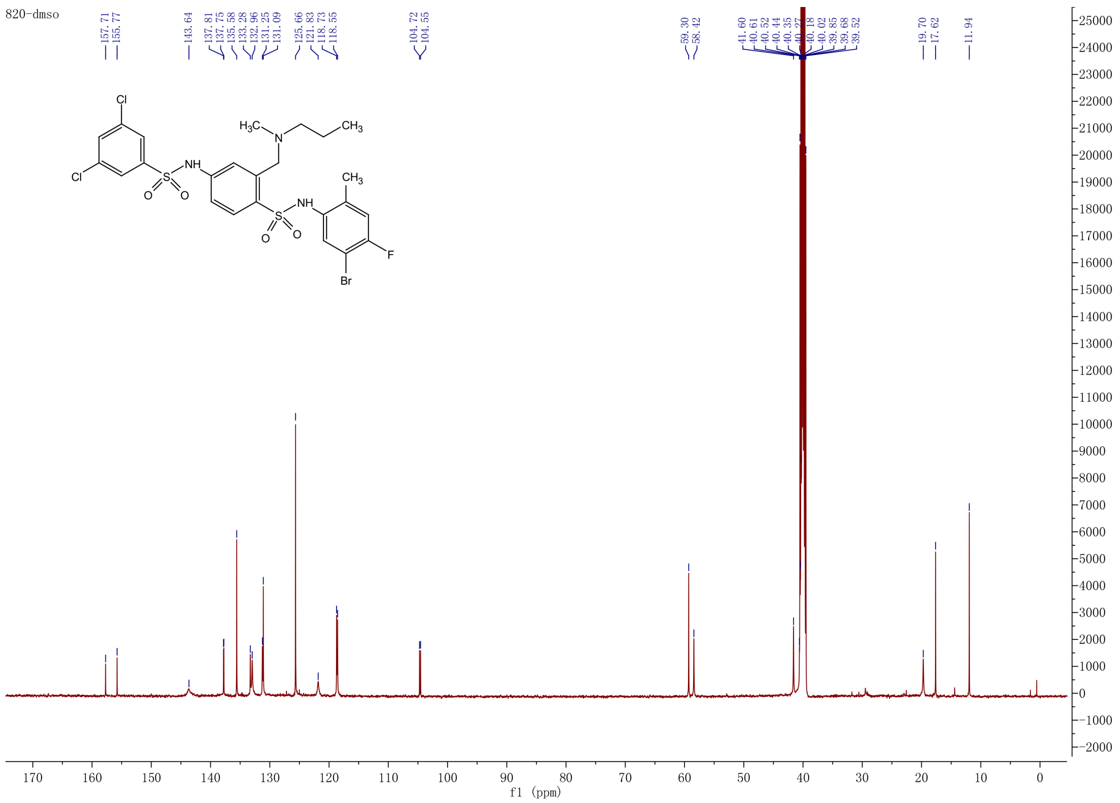
820-dmso
157.71
155.77
143.64
137.81
137.75
135.58
133.28
132.96
131.25
131.09
125.66
121.83
118.73
118.55
104.72
104.55
59.30
58.42
41.60
40.61
40.52
40.44
40.35
40.27
40.18
40.02
39.85
39.68
39.52
19.70
17.62
11.94
f1 (ppm)

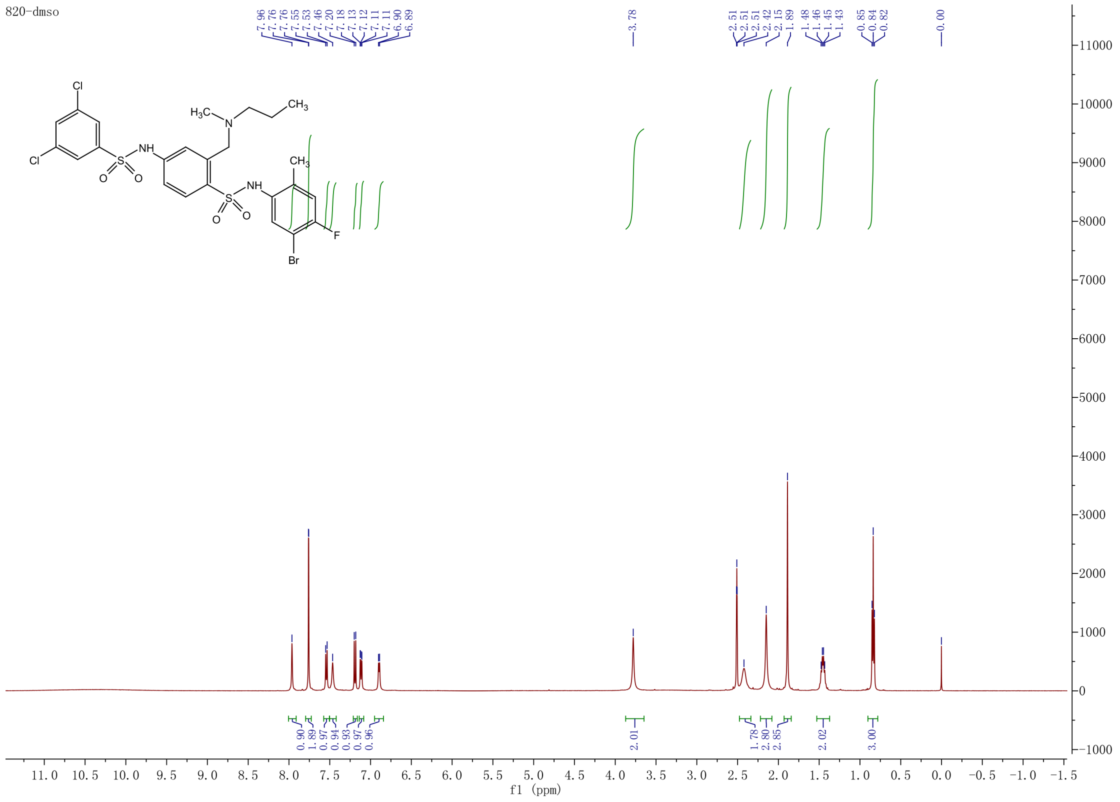
820-dmso
7.96
7.76
7.76
7.55
7.53
7.46
7.20
7.18
7.13
7.12
7.11
7.11
6.90
6.89
3.78
2.51
2.51
2.51
2.42
2.15
1.89
1.48
1.46
1.45
1.43
0.85
0.84
0.82
0.00
0.90
1.89
0.97
0.94
0.93
0.97
0.96
2.01
1.78
2.80
2.85
2.02
3.00
f1 (ppm)

MDL-821

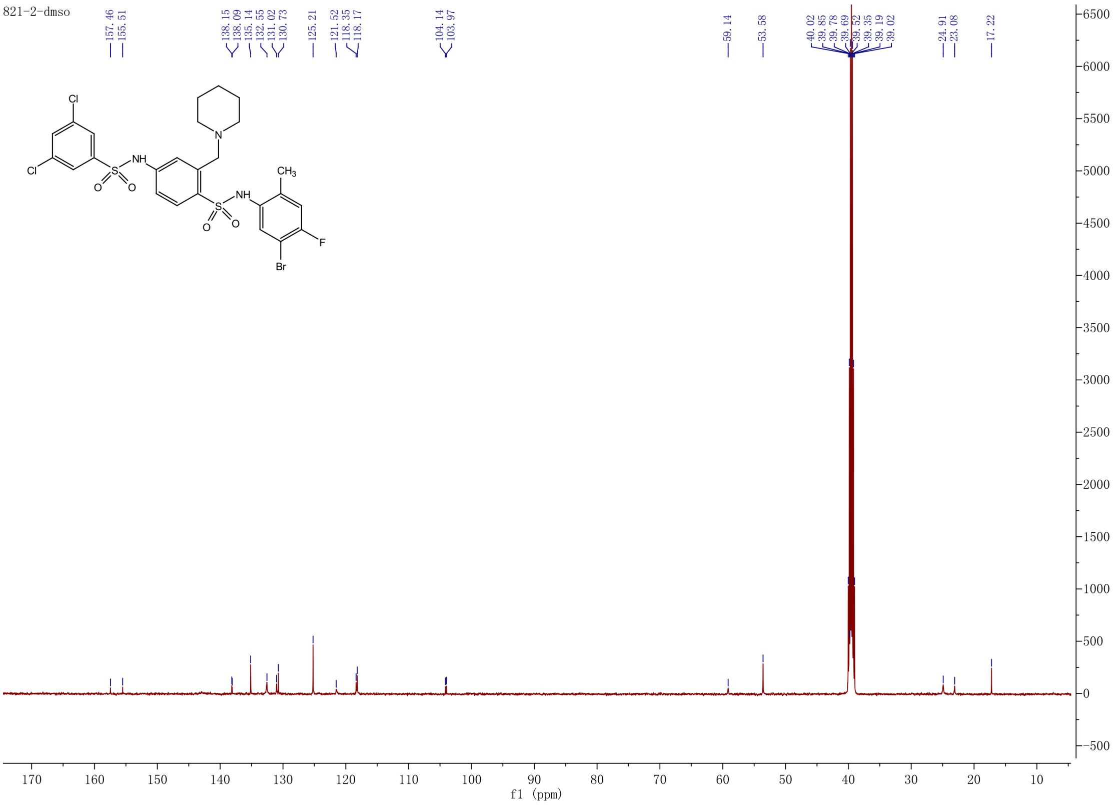
821-2-dmso
157.46
155.51
138.15
138.09
135.14
132.55
131.02
130.73
125.21
121.52
118.35
118.17
104.14
103.97
59.14
53.58
40.02
39.85
39.78
39.69
39.52
39.35
39.19
39.02
24.91
23.08
17.22
Cl
Cl
NH
N
S
O
O
NH
CH3
F
Br
f1 (ppm)

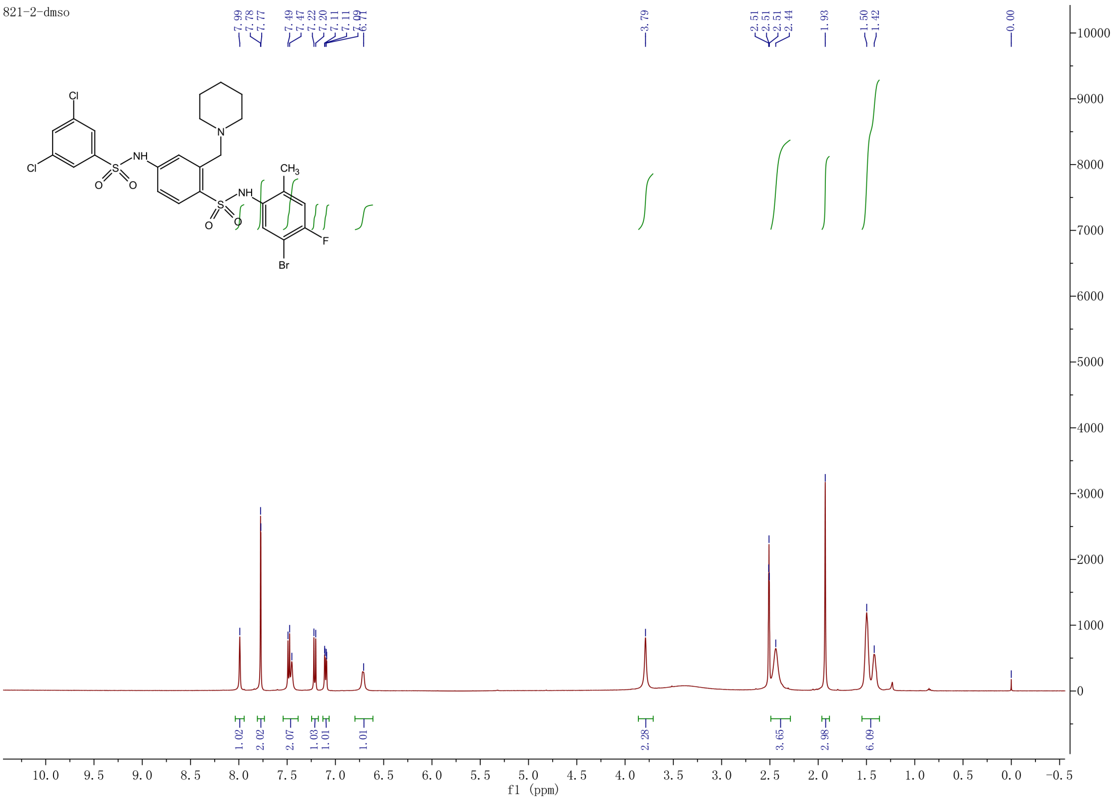
821-2-dmso
7.99
7.78
7.77
7.49
7.47
7.22
7.20
7.11
7.11
7.09
6.71
3.79
2.51
2.51
2.51
2.44
1.93
1.50
1.42
0.00
1.02
2.02
2.07
1.03
1.01
1.01
2.28
3.65
2.98
6.09
Cl
Cl
NH
N
S
O
O
NH
CH3
F
Br
f1 (ppm)

MDL-822

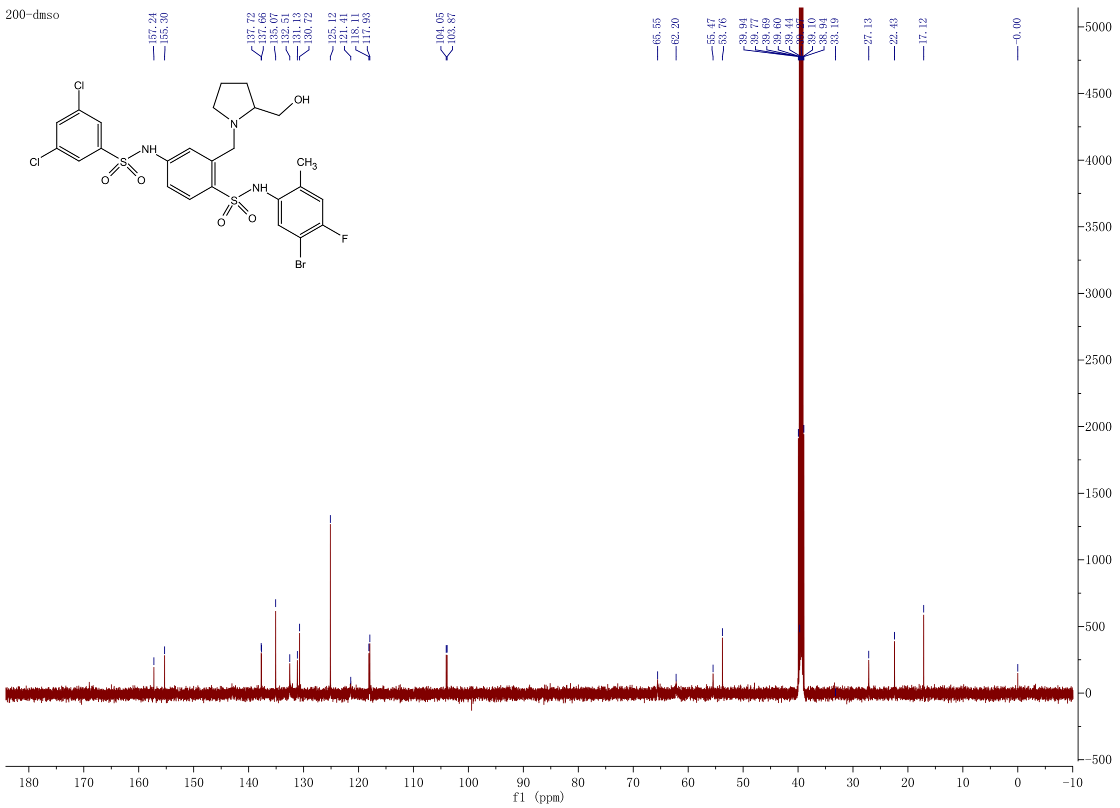

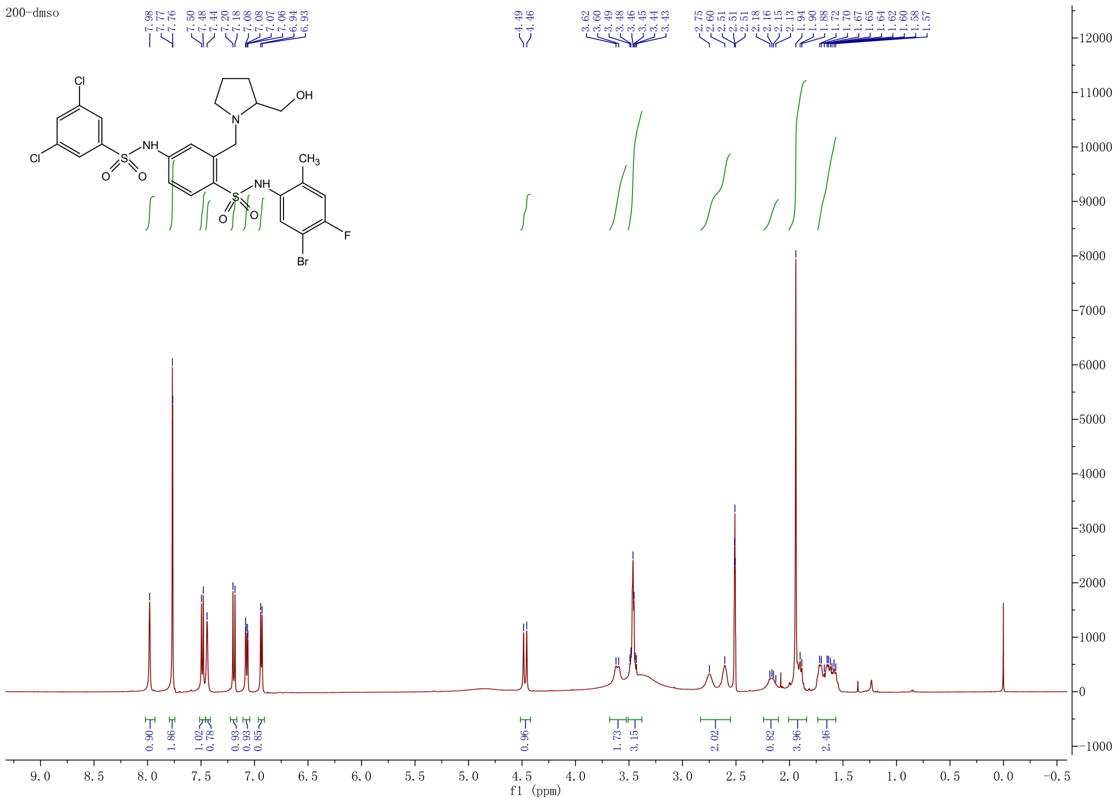

Relate to “Synthesis of compounds MDL analogs”. NMR spectra of compounds MDL analogs were shown.

HPLC analysis data of compounds MDL analogs

HPLC analysis data of compounds MDL analogs. The purities of identified compounds that were essential to the conclusions drawn in the text and determined by one standard instrumentation with one system given in the following table. The peak purity was checked with UV spectra.

		Method		
Equipment		Agilent 1260 with quaternary pump, photodiode array detector (DAD)		
Column		Agilent Zorbax Exlipse Plus C18 (100×4.6 mm, 3.5 µm particle size)		
System condition		a. CH_3CN(0.1% TFA)/H_2O (0.1% TFA), 10% (v/v) of CH_3CN(0.1% TFA) at the first time. Next, percentage of CH_3CN(0.1% TFA) was slowly increased to 100% in 15 min and the condition was maintained at 5 min, flow rate: 1.0 mL/min, calculated the relative purity of each compound at 254 nM.	b. CH_3CN(0.1% TFA)/H_2O (0.1% TFA), 10% (v/v) of CH_3CN(0.1% TFA) at the first time. Next, percentage of CH_3CN(0.1% TFA) was slowly increased to 100% in 15 min and the condition was maintained at 5 min, flow rate: 1.0 mL/min, calculated the relative purity of each compound at 280 nM.	
		c. CH_3CN(0.1% TFA)/H_2O (0.1% TFA), 10% (v/v) of CH_3CN(0.1% TFA) at the first time. Next, percentage of CH_3CN(0.1% TFA) was slowly increased to 100% in 15 min and the condition was maintained at 5 min, flow rate: 1.2 mL/min, calculated the relative purity of each compound at 254 nM.	d. CH_3CN(0.1% TFA)/H_2O (0.1% TFA), 10% (v/v) of CH_3CN(0.1% TFA) at the first time. Next, percentage of CH_3CN(0.1% TFA) was slowly increased to 100% in 15 min and the condition was maintained at 5 min, flow rate: 1.2 mL/min, calculated the relative purity of each compound at 280 nM.	
Result	Compound	Retention time (min)	Relactive purity (%)	
			254 nM	280 nM
	811	15.132	[a]98.28	[b]98.48
	812	13.736	[a]99.51	[b]99.82
	813	16.439	[a]98.53	[b]98.42
	814	13.361	[a]96.00	[b]95.92
	815	15.186	[a]99.55	[b]99.47
	816	15.963	[a]98.00	[b]98.35

	817	13.224	[a]95.09	[b]95.31
	818	11.772	[c]98.86	[d]99.41
	819	12.009	[c]99.38	[d]99.43
	820	12.483	[c]98.76	[d]99.18
	821	12.474	[c]99.58	[d]99.57
	822	10.483	[c]97.78	[d]99.22

HPLC UV spectra data of purity of compounds MDL analogs.

MDL-811

```
Data File C:\CHEM32\1\DATA\FCOMPOUND\TEST 2019-05-20 16-17-05\811.D
Sample Name: 811

    =====================================================================
    Acq. Operator   : SYSTEM                          Seq. Line :    2
    Acq. Instrument : 1260lc                           Location : Vial 25
    Injection Date  : 5/20/2019 4:40:37 PM                  Inj :    1
                                                     Inj Volume : 2.000 µl
    Different Inj Volume from Sample Entry!  Actual Inj Volume : 5.000 µl
    Method          : C:\CHEM32\1\DATA\FCOMPOUND\TEST 2019-05-20 16-17-05\TEST12.M (Sequence
                      Method)
    Last changed    : 5/20/2019 4:17:05 PM by SYSTEM
    Additional Info : Peak(s) manually integrated
```

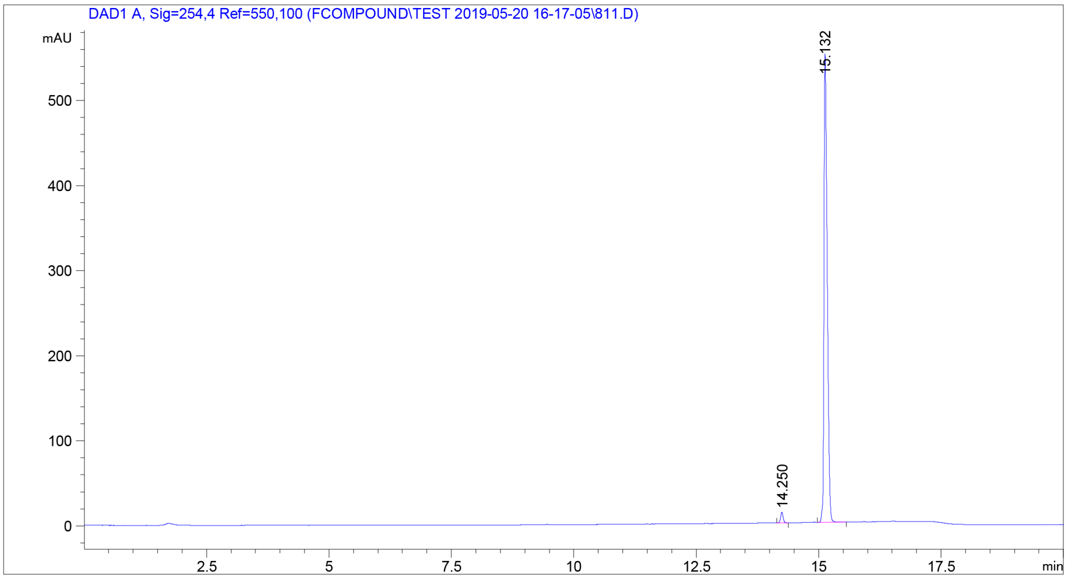


```
    =====================================================================
                            Area Percent Report
    =====================================================================

    Sorted By             :      Signal
    Multiplier            :      1.0000
    Dilution              :      1.0000
    Use Multiplier & Dilution Factor with ISTDs


    Signal 1: DAD1 A, Sig=254,4 Ref=550,100

    Peak RetTime Type  Width     Area      Height     Area
      #   [min]        [min]   [mAU*s]     [mAU]        %
    ----|-------|----|-------|----------|----------|--------|
       1  14.250 BB    0.0551   44.22514   12.39342   1.7187
       2  15.132 VB    0.0705 2528.98633  550.99341  98.2813

    Totals :                  2573.21146  563.38683


    =====================================================================
                             *** End of Report ***
```

Data File C:\CHEM32\1\DATA\FCOMPOUND\TEST 2019-05-20 16-17-05\811.D
Sample Name: 811

===

Acq. Operator : SYSTEM   Seq. Line : 2
Acq. Instrument : 1260lc   Location : Vial 25
Injection Date : 5/20/2019 4:40:37 PM   Inj : 1
                                          Inj Volume : 2.000 µl
Different Inj Volume from Sample Entry! Actual Inj Volume : 5.000 µl
Method : C:\CHEM32\1\DATA\FCOMPOUND\TEST 2019-05-20 16-17-05\TEST12.M (Sequence Method)
Last changed : 5/20/2019 4:17:05 PM by SYSTEM

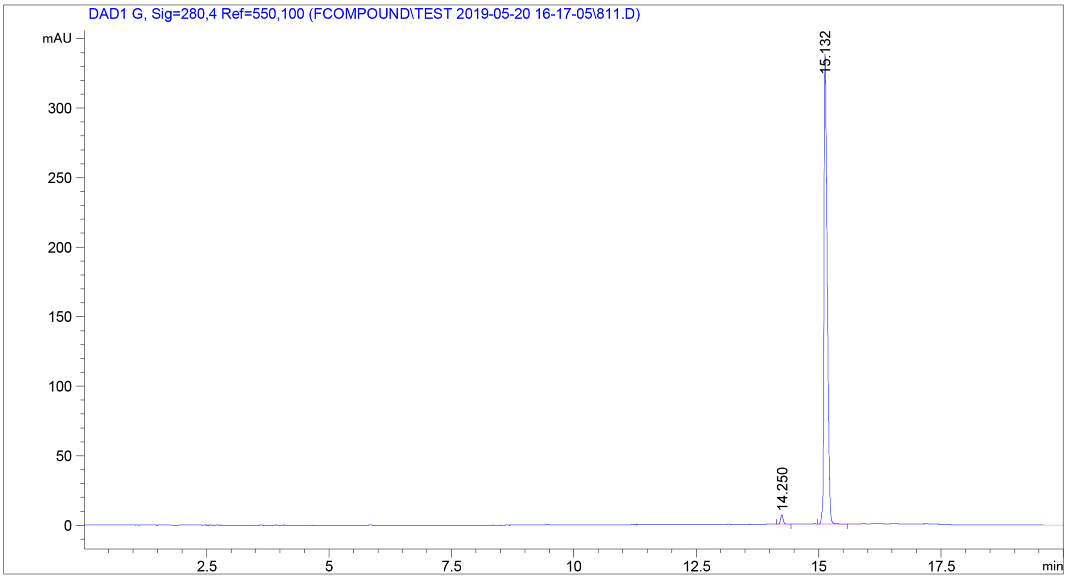

===

Area Percent Report

===

Sorted By : Signal
Multiplier : 1.0000
Dilution : 1.0000
Use Multiplier & Dilution Factor with ISTDs

Signal 1: DAD1 G, Sig=280,4 Ref=550,100

Peak #	RetTime [min]	Type	Width [min]	Area [mAU*s]	Height [mAU]	Area %
1	14.250	BB	0.0554	23.89845	6.66109	1.5168
2	15.132	BB	0.0705	1551.70544	338.23843	98.4832

Totals : 1575.60390 344.89952

===

*** End of Report ***

MDL-812

Data File C:\CHEM32\1\DATA\FCOMPOUND\TEST 2019-05-20 16-17-05\812.D
Sample Name: 812

===
Acq. Operator   : SYSTEM   Seq. Line :   3
Acq. Instrument : 1260lc   Location : Vial 26
Injection Date : 5/20/2019 5:02:27 PM   Inj :   1
                                               Inj Volume : 2.000 µl
Different Inj Volume from Sample Entry! Actual Inj Volume : 10.000 µl
Method   : C:\CHEM32\1\DATA\FCOMPOUND\TEST 2019-05-20 16-17-05\TEST12.M (Sequence Method)
Last changed   : 5/20/2019 4:17:05 PM by SYSTEM
Additional Info : Peak(s) manually integrated

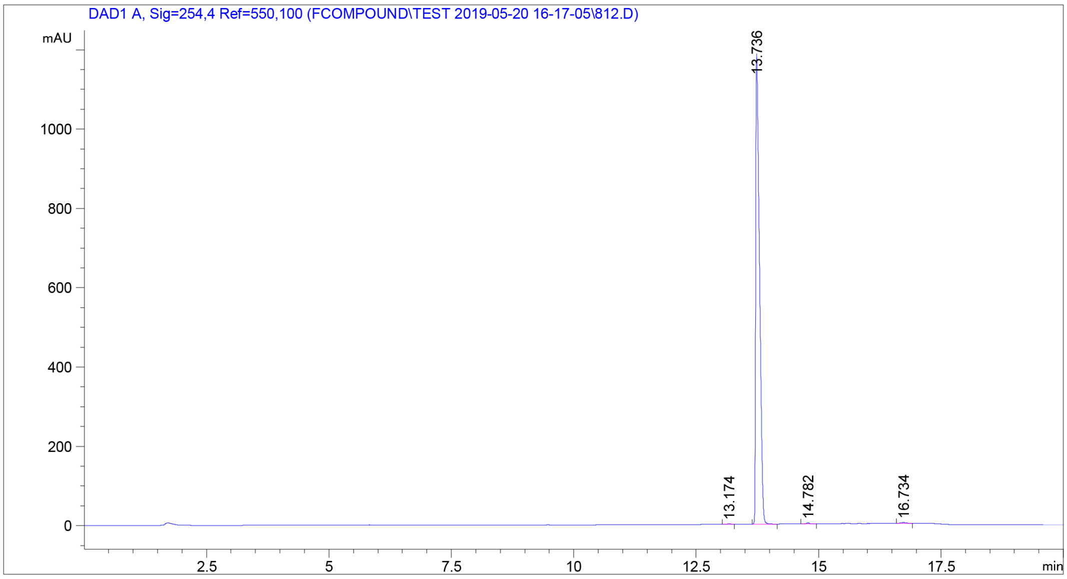

===
Area Percent Report
===

Sorted By : Signal
Multiplier : 1.0000
Dilution : 1.0000
Use Multiplier & Dilution Factor with ISTDs

Signal 1: DAD1 A, Sig=254,4 Ref=550,100

Peak #	RetTime [min]	Type	Width [min]	Area [mAU*s]	Height [mAU]	Area %
1	13.174	BB	0.0547	6.77659	1.87163	0.1058
2	13.736	VB	0.0822	6374.10400	1186.53284	99.5194
3	14.782	BB	0.0595	10.87952	2.78801	0.1699
4	16.734	BB	0.1150	13.12411	1.73899	0.2049

Totals : 6404.88423 1192.93146

```
Data File C:\CHEM32\1\DATA\FCOMPOUND\TEST 2019-05-20 16-17-05\812.D
Sample Name: 812

    =====================================================================
    Acq. Operator   : SYSTEM                        Seq. Line :   3
    Acq. Instrument : 1260lc                         Location : Vial 26
    Injection Date  : 5/20/2019 5:02:27 PM                  Inj :   1
                                                   Inj Volume : 2.000 µl
    Different Inj Volume from Sample Entry!  Actual Inj Volume : 10.000 µl
    Method          : C:\CHEM32\1\DATA\FCOMPOUND\TEST 2019-05-20 16-17-05\TEST12.M (Sequence
                      Method)
    Last changed    : 5/20/2019 4:17:05 PM by SYSTEM
    Additional Info : Peak(s) manually integrated
```

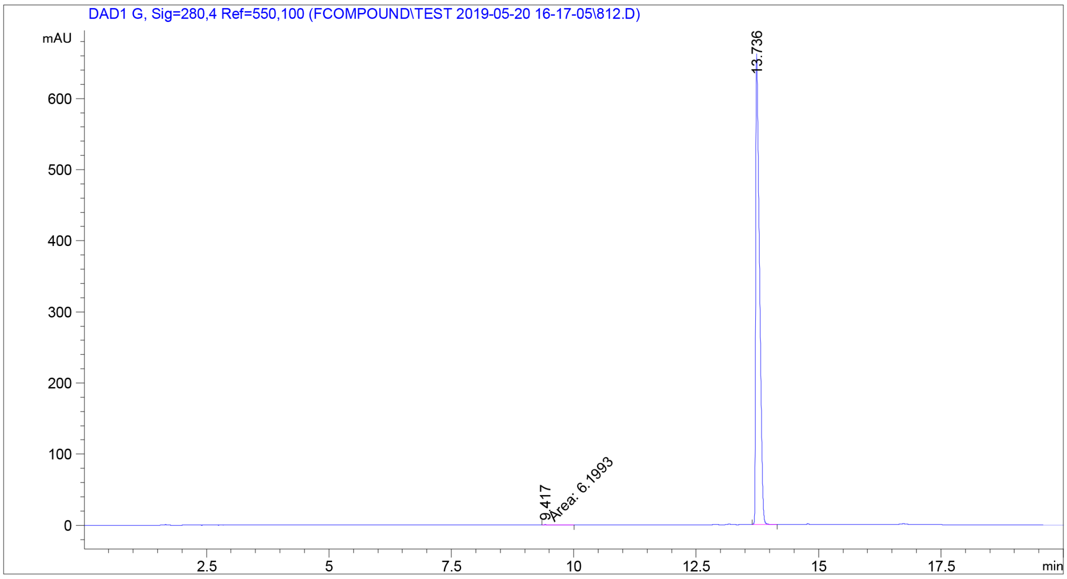


```
    =====================================================================
                             Area Percent Report
    =====================================================================

    Sorted By             :      Signal
    Multiplier            :      1.0000
    Dilution              :      1.0000
    Use Multiplier & Dilution Factor with ISTDs


    Signal 1: DAD1 G, Sig=280,4 Ref=550,100

    Peak RetTime Type  Width     Area      Height     Area
      #   [min]        [min]   [mAU*s]     [mAU]        %
    ----|-------|----|-------|----------|----------|--------|
       1   9.417 MM    0.1517    6.19930 6.81243e-1   0.1739
       2  13.736 VB    0.0822 3557.91553  662.20483  99.8261

    Totals :                  3564.11483  662.88608


    =====================================================================
                              *** End of Report ***
```

MDL-813

Data File C:\CHEM32\1\DATA\FCOMPOUND\TEST 2019-05-20 16-17-05\813.D
Sample Name: 813

===

```
Acq. Operator   : SYSTEM                        Seq. Line :   4
Acq. Instrument : 1260lc                         Location : Vial 27
Injection Date  : 5/20/2019 5:24:16 PM                Inj :   1
                                               Inj Volume : 2.000 µl
Different Inj Volume from Sample Entry!  Actual Inj Volume : 8.000 µl
Method          : C:\CHEM32\1\DATA\FCOMPOUND\TEST 2019-05-20 16-17-05\TEST12.M (Sequence
                  Method)
Last changed    : 5/20/2019 4:17:05 PM by SYSTEM
Additional Info : Peak(s) manually integrated
```

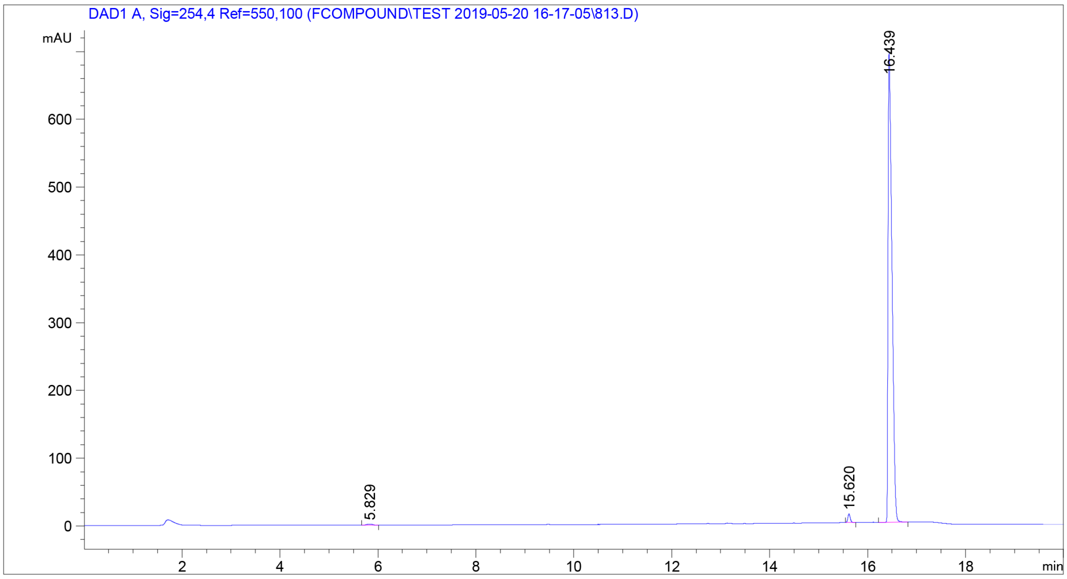

===

Area Percent Report

===

```
Sorted By             :      Signal
Multiplier            :      1.0000
Dilution              :      1.0000
Use Multiplier & Dilution Factor with ISTDs
```

Signal 1: DAD1 A, Sig=254,4 Ref=550,100

Peak #	RetTime [min]	Type	Width [min]	Area [mAU*s]	Height [mAU]	Area %
1	5.829	BB	0.1166	11.75607	1.54736	0.3027
2	15.620	VB	0.0544	45.33770	12.93765	1.1674
3	16.439	BB	0.0856	3826.52124	691.46991	98.5299
Totals :				3883.61501	705.95492	

===

Data File C:\CHEM32\1\DATA\FCOMPOUND\TEST 2019-05-20 16-17-05\813.D
Sample Name: 813

===

Acq. Operator : SYSTEM Seq. Line : 4
Acq. Instrument : 1260lc Location : Vial 27
Injection Date : 5/20/2019 5:24:16 PM Inj : 1
Inj Volume : 2.000 µl
Different Inj Volume from Sample Entry! Actual Inj Volume : 8.000 µl
Method : C:\CHEM32\1\DATA\FCOMPOUND\TEST 2019-05-20 16-17-05\TEST12.M (Sequence Method)
Last changed : 5/20/2019 4:17:05 PM by SYSTEM

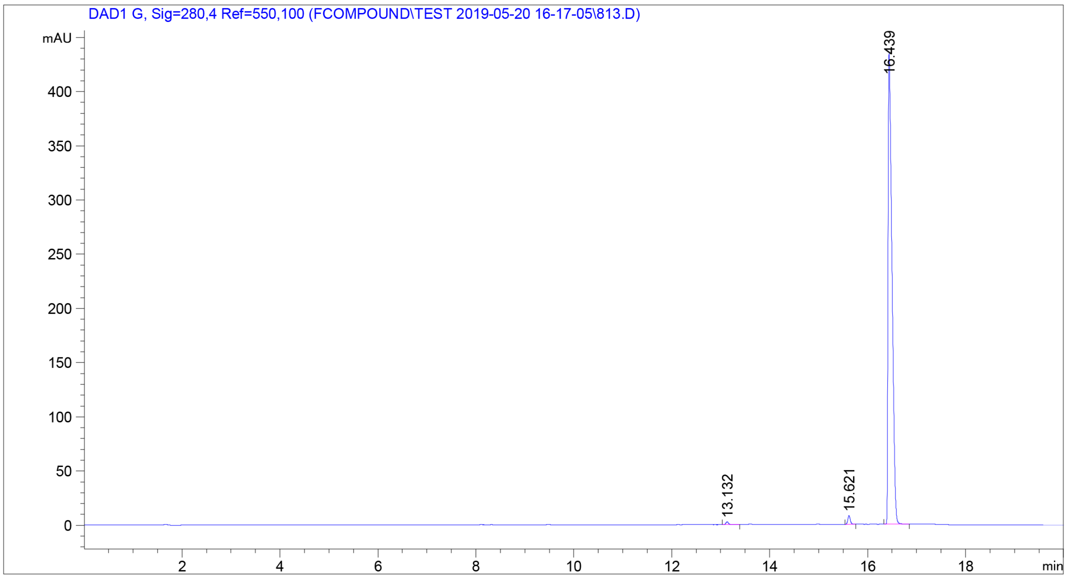

===

Area Percent Report

===

Sorted By : Signal
Multiplier : 1.0000
Dilution : 1.0000
Use Multiplier & Dilution Factor with ISTDs

Signal 1: DAD1 G, Sig=280,4 Ref=550,100

Peak #	RetTime [min]	Type	Width [min]	Area [mAU*s]	Height [mAU]	Area %
1	13.132	BB	0.0563	10.49832	2.79365	0.4302
2	15.621	BB	0.0542	28.01473	8.03633	1.1479
3	16.439	BB	0.0856	2402.01440	434.00583	98.4219

Totals : 2440.52746 444.83580

===

*** End of Report ***

MDL-814

Data File C:\CHEM32\1\DATA\FCOMPOUND\TEST 2019-05-20 16-17-05\814.D
Sample Name: 814

```
=====================================================================
Acq. Operator   : SYSTEM                         Seq. Line :    5
Acq. Instrument : 1260lc                          Location : Vial 28
Injection Date  : 5/20/2019 5:46:06 PM                 Inj :    1
                                                Inj Volume : 2.000 µl
Different Inj Volume from Sample Entry!  Actual Inj Volume : 8.000 µl
Method          : C:\CHEM32\1\DATA\FCOMPOUND\TEST 2019-05-20 16-17-05\TEST12.M (Sequence
                  Method)
Last changed    : 5/20/2019 4:17:05 PM by SYSTEM
Additional Info : Peak(s) manually integrated
```

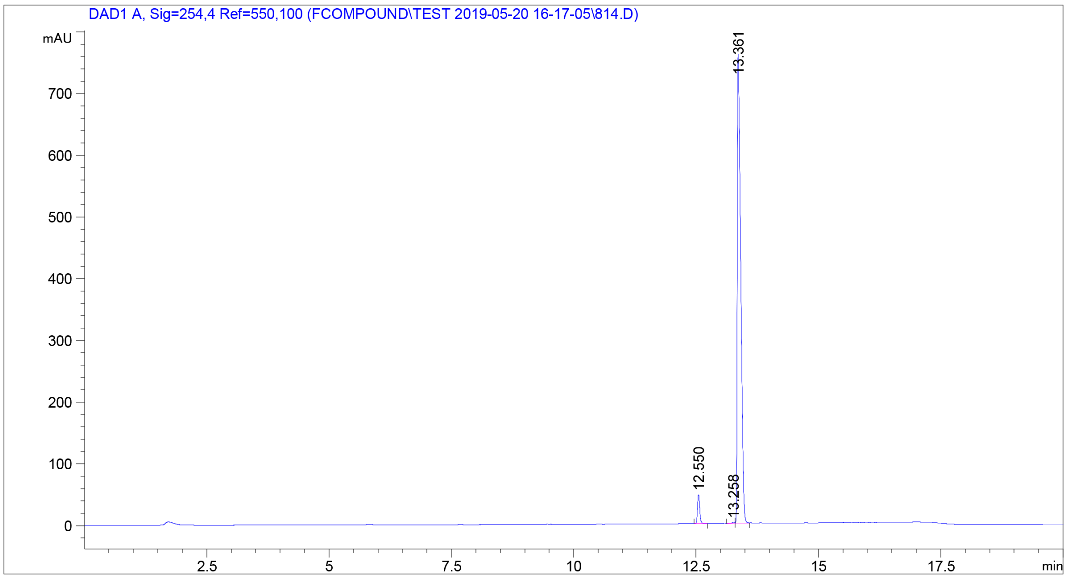


```
=====================================================================
                         Area Percent Report
=====================================================================

Sorted By             :      Signal
Multiplier            :      1.0000
Dilution              :      1.0000
Use Multiplier & Dilution Factor with ISTDs


Signal 1: DAD1 A, Sig=254,4 Ref=550,100
```

Peak #	RetTime [min]	Type	Width [min]	Area [mAU*s]	Height [mAU]	Area %
1	12.550	BB	0.0480	148.06168	47.30540	3.8326
2	13.258	BV	0.0507	6.27082	1.86637	0.1623
3	13.361	VB	0.0749	3708.91333	760.61719	96.0051

Totals : 3863.24583 809.78896

===

Data File C:\CHEM32\1\DATA\FCOMPOUND\TEST 2019-05-20 16-17-05\814.D
Sample Name: 814

===

Acq. Operator : SYSTEM   Seq. Line : 5
Acq. Instrument : 1260lc   Location : Vial 28
Injection Date : 5/20/2019 5:46:06 PM   Inj : 1
Inj Volume : 2.000 µl
Different Inj Volume from Sample Entry! Actual Inj Volume : 8.000 µl
Method : C:\CHEM32\1\DATA\FCOMPOUND\TEST 2019-05-20 16-17-05\TEST12.M (Sequence Method)
Last changed : 5/20/2019 4:17:05 PM by SYSTEM

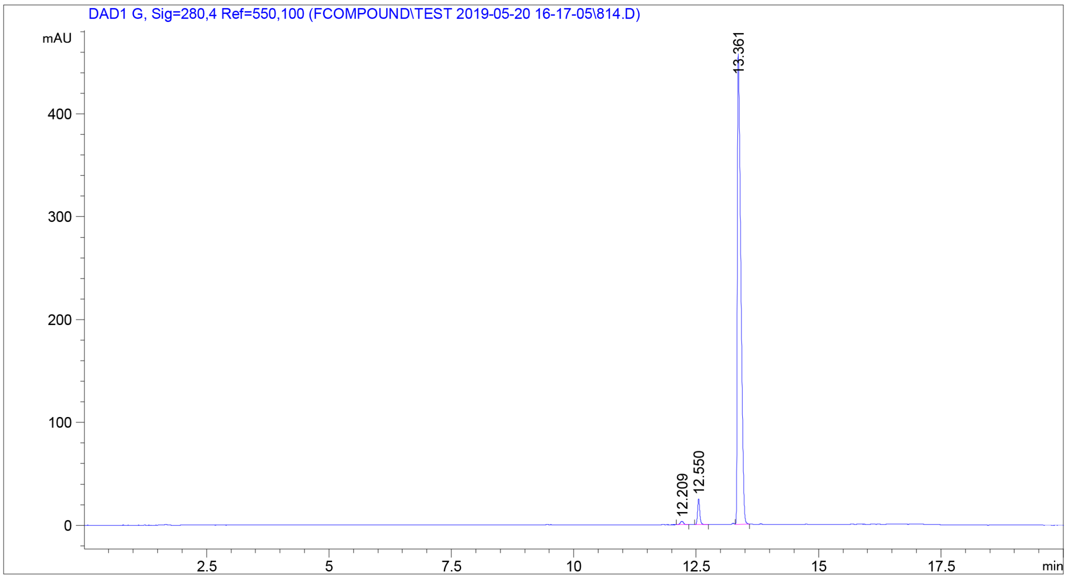

===

Area Percent Report

===

Sorted By : Signal
Multiplier : 1.0000
Dilution : 1.0000
Use Multiplier & Dilution Factor with ISTDs

Signal 1: DAD1 G, Sig=280,4 Ref=550,100

Peak #	RetTime [min]	Type	Width [min]	Area [mAU*s]	Height [mAU]	Area %
1	12.209	BB	0.0734	15.14836	3.27949	0.6507
2	12.550	BB	0.0483	79.78844	25.32793	3.4272
3	13.361	VB	0.0749	2233.15820	457.59863	95.9221

Totals : 2328.09500 486.20606

===

*** End of Report ***

MDL-815

Data File C:\CHEM32\1\DATA\FCOMPOUND\TEST 2019-05-20 16-17-05\815.D
Sample Name: 815

===

Acq. Operator : SYSTEM Seq. Line : 6
Acq. Instrument : 1260lc Location : Vial 29
Injection Date : 5/20/2019 6:07:55 PM Inj : 1
Inj Volume : 2.000 µl
Different Inj Volume from Sample Entry! Actual Inj Volume : 10.000 µl
Method : C:\CHEM32\1\DATA\FCOMPOUND\TEST 2019-05-20 16-17-05\TEST12.M (Sequence Method)
Last changed : 5/20/2019 4:17:05 PM by SYSTEM
Additional Info : Peak(s) manually integrated

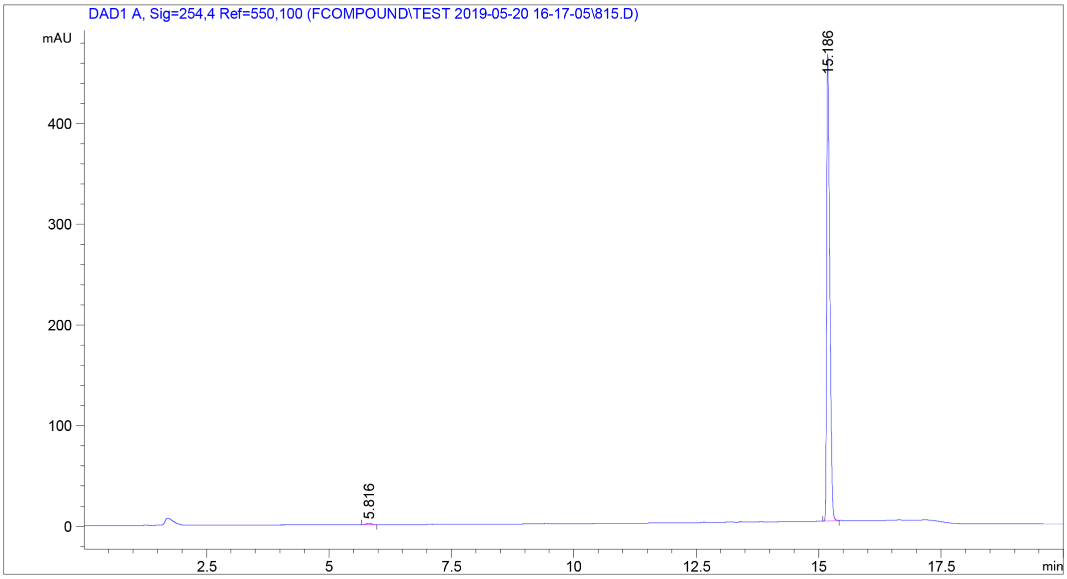

===

Area Percent Report

===

Sorted By : Signal
Multiplier : 1.0000
Dilution : 1.0000
Use Multiplier & Dilution Factor with ISTDs

Signal 1: DAD1 A, Sig=254,4 Ref=550,100

Peak #	RetTime [min]	Type	Width [min]	Area [mAU*s]	Height [mAU]	Area %
1	5.816	BB	0.1004	9.23906	1.18561	0.4516
2	15.186	BB	0.0677	2036.49268	464.31644	99.5484

Totals : 2045.73174 465.50205

===

*** End of Report ***

Data File C:\CHEM32\1\DATA\FCOMPOUND\TEST 2019-05-20 16-17-05\815.D
Sample Name: 815

===

Acq. Operator : SYSTEM   Seq. Line : 6
Acq. Instrument : 1260lc   Location : Vial 29
Injection Date : 5/20/2019 6:07:55 PM   Inj : 1
Inj Volume : 2.000 µl
Different Inj Volume from Sample Entry! Actual Inj Volume : 10.000 µl
Method : C:\CHEM32\1\DATA\FCOMPOUND\TEST 2019-05-20 16-17-05\TEST12.M (Sequence Method)
Last changed : 5/20/2019 4:17:05 PM by SYSTEM
Additional Info : Peak(s) manually integrated

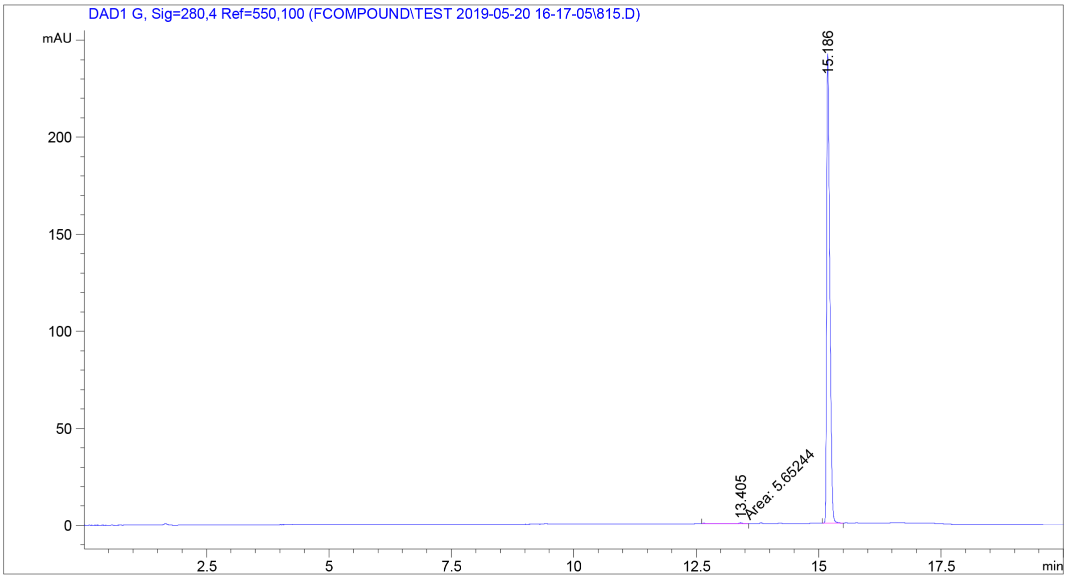

===

Area Percent Report

===

Sorted By : Signal
Multiplier : 1.0000
Dilution : 1.0000
Use Multiplier & Dilution Factor with ISTDs

Signal 1: DAD1 G, Sig=280,4 Ref=550,100

Peak #	RetTime [min]	Type	Width [min]	Area [mAU*s]	Height [mAU]	Area %
1	13.405	MM	0.2193	5.65244	4.29613e-1	0.5286
2	15.186	BB	0.0678	1063.58484	241.95203	99.4714

Totals : 1069.23728 242.38164

===

*** End of Report ***

MDL-816

```
Data File C:\CHEM32\1\DATA\FCOMPOUND\TEST 2019-05-20 16-17-05\816.D
Sample Name: 816

    =====================================================================
    Acq. Operator   : SYSTEM                        Seq. Line :    7
    Acq. Instrument : 1260lc                         Location : Vial 30
    Injection Date  : 5/20/2019 6:29:46 PM                Inj :    1
                                                   Inj Volume : 2.000 µl
    Different Inj Volume from Sample Entry!  Actual Inj Volume : 6.000 µl
    Method          : C:\CHEM32\1\DATA\FCOMPOUND\TEST 2019-05-20 16-17-05\TEST12.M (Sequence
                      Method)
    Last changed    : 5/20/2019 4:17:05 PM by SYSTEM
    Additional Info : Peak(s) manually integrated
```

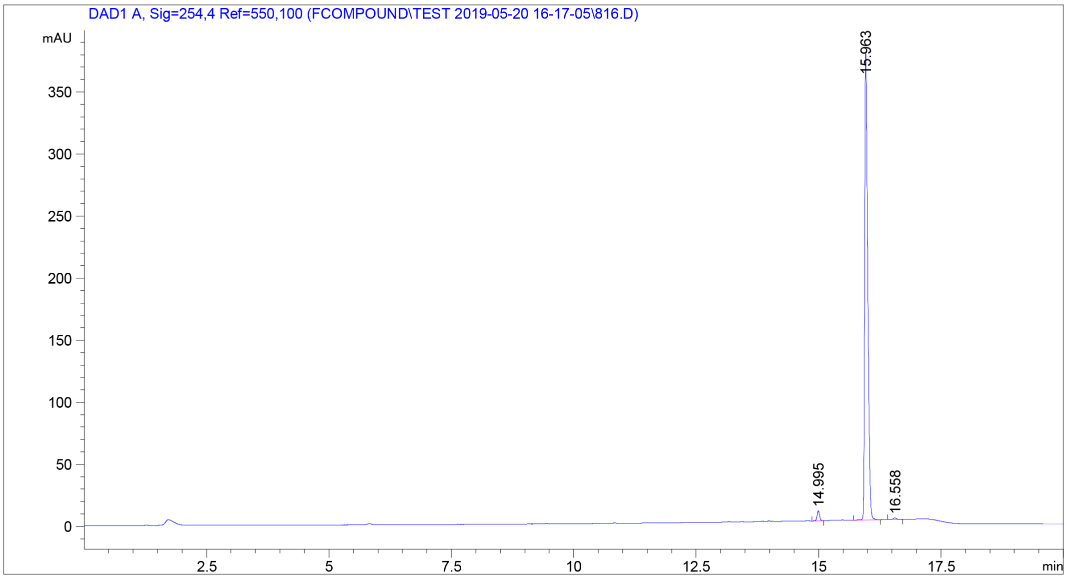


```
    =====================================================================
                             Area Percent Report
    =====================================================================

    Sorted By             :      Signal
    Multiplier            :      1.0000
    Dilution              :      1.0000
    Use Multiplier & Dilution Factor with ISTDs


    Signal 1: DAD1 A, Sig=254,4 Ref=550,100
```

Peak #	RetTime [min]	Type	Width [min]	Area [mAU*s]	Height [mAU]	Area %
1	14.995	BB	0.0542	28.12886	8.06285	1.6854
2	15.963	BB	0.0668	1635.56042	375.72974	97.9955
3	16.558	BB	0.0608	5.32693	1.30085	0.3192

```
    Totals :                  1669.01622  385.09344


    =====================================================================
```

Data File C:\CHEM32\1\DATA\FCOMPOUND\TEST 2019-05-20 16-17-05\816.D
Sample Name: 816

===

Acq. Operator : SYSTEM Seq. Line : 7
Acq. Instrument : 1260lc Location : Vial 30
Injection Date : 5/20/2019 6:29:46 PM Inj : 1
Inj Volume : 2.000 µl
Different Inj Volume from Sample Entry! Actual Inj Volume : 6.000 µl
Method : C:\CHEM32\1\DATA\FCOMPOUND\TEST 2019-05-20 16-17-05\TEST12.M (Sequence Method)
Last changed : 5/20/2019 4:17:05 PM by SYSTEM

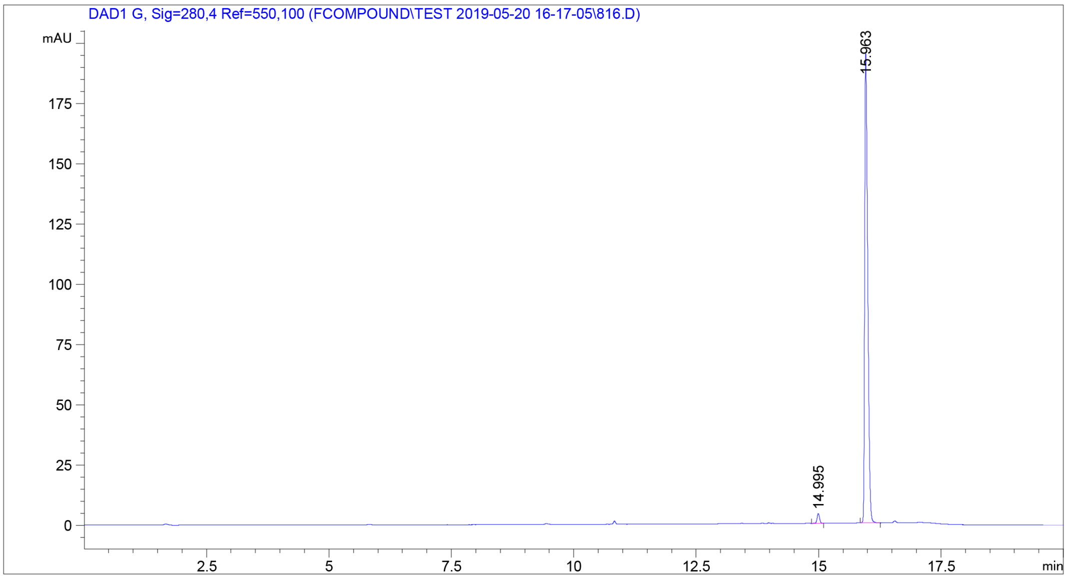

===

Area Percent Report

===

Sorted By : Signal
Multiplier : 1.0000
Dilution : 1.0000
Use Multiplier & Dilution Factor with ISTDs

Signal 1: DAD1 G, Sig=280,4 Ref=550,100

Peak #	RetTime [min]	Type	Width [min]	Area [mAU*s]	Height [mAU]	Area %
1	14.995	BB	0.0542	14.24673	4.08325	1.6534
2	15.963	BB	0.0673	847.40161	194.75302	98.3466

Totals : 861.64835 198.83627

===

*** End of Report ***

MDL-817

Data File C:\CHEM32\1\DATA\FCOMPOUND\TEST 2019-05-20 16-17-05\817.D
Sample Name: 817

===

Acq. Operator : SYSTEM Seq. Line : 8
Acq. Instrument : 1260lc Location : Vial 31
Injection Date : 5/20/2019 6:51:36 PM Inj : 1
Inj Volume : 2.000 µl
Different Inj Volume from Sample Entry! Actual Inj Volume : 6.000 µl
Method : C:\CHEM32\1\DATA\FCOMPOUND\TEST 2019-05-20 16-17-05\TEST12.M (Sequence Method)
Last changed : 5/20/2019 4:17:05 PM by SYSTEM
Additional Info : Peak(s) manually integrated

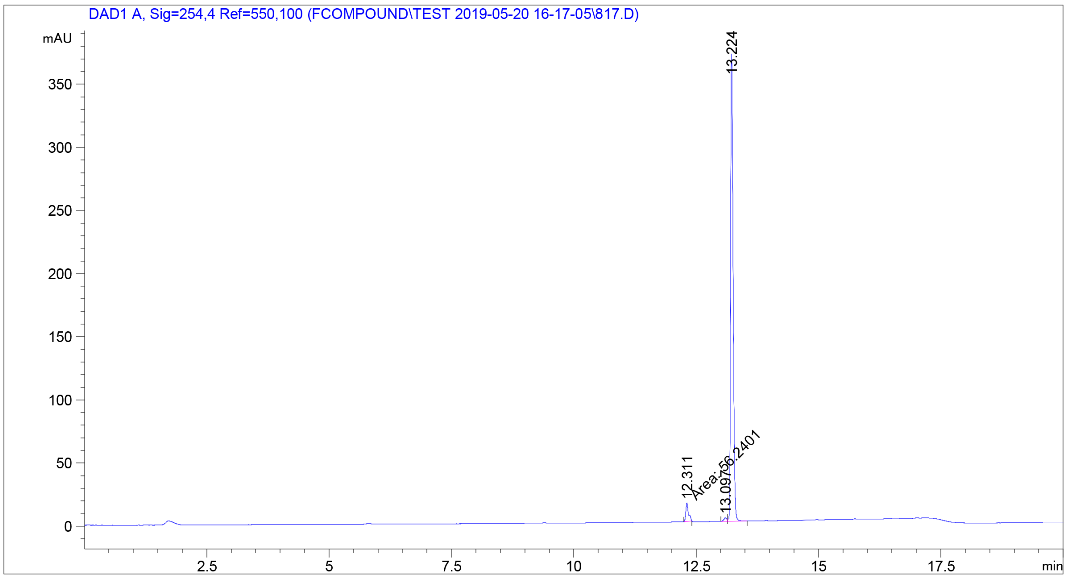

===
Area Percent Report
===

Sorted By : Signal
Multiplier : 1.0000
Dilution : 1.0000
Use Multiplier & Dilution Factor with ISTDs

Signal 1: DAD1 A, Sig=254,4 Ref=550,100

Peak #	RetTime [min]	Type	Width [min]	Area [mAU*s]	Height [mAU]	Area %
1	12.311	MM	0.0636	56.24012	14.74519	4.0391
2	13.097	VV	0.0638	12.09847	2.77612	0.8689
3	13.224	VB	0.0552	1324.04688	370.23776	95.0920

Totals : 1392.38546 387.75907

===

Data File C:\CHEM32\1\DATA\FCOMPOUND\TEST 2019-05-20 16-17-05\817.D
Sample Name: 817

===

Acq. Operator : SYSTEM Seq. Line : 8
Acq. Instrument : 1260lc Location : Vial 31
Injection Date : 5/20/2019 6:51:36 PM Inj : 1
Inj Volume : 2.000 µl
Different Inj Volume from Sample Entry! Actual Inj Volume : 6.000 µl
Method : C:\CHEM32\1\DATA\FCOMPOUND\TEST 2019-05-20 16-17-05\TEST12.M (Sequence Method)
Last changed : 5/20/2019 4:17:05 PM by SYSTEM
Additional Info : Peak(s) manually integrated

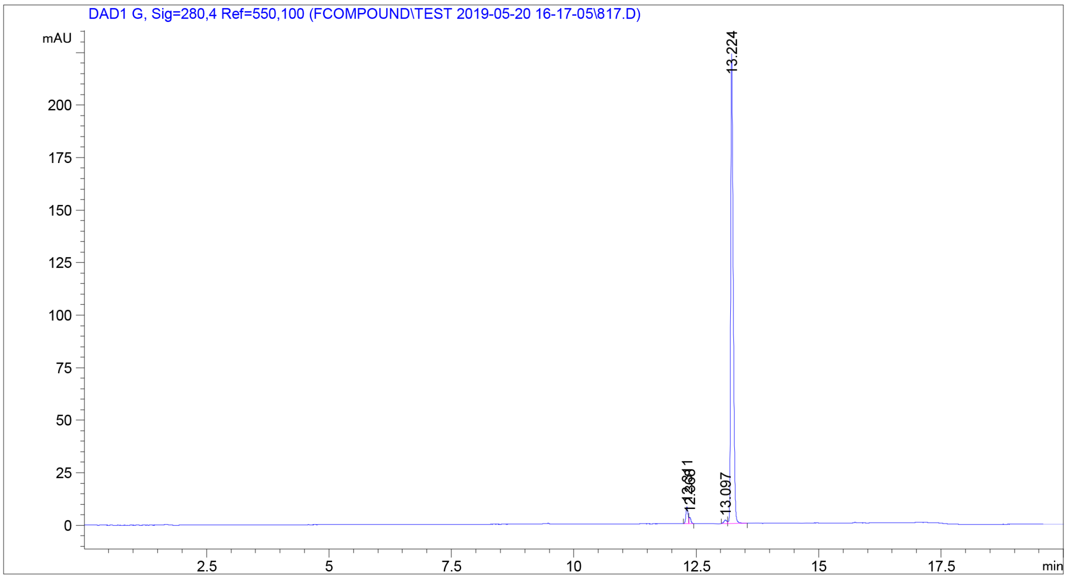

===

Area Percent Report

===

Sorted By : Signal
Multiplier : 1.0000
Dilution : 1.0000
Use Multiplier & Dilution Factor with ISTDs

Signal 1: DAD1 G, Sig=280,4 Ref=550,100

Peak #	RetTime [min]	Type	Width [min]	Area [mAU*s]	Height [mAU]	Area %
1	12.311	BV	0.0470	24.66913	7.99953	2.9406
2	12.368	VB	0.0396	7.80047	3.07238	0.9298
3	13.097	VV	0.0626	6.86336	1.61494	0.8181
4	13.224	VB	0.0552	799.58600	223.55717	95.3115

Totals : 838.91896 236.24402

MDL-818

Data File C:\CHEM32\1\DATA\FCOMPOUND\TEST 2020-03-03 11-11-11\ME-ME.D
Sample Name: Me-Me

===

Acq. Operator : SYSTEM Seq. Line : 5
Acq. Instrument : 1260lc Location : Vial 65
Injection Date : 3/3/2020 12:40:06 PM Inj : 1
Inj Volume : 2.000 µl
Different Inj Volume from Sample Entry! Actual Inj Volume : 6.000 µl
Method : C:\CHEM32\1\DATA\FCOMPOUND\TEST 2020-03-03 11-11-11\TEST12.M (Sequence Method)
Last changed : 3/3/2020 11:11:11 AM by SYSTEM
Additional Info : Peak(s) manually integrated

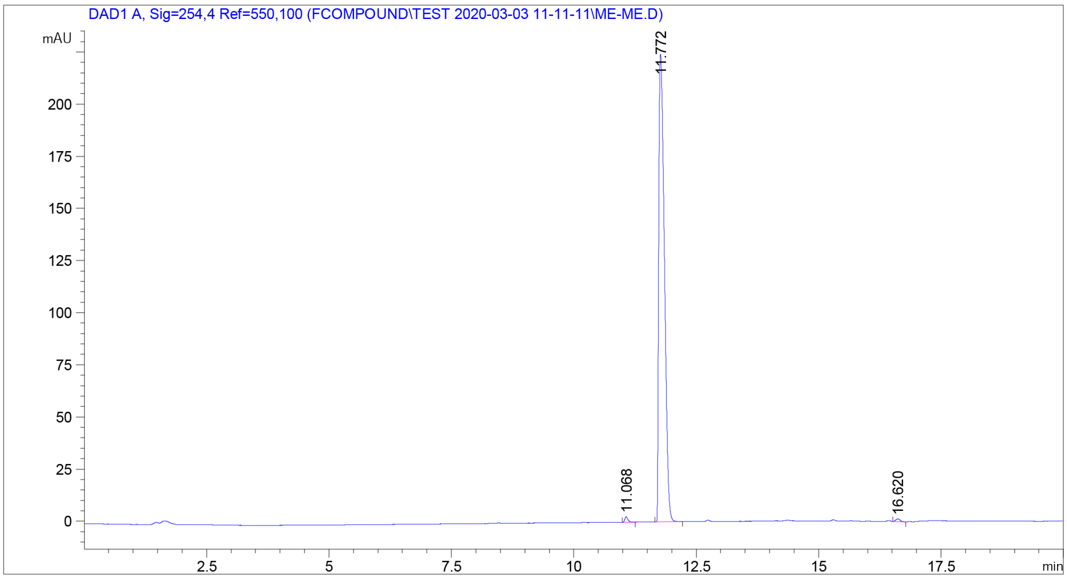

===

Area Percent Report

===

Sorted By : Signal
Multiplier : 1.0000
Dilution : 1.0000
Use Multiplier & Dilution Factor with ISTDs

Signal 1: DAD1 A, Sig=254,4 Ref=550,100

Peak #	RetTime [min]	Type	Width [min]	Area [mAU*s]	Height [mAU]	Area %
1	11.068	BB	0.0647	11.79943	2.71694	0.6496
2	11.772	BB	0.1294	1795.72339	224.37698	98.8628
3	16.620	VB	0.0917	8.85562	1.42138	0.4875

Totals : 1816.37844 228.51530

===

Data File C:\CHEM32\1\DATA\FCOMPOUND\TEST 2020-03-03 11-11-11\ME-ME.D
Sample Name: Me-Me

===

Acq. Operator : SYSTEM Seq. Line : 5
Acq. Instrument : 1260lc Location : Vial 65
Injection Date : 3/3/2020 12:40:06 PM Inj : 1
Inj Volume : 2.000 µl
Different Inj Volume from Sample Entry! Actual Inj Volume : 6.000 µl
Method : C:\CHEM32\1\DATA\FCOMPOUND\TEST 2020-03-03 11-11-11\TEST12.M (Sequence Method)
Last changed : 3/3/2020 11:11:11 AM by SYSTEM

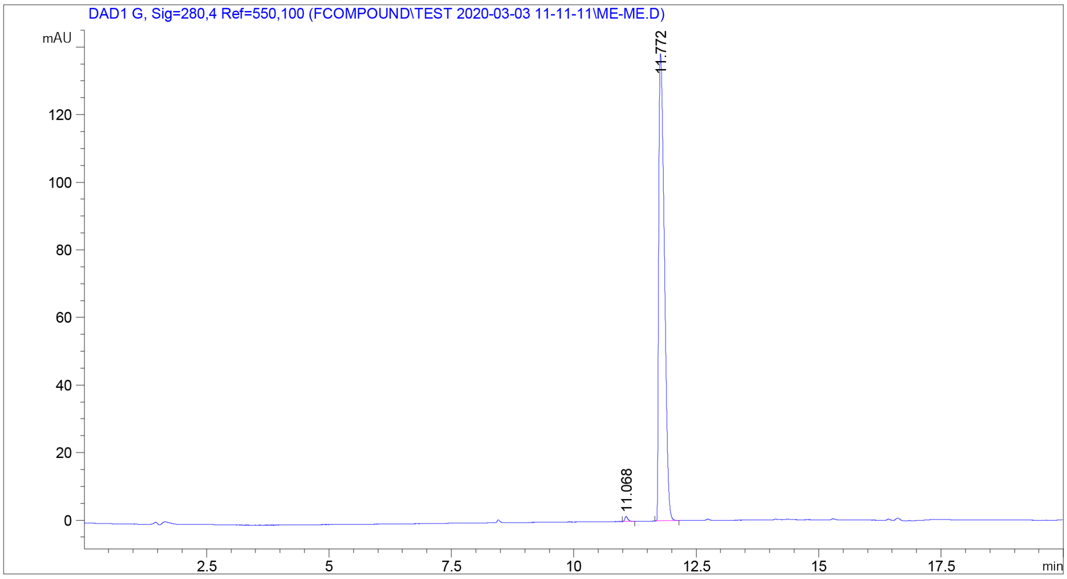

===

Area Percent Report

===

Sorted By : Signal
Multiplier : 1.0000
Dilution : 1.0000
Use Multiplier & Dilution Factor with ISTDs

Signal 1: DAD1 G, Sig=280,4 Ref=550,100

Peak #	RetTime [min]	Type	Width [min]	Area [mAU*s]	Height [mAU]	Area %
1	11.068	BB	0.0666	6.54671	1.49455	0.5886
2	11.772	BB	0.1293	1105.68530	138.27916	99.4114

Totals : 1112.23201 139.77371

===

*** End of Report ***

MDL-819

Data File C:\CHEM32\1\DATA\FCOMPOUND\TEST 2020-03-03 11-11-11\ME-ET.D
Sample Name: Me-Et

===

Acq. Operator : SYSTEM Seq. Line : 6
Acq. Instrument : 1260lc Location : Vial 66
Injection Date : 3/3/2020 1:01:53 PM Inj : 1
Inj Volume : 2.000 µl
Different Inj Volume from Sample Entry! Actual Inj Volume : 6.000 µl
Method : C:\CHEM32\1\DATA\FCOMPOUND\TEST 2020-03-03 11-11-11\TEST12.M (Sequence Method)
Last changed : 3/3/2020 11:11:11 AM by SYSTEM
Additional Info : Peak(s) manually integrated

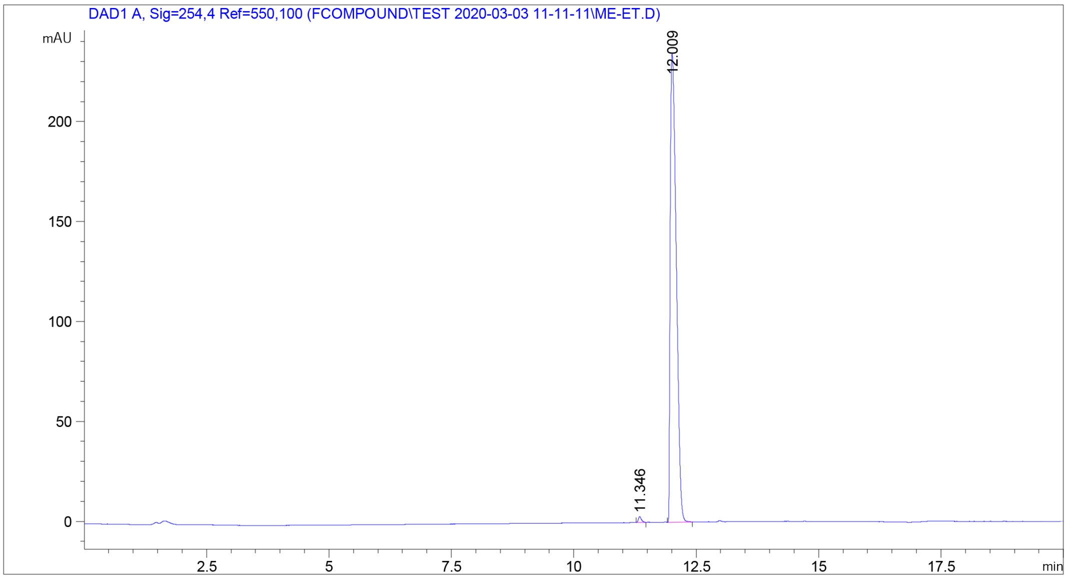

===

Area Percent Report

===

Sorted By : Signal
Multiplier : 1.0000
Dilution : 1.0000
Use Multiplier & Dilution Factor with ISTDs

Signal 1: DAD1 A, Sig=254,4 Ref=550,100

Peak #	RetTime [min]	Type	Width [min]	Area [mAU*s]	Height [mAU]	Area %
1	11.346	BB	0.0637	12.47002	2.95845	0.6193
2	12.009	BB	0.1371	2001.16431	234.39781	99.3807
Totals :				2013.63433	237.35626	

===

*** End of Report ***

Data File C:\CHEM32\1\DATA\FCOMPOUND\TEST 2020-03-03 11-11-11\ME-ET.D
Sample Name: Me-Et

===

Acq. Operator : SYSTEM Seq. Line : 6
Acq. Instrument : 1260lc Location : Vial 66
Injection Date : 3/3/2020 1:01:53 PM Inj : 1
Inj Volume : 2.000 µl
Different Inj Volume from Sample Entry! Actual Inj Volume : 6.000 µl
Method : C:\CHEM32\1\DATA\FCOMPOUND\TEST 2020-03-03 11-11-11\TEST12.M (Sequence Method)
Last changed : 3/3/2020 11:11:11 AM by SYSTEM

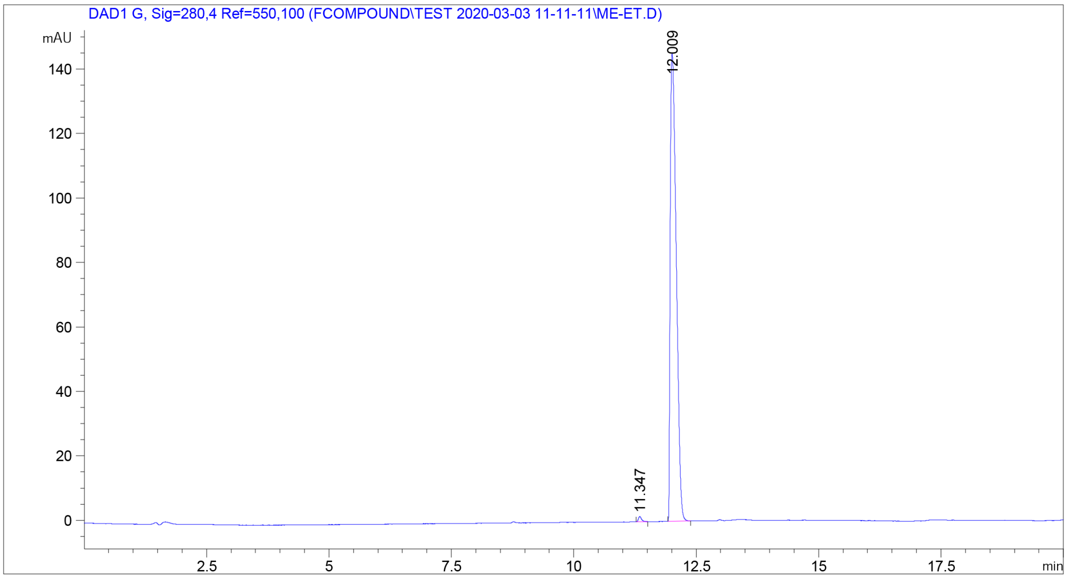

===

Area Percent Report

===

Sorted By : Signal
Multiplier : 1.0000
Dilution : 1.0000
Use Multiplier & Dilution Factor with ISTDs

Signal 1: DAD1 G, Sig=280,4 Ref=550,100

Peak #	RetTime [min]	Type	Width [min]	Area [mAU*s]	Height [mAU]	Area %
1	11.347	BB	0.0663	7.12819	1.63676	0.5720
2	12.009	BB	0.1371	1238.99194	145.11757	99.4280

Totals : 1246.12013 146.75433

===

*** End of Report ***

MDL-820

Data File C:\CHEM32\1\DATA\FCOMPOUND\TEST 2020-03-03 11-11-11\ME-PR.D
Sample Name: Me-Pr

===

Acq. Operator : SYSTEM Seq. Line : 7
Acq. Instrument : 1260lc Location : Vial 67
Injection Date : 3/3/2020 1:23:40 PM Inj : 1
Inj Volume : 2.000 µl
Different Inj Volume from Sample Entry! Actual Inj Volume : 6.000 µl
Method : C:\CHEM32\1\DATA\FCOMPOUND\TEST 2020-03-03 11-11-11\TEST12.M (Sequence Method)
Last changed : 3/3/2020 11:11:11 AM by SYSTEM
Additional Info : Peak(s) manually integrated

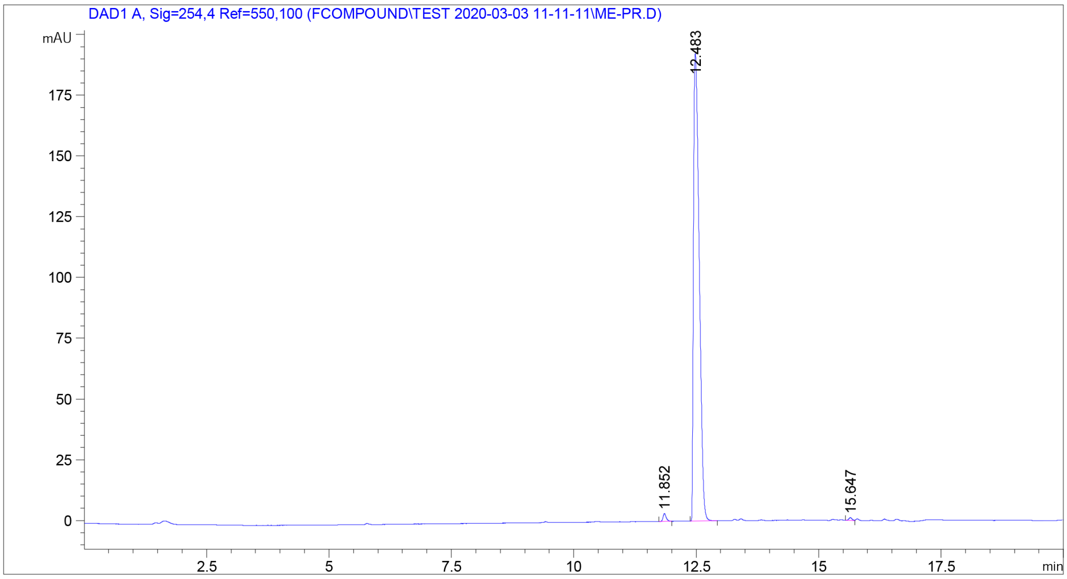

===

Area Percent Report

===

Sorted By : Signal
Multiplier : 1.0000
Dilution : 1.0000
Use Multiplier & Dilution Factor with ISTDs

Signal 1: DAD1 A, Sig=254,4 Ref=550,100

Peak #	RetTime [min]	Type	Width [min]	Area [mAU*s]	Height [mAU]	Area %
1	11.852	BB	0.0667	14.50987	3.27323	0.9185
2	12.483	BB	0.1312	1560.13611	192.28294	98.7560
3	15.647	BV	0.0710	5.14208	1.15293	0.3255

Totals : 1579.78806 196.70911

===

Data File C:\CHEM32\1\DATA\FCOMPOUND\TEST 2020-03-03 11-11-11\ME-PR.D
Sample Name: Me-Pr

===

Acq. Operator : SYSTEM Seq. Line : 7
Acq. Instrument : 1260lc Location : Vial 67
Injection Date : 3/3/2020 1:23:40 PM Inj : 1
Inj Volume : 2.000 µl
Different Inj Volume from Sample Entry! Actual Inj Volume : 6.000 µl
Method : C:\CHEM32\1\DATA\FCOMPOUND\TEST 2020-03-03 11-11-11\TEST12.M (Sequence Method)
Last changed : 3/3/2020 11:11:11 AM by SYSTEM

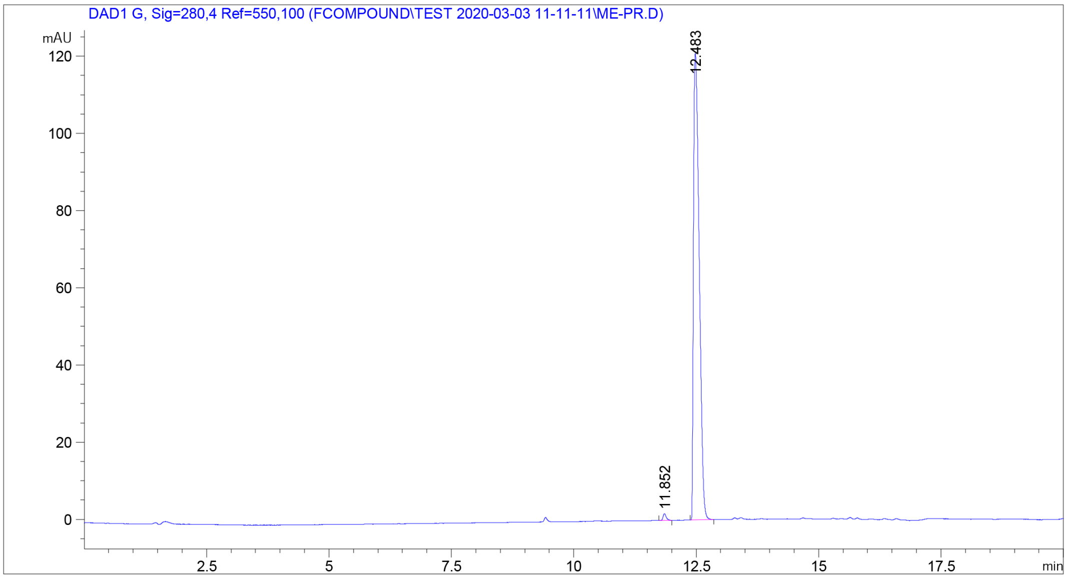

===

Area Percent Report

===

Sorted By : Signal
Multiplier : 1.0000
Dilution : 1.0000
Use Multiplier & Dilution Factor with ISTDs

Signal 1: DAD1 G, Sig=280,4 Ref=550,100

Peak #	RetTime [min]	Type	Width [min]	Area [mAU*s]	Height [mAU]	Area %
1	11.852	BB	0.0653	8.12277	1.81173	0.8225
2	12.483	BB	0.1301	979.44397	120.80373	99.1775

Totals : 987.56674 122.61545

===

*** End of Report ***

MDL-821

Data File C:\CHEM32\1\DATA\FCOMPOUND\TEST 2020-03-03 11-11-11\PEP-1.D
Sample Name: PEP-1

===

Acq. Operator : SYSTEM Seq. Line : 8
Acq. Instrument : 1260lc Location : Vial 68
Injection Date : 3/3/2020 1:45:27 PM Inj : 1
Inj Volume : 2.000 µl
Different Inj Volume from Sample Entry! Actual Inj Volume : 6.000 µl
Method : C:\CHEM32\1\DATA\FCOMPOUND\TEST 2020-03-03 11-11-11\TEST12.M (Sequence Method)
Last changed : 3/3/2020 11:11:11 AM by SYSTEM
Additional Info : Peak(s) manually integrated

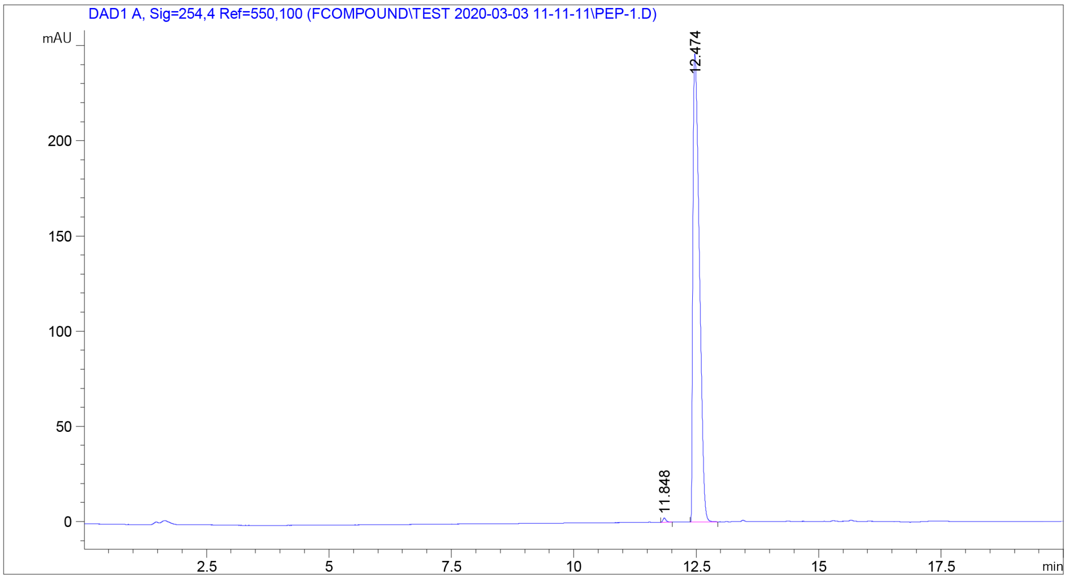

===

Area Percent Report

===

Sorted By : Signal
Multiplier : 1.0000
Dilution : 1.0000
Use Multiplier & Dilution Factor with ISTDs

Signal 1: DAD1 A, Sig=254,4 Ref=550,100

Peak #	RetTime [min]	Type	Width [min]	Area [mAU*s]	Height [mAU]	Area %
1	11.848	BB	0.0644	9.53240	2.18448	0.4227
2	12.474	BB	0.1475	2245.37402	245.92307	99.5773

Totals : 2254.90643 248.10755

===

*** End of Report ***

Data File C:\CHEM32\1\DATA\FCOMPOUND\TEST 2020-03-03 11-11-11\PEP-1.D
Sample Name: PEP-1

===

Acq. Operator : SYSTEM Seq. Line : 8
Acq. Instrument : 1260lc Location : Vial 68
Injection Date : 3/3/2020 1:45:27 PM Inj : 1
Inj Volume : 2.000 µl
Different Inj Volume from Sample Entry! Actual Inj Volume : 6.000 µl
Method : C:\CHEM32\1\DATA\FCOMPOUND\TEST 2020-03-03 11-11-11\TEST12.M (Sequence Method)
Last changed : 3/3/2020 11:11:11 AM by SYSTEM
Additional Info : Peak(s) manually integrated

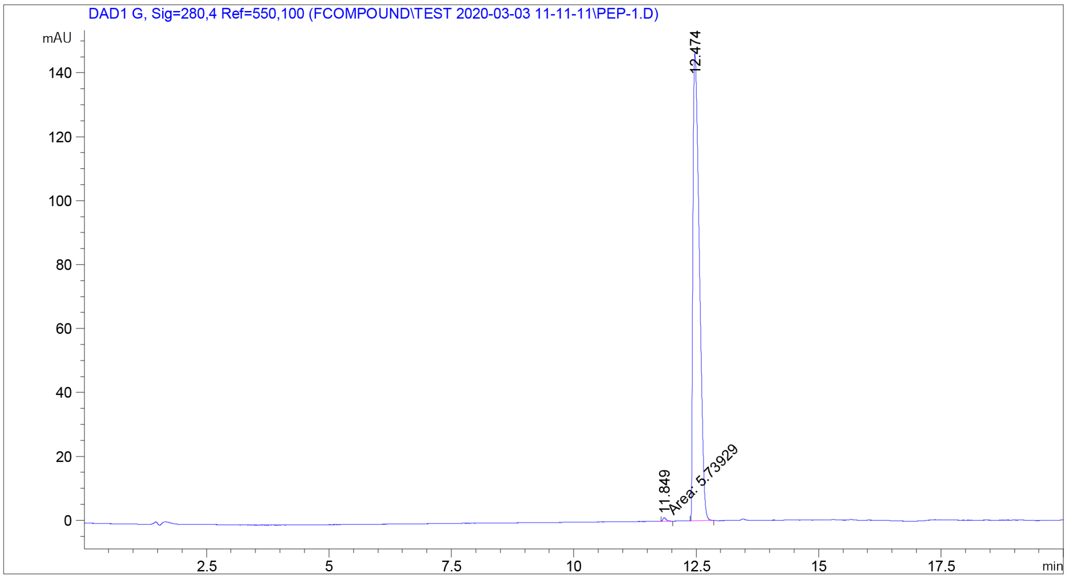

===

Area Percent Report

===

Sorted By : Signal
Multiplier : 1.0000
Dilution : 1.0000
Use Multiplier & Dilution Factor with ISTDs

Signal 1: DAD1 G, Sig=280,4 Ref=550,100

Peak #	RetTime [min]	Type	Width [min]	Area [mAU*s]	Height [mAU]	Area %
1	11.849	MM	0.0803	5.73929	1.19086	0.4282
2	12.474	BB	0.1475	1334.58997	146.18858	99.5718

Totals : 1340.32926 147.37944

===

*** End of Report ***

MDL-822

```
Data File C:\CHEM32\1\DATA\FCOMPOUND\TEST 2020-03-10 15-04-31\822.D
Sample Name: 822

    =====================================================================
    Acq. Operator   : SYSTEM                            Seq. Line :   8
    Acq. Instrument : 1260lc                             Location : Vial 8
    Injection Date  : 3/10/2020 5:38:57 PM                    Inj :   1
                                                       Inj Volume : 2.000 µl
    Different Inj Volume from Sample Entry!  Actual Inj Volume : 8.000 µl
    Method          : C:\CHEM32\1\DATA\FCOMPOUND\TEST 2020-03-10 15-04-31\TEST12.M (Sequence
                      Method)
    Last changed    : 3/10/2020 3:04:58 PM by SYSTEM
    Additional Info : Peak(s) manually integrated
```

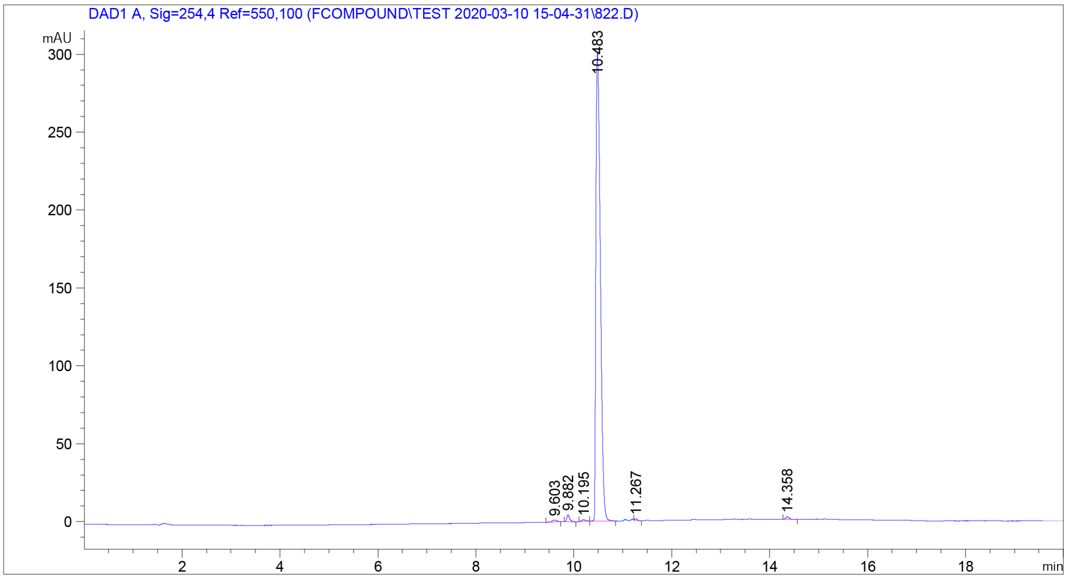


```
    =====================================================================
                             Area Percent Report
    =====================================================================

    Sorted By             :      Signal
    Multiplier            :      1.0000
    Dilution              :      1.0000
    Use Multiplier & Dilution Factor with ISTDs


    Signal 1: DAD1 A, Sig=254,4 Ref=550,100
```

Peak #	RetTime [min]	Type	Width [min]	Area [mAU*s]	Height [mAU]	Area %
1	9.603	BB	0.0885	7.52870	1.13340	0.3884
2	9.882	BB	0.0581	16.56597	4.28472	0.8546
3	10.195	BB	0.0912	6.59114	1.02264	0.3400
4	10.483	BB	0.1027	1895.50354	299.96573	97.7795
5	11.267	VB	0.0595	5.00499	1.19285	0.2582
6	14.358	BB	0.0687	7.35488	1.64479	0.3794

Totals : 1938.54922 309.24412

Data File C:\CHEM32\1\DATA\FCOMPOUND\TEST 2020-03-10 15-04-31\822.D
Sample Name: 822

===

Acq. Operator : SYSTEM Seq. Line : 8
Acq. Instrument : 1260lc Location : Vial 8
Injection Date : 3/10/2020 5:38:57 PM Inj : 1
Inj Volume : 2.000 µl
Different Inj Volume from Sample Entry! Actual Inj Volume : 8.000 µl
Method : C:\CHEM32\1\DATA\FCOMPOUND\TEST 2020-03-10 15-04-31\TEST12.M (Sequence Method)
Last changed : 3/10/2020 3:04:58 PM by SYSTEM

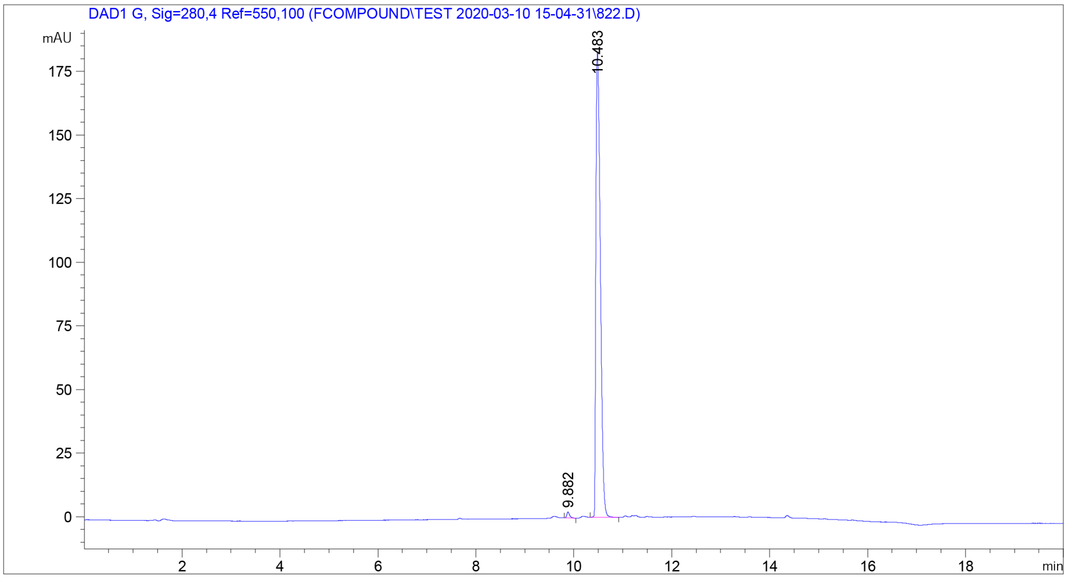

===

Area Percent Report

===

Sorted By : Signal
Multiplier : 1.0000
Dilution : 1.0000
Use Multiplier & Dilution Factor with ISTDs

Signal 1: DAD1 G, Sig=280,4 Ref=550,100

Peak #	RetTime [min]	Type	Width [min]	Area [mAU*s]	Height [mAU]	Area %
1	9.882	BB	0.0576	9.00800	2.30527	0.7752
2	10.483	BB	0.1023	1152.96228	182.31613	99.2248

Totals : 1161.97028 184.62141

===

*** End of Report ***

Supplementary Tables

Table S1. Plasma pharmacokinetic parameters of MDL-800 and MDL-811 in C57BL/6J mice. *

Compound	MDL-800	MDL-811
Administration	IP 30 mg/mL	IP 30 mg/mL
t_{max} (h)	0.50	0.50
C_{max} (ng/mL)	6610.46	5330.67
$AUC_{0\text{-}t}$ (h × ng/mL)	17831.51	19580.01
$AUC_{0\text{-}\infty}$ (h × ng/mL)	17883.09	19669.85
$t_{1/2}$ (h)	2.07	1.38
MRT (h)	2.47	2.63
F (%)	71.33	92.96

*The data represent the mean value from five C57BL/6J mice per group.

Table S2. Effect of MDL-811 among histone deacetylase enzymes.

Enzyme	Effect*
HDAC1	ND
HDAC2	ND
HDAC3	ND
HDAC4	ND
HDAC5	ND
HDAC6	ND
HDAC7	ND
HDAC8	ND
HDAC9	ND
HDAC10	ND
HDAC11	ND
SIRT1	ND
SIRT2	ND
SIRT3	ND
SIRT5	ND
SIRT6	Activation
SIRT7	ND

*ND represents no detectable activation or inhibition effect on the targets in the presence of MDL-811 at 100 μM. The data are from three independent experiments.

Table S3. Melting temperatures of SIRT6 treated with or without MDL-811 in the CETSA.

	**Melting Temperature (T_m) (°C) ***
DMSO	45.8 ± 0.3
10 μM MDL-811	47.6 ± 0.1

*The data represent the mean ± s.e.m. from four independent experiments.

Table S4. IC_{50} values of MDL-811 in CRC cell lines.

**CRC cell line**	**IC_{50} ± s.d. (μM) [*]**
NCI-H716	61.0 ± 2.3
Colo205	38.1 ± 4.4
Colo320DM	33.2 ± 0.9
LS1034	31.9 ± 0.7
Caco2	28.7 ± 1.7
LS513	28.6 ± 0.6
LS174T	23.8 ± 2.0
HCT15	20.5 ± 1.3
Colo201	20.3 ± 1.4
HCT8	20.1 ± 2.0
LS123	19.8 ± 0.5
LS180	19.1 ± 0.4
DLD-1	18.0 ± 0.1
SW620	16.7 ± 1.4
SW1417	15.1 ± 1.9
SW1463	13.0 ± 1.5
T84	13.0 ± 1.4
RKO	12.3 ± 1.8
SKCO1	12.1 ± 0.1

SW48	10.8 ± 1.5
NCI-H508	9.7 ± 1.2
SW480	9.3 ± 1.7
LOVO	7.8 ± 0.2
HT29	7.0 ± 1.1
SW1116	5.7 ± 0.3
HCT116	4.7 ± 0.2

*The IC_{50} ± s.d. values of MDL-811 to the indicated CRC cell lines were determined by CCK8 assays. The value of each MDL-811 treatment group was calculated as a percentage change of the DMSO controls, which represents the proliferation of the CRC cells. The data represent the mean ± s.d. from two or three independent experiments.

Table S5. Statistical data of cell cycle phase distribution in CRC cell lines treated with MDL-811 for 48 h. [a]

Cell name	Phase	0 μM	5 μM	10 μM
HCT116	G_0/G_1	49.5 ± 3.5	63.2 ± 2.1**	66.9 ± 1.4***
	S	30.1 ± 4.4	19.0 ± 2.3*	19.0 ± 4.3*
	G_2/M	20.4 ± 4.5	17.9 ± 4.0	14.1 ± 3.8
HT29	G_0/G_1	40.5 ± 2.6	54.5 ± 4.1*	59.9 ± 2.7***
	S	36.9 ± 4.6	25.3 ± 4.5	19.6 ± 2.3**
	G_2/M	22.6 ± 6.7	20.2 ± 4.2	20.5 ± 3.7
SW480	G_0/G_1	47.7 ± 1.3	62.9 ± 6.0*	70.0 ± 3.8***
	S	38.3 ± 3.6	26.3 ± 5.1	18.1 ± 4.9***
	G_2/M	13.9 ± 1.8	10.8 ± 4.4	11.9 ± 6.4

[a]The data are shown as mean ± s.d. from three independent experiments (*, $P < 0.05$; **, $P < 0.01$; ***, $P < 0.001$, two-way ANOVA analysis).

Table S6. Primary antibodies used for western blots.

Antibody name	Company name	Catalog number	Dilution
SIRT6	Cell Signaling Technology	12486	1:2000
H3K9Ac	Abcam	ab32129	1:2000
H3K18Ac	Abcam	ab1191	1:2000
H3K56Ac	Active Motif	39281	1:2000
Histone H3	Abcam	ab10799	1:2000
β-actin	Proteintech	HRP-60008	1:5000
His-Tag	Proteintech	HRP-66005	1:10000
Flag-Tag	Sigma-Aldrich	F1804	1:1000

Table S7. Primer sequences used for RT-qPCR.

Gene name	Forward primer (5' to 3')	Reverse primer (5' to 3')
**ACTB**	TGACTGACTACCTCATGAAGATCC	CCATCTCTTGCTCGAAGTCCAG
**LDHA**	AGGAGAAACACGCCTTGATTTAG	ACGAGCAGAGTCCAGATTACAA
**GLUT1**	TGGACCCATGTCTGGTTGTA	ATGGAGCCCAGCAGCAA
**PDK1**	GGAGGTCTCAACACGAGGTC	GTTCATGTCACGCTGGGTAA
**PKM2**	ATGTCGAAGCCCCATAGTGAA	TGGGTGGTGAATCAATGTCCA
**PCNA**	AGGGCTCCATCCTCAAGAAGG	TGGTGCTTCAAATACTAGCGC
**CDC2**	CAGTCTTCAGGATGTGCTTATGC	GAGGTTTTAAGTCTCTGTGAAGAACTC
**CCNA2**	GAAGACGAGACGGGTTGCA	AGGAGGAACGGTGACATGCT
**CDC25C**	GAACAGGCCAAGACTGAAGC	GCCCCTGGTTAGAATCTTCC
**c-MYC**	GGCTCCTGGCAAAAGGTCAGAGT	CTGCGTAGTTGTGCTGATGTGT
**AKT1**	CACAAACGAGGGGAGTACATC	GCCATCATTCTTGAGGAGGAAGT
**AKT2**	TCCAGAACACCAGGCACCC	ATTGTCCTCCAGCACCTCA
**MTOR**	AGTGGACCAGTGGAAACAGG	TTCAGCGATGTCTTGTGAGG
**CYP24A1**	CCGTAATCCCCAAGTGCAAC	CCCAGAACTGTTGCCTTGTC
**SIRT6**	TACGCGGACAAGGGCAAG	ACTTGGGGGCCAGACCTCGC

Table S8. Primary antibodies used for ChIP assays.

Antibody name	Company name	Catalog number	Dilution
SIRT6	Abcam	ab62739	1:200
H3K9Ac	Abcam	ab32129	1:100
H3K18Ac	Abcam	ab1191	1:100
H3K56Ac	Active Motif	39281	1:100
HA-Tag	Cell Signaling Technology	#3724	1:50

Table S9. Primer sequences used for ChIP-qPCR.

Primer name	Forward primer (5' to 3')	Reverse primer (5' to 3')
**H3K9Ac_ 1**	CGCTGTCCCACCTGATCACA	AGGACATGACCGCTTTCTTCAA
**H3K9Ac_ 2**	CTCCCTTTCTCTTTTCCTTTACTCC	ACGCATAACCCCTGTACCCT
**H3K9Ac_ 3**	CAGCTATCCTGAGGTGTGCC	AGGAGCTGTGTCCAGAATTGG
**H3K9Ac_ 4**	TGGGTTCAGGGATTTTGAGGT	TTGCTGGTGATGGGGTGTTC
**H3K9Ac_ 5**	CTAGCGGTAAAAGGGGGCAT	TGAAGCCCACACCAATGAGT
**H3K9Ac_ 6**	TCTCCATGTTCCTATGCCCAG	TCGCTCACCTCGCTGACT
**H3K9Ac_ 7**	CGTAAAGCGGCAACAACGAA	GTAGAAGAACAGAGGCGGGC
**H3K9Ac_ 8**	CCGGATTGCAGAGGAAAGCA	TCCTGGAAGCGGGATCAAAA
**H3K18Ac_ 1**	CGCTGTCCCACCTGATCACA	AGGACATGACCGCTTTCTTCAA
**H3K18Ac_ 2**	TTCAGGTCTTGCTACATCGC	CAGGACGAGGTCATTAGGGG
**H3K18Ac_ 3**	GTCATGTGAGCCCTGGAAGC	GCACGGAGTCAAAGGGAGTT
**H3K18Ac_ 4**	CATACTTCTTGTGGTACTCCACCT	TGGTGCGCTTCGGCGT
**H3K18Ac_ 5**	AATGCACGTAAAGCGGCAAC	TCAAGCATCGTTGGTGCAAG
**H3K18Ac_ 6**	TCTAGGCTGGGCCCTAAATAGT	CTGCCCCACACATACTGACAT
**H3K18Ac_ 7**	CAAGGGACCACCCATGACAA	GCAGAGAAACCAGCCCTTGA
**H3K56Ac_ 1**	CAGCTATCCTGAGGTGTGCC	AGGAGCTGTGTCCAGAATTGG
**H3K56Ac_ 2**	TTTGACTCCGTGCTGGCTAA	GGCATGAAATGACGTGGTGT
**H3K56Ac_ 3**	CATACTTCTTGTGGTACTCCACCT	TGGTGCGCTTCGGCGT
**H3K56Ac_ 4**	CTCTCCATGTTCCTATGCCCAG	CTCGCTGACTCCATCCTCCTTC
**H3K56Ac_ 5**	CGTAAAGCGGCAACAACGAA	GTAGAAGAACAGAGGCGGGC

**H3K56Ac_ 6**	CAAGGGACCACCCATGACAA	GCAGAGAAACCAGCCCTTGA
